# Tracing the origin of disseminated tumor cells in breast cancer using single-cell sequencing

**DOI:** 10.1186/s13059-016-1109-7

**Published:** 2016-12-09

**Authors:** Jonas Demeulemeester, Parveen Kumar, Elen K. Møller, Silje Nord, David C. Wedge, April Peterson, Randi R. Mathiesen, Renathe Fjelldal, Masoud Zamani Esteki, Koen Theunis, Elia Fernandez Gallardo, A. Jason Grundstad, Elin Borgen, Lars O. Baumbusch, Anne-Lise Børresen-Dale, Kevin P. White, Vessela N. Kristensen, Peter Van Loo, Thierry Voet, Bjørn Naume

**Affiliations:** 1 The Francis Crick Institute, London, UK; 2 Department of Human Genetics, KU Leuven—University of Leuven, Leuven, Belgium; 3 Single-cell Genomics Centre, Wellcome Trust Sanger Institute, Hinxton, UK; 4 Department of Genetics, Institute for Cancer Research, Oslo University Hospital, Radiumhospitalet, Oslo, Norway; 5 Cancer Genome Project, Wellcome Trust Sanger Institute, Hinxton, UK; 6 Institute for Genomics & Systems Biology and Department of Human Genetics, University of Chicago, Chicago, IL USA; 7 Present address: Laboratory of Genetics, University of Wisconsin, Madison, WI USA; 8 Department of Oncology, Division of Surgery and Cancer Medicine, Oslo University Hospital, Radiumhospitalet, Oslo, Norway; 9 Present address: Department of Oncology, Akershus University Hospital, Lørenskog, Norway; 10 Department of Pathology, Oslo University Hospital, Radiumhospitalet, Oslo, Norway; 11 Present address: Department of Pediatric Research, Division of Paediatric and Adolescent Medicine, Oslo University Hospital, Rikshospitalet, Oslo, Norway; 12 Department of Clinical Molecular Biology (EpiGen), Medical Division, Akershus University Hospital, Lørenskog, Norway; 13 Institute of Clinical Medicine, University of Oslo, Oslo, Norway; 14 Tempus Labs, Chicago, IL 60654 USA

**Keywords:** Disseminated tumor cells, Single-cell sequencing, Intra-tumor genetic heterogeneity, Phylogeny, Metastasis

## Abstract

**Background:**

Single-cell micro-metastases of solid tumors often occur in the bone marrow. These disseminated tumor cells (DTCs) may resist therapy and lay dormant or progress to cause overt bone and visceral metastases. The molecular nature of DTCs remains elusive, as well as when and from where in the tumor they originate. Here, we apply single-cell sequencing to identify and trace the origin of DTCs in breast cancer.

**Results:**

We sequence the genomes of 63 single cells isolated from six non-metastatic breast cancer patients. By comparing the cells’ DNA copy number aberration (CNA) landscapes with those of the primary tumors and lymph node metastasis, we establish that 53% of the single cells morphologically classified as tumor cells are DTCs disseminating from the observed tumor. The remaining cells represent either non-aberrant “normal” cells or “aberrant cells of unknown origin” that have CNA landscapes discordant from the tumor. Further analyses suggest that the prevalence of aberrant cells of unknown origin is age-dependent and that at least a subset is hematopoietic in origin. Evolutionary reconstruction analysis of bulk tumor and DTC genomes enables ordering of CNA events in molecular pseudo-time and traced the origin of the DTCs to either the main tumor clone, primary tumor subclones, or subclones in an axillary lymph node metastasis.

**Conclusions:**

Single-cell sequencing of bone marrow epithelial-like cells, in parallel with intra-tumor genetic heterogeneity profiling from bulk DNA, is a powerful approach to identify and study DTCs, yielding insight into metastatic processes. A heterogeneous population of CNA-positive cells is present in the bone marrow of non-metastatic breast cancer patients, only part of which are derived from the observed tumor lineages.

**Electronic supplementary material:**

The online version of this article (doi:10.1186/s13059-016-1109-7) contains supplementary material, which is available to authorized users.

## Background

Cancer is a disease of the genome, arising through the accumulation of somatic driver mutations, leading to successive clonal expansions [1, 2]. Somatic mutations can take many forms, including single nucleotide changes, small insertions and deletions, genomic rearrangements, copy number aberrations, and epigenetic changes. While the majority of these mutations are innocent passenger mutations, a small subset are drivers, conferring a selective advantage on the cells that carry them and sparking clonal expansions. Cancers develop through Darwinian and punctuated evolutionary processes in which early clonal expansions represent complete selective sweeps [3]. As a result of these early clonal expansions, the driver and passenger mutations in the originating cells are inherited in all cancer cells. The cell of origin that prompts the last complete selective sweep can be termed the most recent common ancestor (MRCA), the one cell from which all cancer cells in a tumor sample derive. Later driver mutations may result in incomplete clonal expansions, resulting in a patchwork of genetically related but competing subclones. In breast cancer, assessment of variant allele frequencies in bulk DNA samples has allowed determination of the subclonal architecture of the tumor [4, 5]. Parallel advances in single-cell isolation, DNA amplification, and computational approaches have recently enabled single-cell genome sequence analyses, providing unprecedented power to dissect intra-tumor genetic heterogeneity [6–11].

Cancer cells may intravasate from a solid tumor, travel through the blood stream as a circulating tumor cell, and subsequently extravasate in distant organs like the bone marrow. These disseminated tumor cells (DTCs) in the bone marrow may remain dormant for years, providing a reservoir of progenitors for distant metastases [9, 12]. Patients diagnosed with non-metastatic breast cancer still have a significant risk of relapse, even after complete surgical removal of the tumor, most likely due to the existence of DTCs, reported in up to 40% of cases [13, 14]. Their presence in bone marrow aspirates at the time of diagnosis or following treatment is a prognostic marker for poor survival [13–19]. DTCs can be refractory to therapy due to their dormant cell state or other cellular features, such as overexpression of the *Her2* proto-oncogene [20, 21]. The concentration of DTCs in the bone marrow is typically estimated at one cell per 10^7^–10^8^ blood cells in patients with advanced disease [13]. These cells are usually identified using immunocytochemistry or immunofluorescence for epithelial (e.g., cytokeratins, EpCAM) or breast tissue markers (e.g., human mammaglobin) [13].

Exactly when and where DTCs arise during tumor evolution, as well as the molecular mechanisms involved, remain largely elusive. Two main models have been proposed for dissemination of tumor cells [22]. The parallel progression model hypothesizes that cancer cells leave their site of origin early, resulting in largely independent evolution of the primary tumor and the disseminated cells. Under this model, the primary tumor and DTCs can present with profoundly different genomes. In contrast, the linear model proposes a sequential process whereby tumor cells disseminate from major or minor subclone(s), leading to at least partly identical genomic profiles for DTCs and the primary tumor.

Previous genomic analyses of cells, immunocytochemically classified as DTCs in bone marrow aspirates, primarily employed comparative genomic hybridization. In patients with non-metastatic breast cancer, the majority of identified cells displayed either a normal euploid profile or an aberrant DNA copy number landscape seemingly unrelated to the primary tumor [23, 24], suggesting parallel evolution. Additionally, copy number aberrations (CNAs) detected among DTCs from the same non-metastatic patient were generally non-recurrent [23, 25]. In contrast, DTCs isolated from the same patient burdened with metastatic disease frequently shared CNAs, also with the primary tumor and/or the lymph node metastasis, fitting a linear progression model [26, 27].

In this study, we applied single-cell sequencing to profile the genomic landscape of cells isolated based on immunocytochemical and morphologic parameters from bone marrow aspirates of six breast cancer patients. We correlated their profiles with the (sub)clonal CNA architectures and somatic single nucleotide substitution profiles—obtained by SNP-array and exome-sequencing, respectively—of the primary tumors as well as one lymph node metastasis. Copy number and somatic single nucleotide variant genotyping analyses reveal that only a fraction of the cells commonly selected as DTCs from bone marrow aspirates in breast cancer derive from the same lineage as the observed tumor clones. The cells exhibiting copy number neutral or aberrant profiles dissimilar from that of the primary tumor do not derive from the observed primary tumor. Additionally, by combining single-cell sequencing with subclonal reconstruction of the bulk tumor, we construct detailed phylogenetic trees of the breast cancers and trace the origins of the genuine DTCs. Our results support a model where tumor cells disseminate relatively late, from observable subclones in the primary tumor or metastasis.

## Results

### Immunocytochemical and sequencing-based molecular classification of single cells in the bone marrow

Following the established immunocytochemical staining for putative DTCs (“Methods”) [17, 28–30], we isolated 56 single cells from seven bone marrow aspirates of six breast cancer patients (six aspirates taken at diagnosis and one taken 3 years after; Additional file 1: Figure S1; Additional file 2: Table S1). We also isolated seven control cells after staining each sample with an isotype control monoclonal antibody (mAb) instead of the anti-cytokeratin mAbs. Based on previously described morphologic parameters, the single cells were classified as tumor cell (TC), probable hematopoietic cell (PHC), hematopoietic cell (HC), or uncertain cell (“Methods”) [29]. The patients included in this study had not previously been diagnosed with any type of cancer and had localized or regional disease and no distant metastasis at diagnosis. Four of the six patients were diagnosed with invasive lobular carcinoma and the remaining two with invasive ductal carcinoma (Table 1). All patients had grade 2 tumors; five had hormone receptor-positive tumors with confirmed axillary lymph node involvement. Two patients had Her2-positive tumors. Four patients developed systemic recurrence, three of which died of breast cancer and one was still alive at last follow-up 241 months later. Two patients died of other causes. For all patients, putative DTCs were collected at diagnosis as well as a sample from the primary tumor. For one patient, MicMa107, a synchronous axillary lymph node metastasis was sampled as well, and DTCs were obtained again 3 years after diagnosis (Additional file 2: Table S1). The bulk primary and lymph node samples were subjected to SNP array profiling and whole exome sequencing.

**Table 1:** Clinical parameters of the six breast cancer patients

Patient	Histology	Grade	pN status	HR status	Her2 status	PAM50 subtype	Systemic recurrence (time to recurrence or last observation)	Age at diagnosis (years)	Time to death (months)	Disease status
MicMa003	ILC	2	2	Pos	Neg	LumA	Yes (11.71 months)	36	-	Alive (241 months)
MicMa017	ILC	2	1	Pos	Neg	LumA	No (last obs 80.98 months)	78	80.98	Dead other cause
MicMa019	IDC	2	2	Pos	Neg	LumB	Yes (40.31 months)	71	52.56	Dead BrCa
MicMa044	IDC	2	ND	Neg	Pos (FISH)/neg (IHC)	Basal	No (last obs 8.26 months)	81	8.26	Dead other cause
MicMa083	ILC	2	3	Pos	Neg	LumA	Yes (11.21 months)	69	19.37	Dead BrCa
MicMa107	ILC	2	3	Pos	Neg (FISH)/pos (IHC)	LumB	Yes (44.31 months)	45	55.10	Dead BrCa

*BrCa* breast cancer, *ER* estrogen receptor, *FISH* fluorescence in situ hybridization, *HR* hormone receptor, *IDC* invasive ductal carcinoma, *IHC* immunohistochemistry, *ILC* invasive lobular carcinoma, *LumB* luminal B, *ND* not determined, *Neg* negative, *Obs* observation, *pN* axillary lymph node involvement, *Pos* positive

**Fig. 1:**
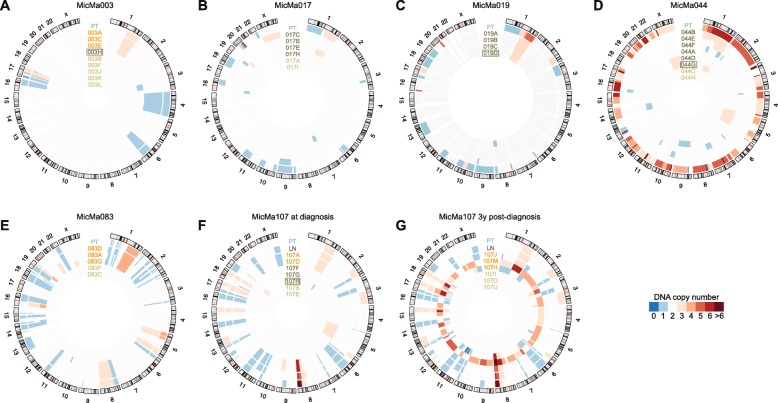
DNA copy number profiles of breast tumors and bone marrow-derived single cells. **a**–**g** Per patient profiles are shown as *concentric circles* inside the circular human karyogram. Total (clonal) copy number is represented as a heat map from *blue* to *red* as indicated. Tumor samples and single cells are labeled on the corresponding circles and are color-coded: primary tumor bulk (*PT*) in *cyan*, lymph node bulk (*LN*) in *purple*, DTCs in *orange*, aberrant cells of unknown origin in *dark green*, and normal cells in *green*. Cells isolated after MOPC 21 isotype control staining are *boxed*. Panels are shown for patient MicMa003 (**a**), MicMa017 (**b**), MicMa019 (**c**), MicMa044 (**d**), MicMa083 (**e**), and MicMa107 at the time of diagnosis (**f**) and 3 years post-diagnosis (**g**)

We amplified the genomes of these 63 single cells using a modified GenomePlex approach (“Methods”) and performed low coverage paired-end sequencing, resulting in 1.7× (±1.2×) coverage depth (average ± standard deviation) and 23.7% (±15.4%) coverage breadth across the cell’s genome (Additional file 2: Table S2). Following read depth analysis, we observed different relationships of the DNA copy number profiles of the cells to the clonal copy number profiles of the bulk primary tumors and lymph node metastasis as assessed by SNP array (Table 2, Fig. 1). Eleven single cells presented with CNAs similar to the primary tumor or the lymph node metastasis and were classified as genuine tumor-derived DTCs. Other single cells, however, demonstrated either copy neutral profiles or CNAs that were distinct from the bulk tumor. These cells were labeled, respectively, as “normal” cells (N; *n* = 16) and “aberrant cells of unknown origin” (AU; *n* = 24) (Table 2, Fig. 1; Additional file 2: Table S3). Six of the aberrant cells, including one control, and five of the normal cells exhibited increased noise in the read depth profiles, which may induce false positive CNAs (“Methods”; Additional file 1: Figure S2). The aberrant cells with increased noise were excluded from our analyses as they could not be unambiguously assigned to the N or AU category. In a few cases, a CNA in an AU cell overlapped a CNA in the corresponding bulk tumor. However, in all cases where the breakpoints were identifiable (i.e., not at a centromere or a telomere), they were demonstrably distinct, indicating that these represented independent CNA events (Additional file 1: Figure S3). Additional investigation of the micrographs of each cell along with their respective CNA profile revealed that in about one isolation out of five, two or more cells were likely collected rather than just one (Additional file 1: Figures S1, S4 and S7; Additional file 2: Table S3). These were annotated as “doublets” (D; *n* = 12, including two control cells), several of which are likely to contain a tumor-derived DTC based on their CN landscape (e.g., 003D, 107N).

**Table 2:** Classification of the single cells isolated from bone marrow following sequencing

Patient	Collection time	DTC	Normal	Aberrant cell of unknown origin	Doublet	Total
MicMa003	Time of diagnosis	3	5	1	3	12
MicMa017	Time of diagnosis	-	2	4	3	9
MicMa019	Time of diagnosis	-	-	4	-	4
MicMa044	Time of diagnosis	-	2	6	-	8
MicMa083	Time of diagnosis	3	2	3^a^	1 triplet	9
MicMa107	Time of diagnosis	2	2	1^a^ + 3	1	9
	Three years after diagnosis	3	3	2^a^	4	12
Total		11	16	24	12	63

^a^These aberrant cells were excluded from downstream analyses based on genome-wide noisy logR profiles (see also Additional file 1: Figure S2 and Additional file 2: Table S3)

To assess whether generalizable conclusions can be drawn from the remaining 45 single cells, we performed a retrospective power analysis [31]. Specifically, we plotted the cumulative number of unique CNAs as a function of the cumulative number of single cells (Additional file 1: Figure S5). In those patients where genuine DTCs were identified, the curve consistently flattens off, indicating that sufficient cells have been sequenced to capture the diversity of the existing DTC population. In MicMa107, the 3 year after diagnosis time point has a much steeper rarefaction curve compared to the one at diagnosis, suggesting that additional samples are more likely to reveal novel clones.

Most of the 11 single cells categorized by sequencing as DTCs were classified by morphology and staining as TCs (n = 10), while the remaining single cell was interpreted as a HC (*n* = 1) (Table 3). The 18 AU cells, encompassing all four remaining control cells, were morphologically and immunocytochemically classified as TC (*n* = 3), uncertain (*n* = 4), PHC (n = 8), or HC (*n* = 3), while the 16 N cells were typed as TC (*n* = 6), uncertain (*n* = 5), or PHC (*n* = 5). Six cells were consistently classified as N/HC after morphology and CNA analyses. In summary, the seven control cells represented five AUs, one of which was excluded from further analyses, and two Ds. The 56 isolated anti-cytokeratin stained cells constituted 11 genuine DTCs, 19 AUs (five of which were excluded), 16 Ns, and 10 Ds.

**Table 3:** Single-cell sequencing-based classification versus morphologic classification

		Morphologic classification				
		TC	Uncertain	PHC	HC	Total
Sequencing-based classification	DTC	10	-	-	1	11
	AU	3	3 (+1)	7 (+1)	1 (+2)	14 (+4)
	N	6	5	5	-	16
	Doublets	5^a^	3^a^	1^a^	1 (+2)	10^a^ (+2)
	Total	24	11 (+1)	13 (+1)	3 (+4)	51 (+6)

^a^Some of these doublets contain a DTC (see also Additional file 1: Figures S7 and S8 and Additional file 2: Table S6). Control cells are counted between brackets. *DTC* disseminated tumor cell, *AU* aberrant cell of unknown origin, *N* normal cell, *TC* tumor cell, *Uncertain* uncertain cell, *PHC* probable hematopoietic cell, *HC* hematopoietic cell.

### Somatic single nucleotide substitutions in the bulk tumor exome and single-cell sequences

**Fig. 2:**
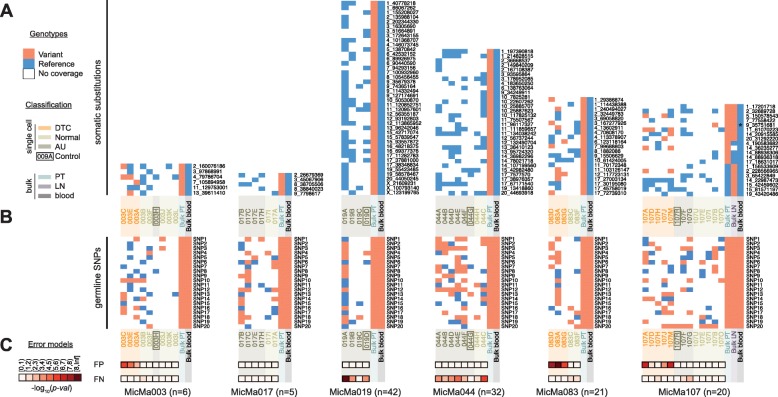
Genotyping of single nucleotide variants from bulk tumor exome sequences in the single-cell sequences. **a**, **b** Heat maps per tumor, where each *row* represents either a single somatic substitution called on the corresponding bulk exome and the matched blood (**a**) or a random heterozygous germline SNP (20 total) (**b**), and *columns* represent the different single cell (DTC, normal, or AU) or exome datasets obtained for that tumor. *Tile colors* reflect the detection of the variant allele (*orange*), of the reference allele only (*blue*), or whether there was no coverage at that position (*white*). Only DTCs, but no normal or AU cells, share mutations with the tumor. A single mutation shared between normal cell 107B and the tumor of MicMa107 was later confirmed as a missed heterozygous germline variant (indicated with an *asterisk*). For clarity, loci with zero coverage in all of the single cells of that patient are omitted. **c** Modeled probability of observing an at least equally extreme pattern of somatic reference and variant alleles for that cell only through false positives (i.e., the cell derives from another lineage and has none of the tumor’s somatic mutations) or false negatives (the cell derives from the tumor and contains these specific somatic mutations). Model results are encoded as heat maps of − log_10_(*p*)

To further investigate the origins of the cells, we sequenced the bulk exome of the primary tumor and matched normal blood for all patients, as well as the lymph node metastasis of patient MicMa107. The average depth and breadth of coverage reached (34.9× and 83% of the targeted region, respectively; Additional file 1: Figure S6) was sufficient to identify somatic single nucleotide substitutions. Following filtering of the variants called by MuTect (“Methods”), we retained 239 somatic substitutions in the seven bulk cancer exomes (Additional file 2: Tables S4 and S5), of which 103 are non-synonymous, four are nonsense, 40 are synonymous, and 92 are present in introns or intergenic regions. These included driver mutations in *ERBB2* in patient MicMa019 and in *PIK3CA* and *TP53* in patient MicMa044. Subsequently, we genotyped the substitutions in the sparser single-cell data: 117 of the 239 positions were covered by at least one read in at least one cell (Fig. 2; Additional file 2: Table S4). The DTCs of patient MicMa003 displayed four out of six covered tumor bulk somatic substitutions, those of MicMa083 displayed ten out of 21, and those of MicMa107 displayed six out of 20. Conversely, the AU and N cell data did not contain a single read reporting any of the 117 somatic substitutions. Note that one normal cell (107B) from patient MicMa107 appeared to contain a single somatic substitution (Fig. 2). However, by PCR-based genotyping in the primary tumor and blood sample, we confirmed this substitution to be a germline SNP that was miscalled as a somatic variant due to low coverage in the matched normal sample.

The occurrence of single-cell sequence reads reporting a substitution known from the tumor bulk provides strong evidence for its presence in the single cell despite false positive substitutions that occasionally result from whole-genome amplification (WGA) or sequencing artifacts (the false positive rate over all single cells was 0.0017 ± 0.0026). The detection of multiple somatic substitutions in most DTCs further substantiates that they indeed originated from the patients’ tumors. In contrast, the sparse coverage, together with the possibilities of complete locus dropout and allelic dropout, do not allow us to ascertain whether a somatic substitution is absent from a single cell (locus dropout rate 0.6907 ± 0.1485, false negative rate 0.4405 ± 0.1495). However, the consistent lack of single-cell reads reporting any mutant allele across all bulk somatic substitutions in the AU and N cells suggests that they do not share a lineage with the observed breast tumor (Fig. 2; Additional file 2: Table S4). As opposed to the somatic substitutions, which were undetectable in the AU and N cells, detection rates of alternative alleles of germline SNPs (Number of alternative/Total number covered) were similar across all single cells, irrespective of their classification (Fig. 2, “Methods”).

To quantify the confidence which the genotyping results provide regarding single cell origins, we calculated per-cell false positive and false negative error rates of genotyping germline homozygous and heterozygous SNPs, respectively. Using these estimates as proxies for the corresponding error rates of genotyping somatic single nucleotide substitutions, we modeled the pattern of reference and variant somatic alleles per cell as a beta-binomial distribution. DTCs generally have *p* values ≤10^−2^ for the false positive model, indicating that it is unlikely they do not have any of the tumor’s somatic single nucleotide substitutions, i.e., they likely derive from the tumor lineage. Vice versa, where we have the power (enough somatic variants and coverage, e.g., MicMa019 and 044), the false negative error model shows that the AU and N cells are unlikely to share the tumor’s single nucleotide substitutions, i.e., they most likely derive from another lineage.

Along with inspection of their CNA landscapes, genotyping somatic variants in the doublets allowed us to build confidence and classify at least one of the constituent cells (Additional file 1: Figures S7 and S8). The doublets of MicMa003 include another two DTCs (one with genotyping evidence) and at least one AU. Those of MicMa017 comprise no less than three AUs, and those of MicMa107 contain four DTCs (two with genotyping evidence) and at least one AU (Additional file 2: Table S6). Taken together, tumor bulk and single-cell mutation analysis corroborated the CNA-based classification of the cells. Including cells captured in doublets, we observed five DTCs from patient MicMa003, three DTCs from patient MicMa083, and nine DTCs from patient MicMa107. In all patients, the cells identified as N or AU did not share any somatic mutations (neither CNAs nor single nucleotide substitutions) with the bulk tumor samples and are therefore unlikely to share a breast tumor lineage.

### Lineage of the aberrant cells of unknown origin

**Fig. 3:**
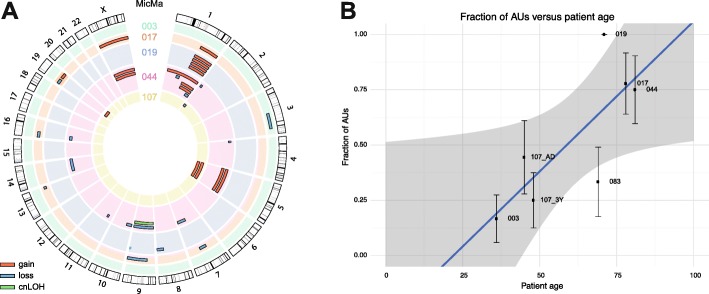
Aberrant cells of unknown origin show recurrent aberrations and correlate with age. **a** Gains (*red*), losses (*blue*), and copy neutral loss of heterozygosity (*cnLOH*, *green*) events observed in the AUs of the different patients. **b** Linear regression analysis of the fraction of aberrant non-DTCs versus patient age. *Shaded areas* represent the regression 95% confidence interval and *error bars* the standard error of the estimated proportion. All cells (doublet constituents and quality control-failed) were taken into account to estimate the fractions

Although the immunocytochemical staining of AUs (and Ns) suggests that they derive from an epithelial lineage, false positive staining cannot be ruled out. Specifically, plasma cells directly reactive to alkaline phosphatase have previously been found to contribute false positive results [32]. Interestingly, the four control single cells, stained using the isotype control mAb instead of anti-cytokeratins, were classified as AUs. Since most of these cells were also morphologically classified as HCs, it is likely that at least a subset of all AUs derives from the hematopoietic lineage. Looking across AUs, 44% of the cells show gains on 1q and 22% have a gain of chromosome 5 (Fig. 3a). While the former is commonly observed in various cancer types, the latter is rare in breast cancer but more frequent in liver cancer and multiple myeloma, a cancer of plasma cells (Mitelman Database of Chromosome Aberrations and Gene Fusions in Cancer, http://cgap.nci.nih.gov/Chromosomes/Mitelman). Several AUs within patients share copy number aberrations, hinting at an ongoing process of clonal expansion and evolution of some of these cells: 1q gain in 019A-D, chromosome 5 gain and chromosome 9 loss or copy neutral loss of heterozygosity in 044A and 044D, respectively, and chromosome 5 gain in 107F and 107G (Figs. 1 and 3a). In line with this observation, the fraction of AUs over total cells tends to increase with patient age (Fig. 3b, slope = 0.013 ± 0.004, *p* = 0.026). In summary, the classification of control cells, the typical and recurrent CNA landscapes, and the correlation with patient age suggest that at least part of the AUs derive from clonally expanding hematopoietic cell populations.

### Tracing the origin of disseminated tumor cells in breast cancer

**Fig. 4:**
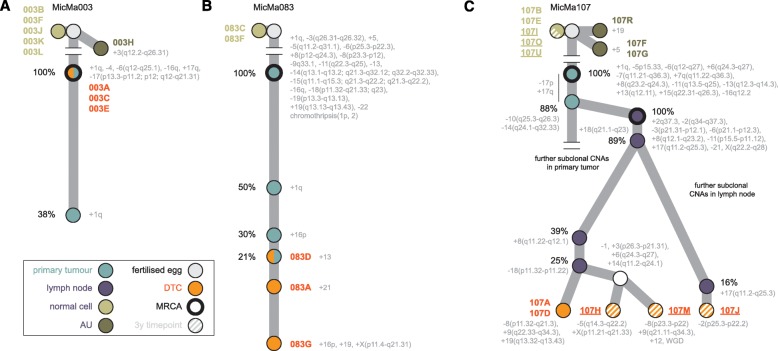
Tracing the origins of DTCs in the breast cancer phylogenetic trees. Copy number-based phylogenetic trees drawn up for patients MicMa003 (**a**), MicMa083 (**b**), and MicMa107 (**c**). Nodes in the trees correspond to (sub)clones and are color-coded based on their type as indicated (primary or lymph node, DTC, AU, or normal cell). *Grey nodes* are not observed directly but can be inferred from the data. *Nodes* are annotated with their specific CNA event and, where possible, with their estimated cancer cell fraction (the percentage of tumor cells containing the indicated aberration). Within the tumors, branch lengths reflect differences in cancer cell fraction. The most recent common ancestor (*MRCA*) in each bulk sample is indicated with a thicker stroke. For MicMa107, single cells isolated 3 years post-diagnosis are represented as *striped nodes*. DTC 107M has undergone a whole-genome duplication (*WGD*)

To evaluate the existing cancer progression models and provide insight into breast tumor evolution, we next reconstructed the phylogeny of the tumors in patients MicMa003 and MicMa083 and traced the origins of their DTCs. To this end, we compared the CNA landscape of the DTCs with the (sub)clonal CNA architecture of the corresponding primary tumors, as determined by the Battenberg algorithm [4] (Fig. 4; Additional file 1: Figures S8 and S9). In both patients, the cancer exhibited a linear progression model. Specifically, in patient MicMa003, the bulk primary tumor contained clonal 1q and 17q DNA gains as well as a loss of chromosome 4 and segmental deletions -6(q12-q25.1), -16q, and -17(p13.3-p11.2; p12; q12-q21.31). A further subclonal gain of the 1q-arm was detected in 38% of tumor cells, demonstrating ongoing evolution. The DTCs from this patient (003A, 003C, and 003E) shared all clonal CNAs but lacked the subclonal 1q gain or any private CNAs, supporting their origin from the most recent common ancestor (MRCA) and not from the more recent subclone (Fig. 4a; Additional file 1: Figure S8a and S9a, b).

In patient MicMa083, DTCs 083A/D/G shared all clonal CNAs from the primary tumor as well as the gains of 1q, 16p, and chromosome 13, which were subclonal in the primary, occurring in 50, 30, and 21% of the tumor cells, respectively (Fig. 4b; Additional file 1: Figure S8e, S9c–f). The presence of these aberrations in the DTCs allowed us to confidently nest the different subclones within one another and derive a linear tree. DTCs 083A and 083G demonstrated further aberrations, not observed in the primary tumor. Both cells have an additional trisomy of chromosome 21, but only 083G has gained another copy of 16p, chromosome 19, and X(p11.4-q21.31). These results show that the primary breast cancers of patient MicMa003 and MicMa083 both display linear evolution. DTCs either disseminated from the MRCA observed in the primary (MicMa003) or from subclones in the primary (MicMa083) that either did (083A) or did not (083D) evolve further.

### Tumor cell dissemination from axillary lymph node metastasis subclones

We subsequently used similar principles to reconstruct the evolutionary history of the primary tumor, axillary lymph node metastasis, two DTCs harvested at diagnosis, and three DTCs harvested 3 years after diagnoses in patient MicMa107. This revealed a complex progression model with DTCs originating from multiple subclones in the lymph node metastasis (Fig. 4c; Additional file 1: Figure S8f, g and S9g–o). The primary tumor and axillary lymph node metastasis exhibited distinct but clearly evolutionarily related CNA profiles. While the MRCA of the primary tumor contained CNAs on chromosomes 1, 5, 6, 7, 8, 11, 13, 15, and 16, the “MRCA” of the lymph node metastasis had several additional aberrations (Fig. 4c; Additional file 1: Figure S8f, g and S9g–o). Interestingly, the CNAs -10(q25.3-q26.3), -14(q24.1-q32.33), and potentially also -17p and +17q were clonal in the lymph node metastasis but define a subclone of 88% of cancer cells in the primary tumor. Furthermore, in the primary tumor, Battenberg identified a large number of CNAs present in smaller subclones, suggesting substantial intratumor heterogeneity. The axillary lymph node metastasis also showed extensive subclonal variegation (Fig. 4c; Additional file 1: Figure S8f, g and S9g–o; Additional file 2: Table S7), with many CNAs absent or below the detection limit in the primary tumor. Nevertheless, independent but similar subclonal CNAs were present in the primary and lymph node metastasis on 1q, 2q, 8q, and 18q, suggesting convergent evolution. Notably, the level of amplification of 8q, possibly driven by *MYC*, seemed to vary between subclones and DTCs. Subclonal CNAs +18(q21.1-q23), +8(q11.22-q12.1) and -18(p11.32-p11.22) were detected in 89, 39 and 25%, respectively, of the cancer cells in the lymph node metastasis as well as in the single-cell sequences of the two DTCs isolated at the time of diagnosis, 107A and 107D. Coexistence of these subclonal CNAs in both DTCs together with all clonal CNAs unique to the lymph node metastasis allowed us to resolve the phylogenic tree by nesting the three subclones within one another under the lymph node MRCA. DTCs 107A and 107D contained additional DNA gains, +9(q22.33-q34.3) and +19(q13.32-q13.43), and a loss of part of a previously gained segment, -8(p11.32-q21.3), not observed in the bulk data. Hence, these two DTCs disseminated to the bone marrow only late, from a small subclone in the axillary lymph node metastasis and not from the primary tumor.

### The population of disseminated tumor cells 3 years after diagnosis

The DTCs isolated 3 years post-diagnosis (107H, 107J, and 107M) presented all clonal aberrations of the lymph node metastasis, as well as the subclonal +18(q21.1-q23) gain, showing that also these cells did not directly derive from the primary tumor. They did not share the private CNAs of 107A and 107D. DTC 107J additionally contained a gain, +17(q11.2-q25.3), and a loss, -2(p25.3-p22.2). While the latter was private, the former was detectable as a subclonal CNA in 16% of the lymph node cancer cells at the time of diagnosis. DTCs 107H and 107M were more closely related to the DTCs isolated at the time of diagnosis and appear to be derived from the same 25% subclone in the lymph node, sharing all of its CNAs. Cells from this subclone seem to have evolved further, acquiring the unique gains +3(p26.3-p21.31), +6(q24.3-q27), and +14(q11.2-q24.1) as well as a loss of chromosome 1. DTC 107H showed an additional gain, +X(p11.21-q21.33), and loss, -5(q14.3-q22.2), while DTC 107M was likely tetraploid with additional gains of 9(q21.11-q34.3) and chromosome 12, and an 8(p23.3-p22) loss. The large number of new CNAs identified in these three DTCs highlights ongoing processes of chromosomal instability and subclonal evolution during follow-up. Indeed, the steeper rarefaction curve derived for the sample 3 years after diagnosis compared to the sample at diagnosis confirms that the population of DTCs in MicMa107 has diversified over time (Additional file 1: Figure S5). In summary, these results show that all DTCs isolated from patient MicMa107 are descendants of subclones in the axillary lymph node metastasis, which have survived therapy and continued to evolve.

## Discussion

Bone marrow provides a reservoir for DTCs that may evade therapy, remain dormant, and can cause overt metastases over time [9]. Hence, developing our understanding of the origin, nature, and biology of this cancer cell “type” is important. In contrast to previous studies that primarily applied low-resolution metaphase-chromosome or microarray comparative genomic hybridization, we employed single-cell genome sequencing to shed new light on the cells isolated as “DTCs” from bone marrow aspirates according to established immunocytochemical and morphologic criteria. Specifically, we demonstrate the existence of three major classes among the isolated cells: (i) true DTCs, which share both CNAs and single nucleotide substitutions with the tumor; (ii) aberrant cells of unknown origin which have CNA profiles that do not match those of the observed tumor or metastasis and that lack the somatic substitutions of the tumor; and (iii) normal cells having copy neutral genomes without tumor-specific mutations.

Of the 19 single cells immunocytochemically and morphologically classified as TCs, ten could be conclusively categorized as true DTCs (14 out of 24 including doublets). In contrast, only one of the 21 single cells classified as uncertain, PHC, or HC turned out to be a true DTC (three out of 26 including doublets). While the adaptation of the staining—as required for downstream genomic analysis [26]—may have affected the precision of the morphologic classification of the immuno-detected single cells, these findings still underscore its value (true positive rate 52.6 ± 11.5%, false negative rate 4.8 ± 4.7%). Notably, we found true DTCs for only three patients (MicMa003, 083 and 107), all of which went on to develop distant metastases (Table 1). In contrast, only AU and N cells were found in MicMa017, 019, and 044, of which only MicMa019 showed systemic recurrence.

Our results suggest that previous studies based on morphologic criteria may have been underrepresented for “true DTCs”, as defined here by genetic profiling. Cells identified in those studies, usually from patients without distant metastases, carrying a smaller number of chromosomal aberrations [23, 25, 33] distinct from one another and from those in the tumor under consideration [23, 33], and usually consisting of whole-chromosome gains or losses [23], likely correspond to AUs. These cells may have been interpreted as genuine DTCs, thus supporting a parallel progression model of the disease. The idea has been put forward that AU-like cells, in their ectopic site, obtain a genomic landscape similar to the primary tumor in a macroevolutionary process resulting from evolutionary shifts as could be induced by telomere crisis or the inactivation of a tumor suppressor such as *TP53* [34]. To the best of our knowledge, we provide the first comprehensive investigation with modern sequencing technologies of these cells. Our data show a complete absence of tumor-specific truncal mutations in the AUs, supporting the notion that these cells do not derive from the MRCA nor any other observable progenitor of the sampled breast tumor. Indeed, direct evidence implicating AU-like cells as the precursors of overt metastases is scant [35].

The AUs (and perhaps also the normal cells) in our study may either represent a likely epithelial cell type of breast or non-breast origin homing to the bone marrow or derive from the hematopoietic cell lineage. Alternatively, these cells may originate from another neoplasm in the patient, an undetected synchronous primary breast tumor, or an undetectable or unsampled tumor cell clone residing in the primary tumor. Our results suggest that at least part of the AUs derive from hematopoietic cell populations, most likely plasma cells. Interestingly, studies employing single nucleus sequencing have previously reported cells with random gains or losses of single chromosomes or chromosome arms in diploid genomes, similar to many of the AUs [6, 36, 37]. These “pseudodiploid cells” were observed at rates of 1–6% in different normal tissues (brain, liver, skin, and breast) and 6–8% in breast tumor stroma, suggesting that even normal tissues display low-level aneuploidy, likely due to mitotic segregation defects. While pseudodiploid cells may account for about half of the AUs, those harboring recurrent CNAs (e.g., gain of chromosome 1q or 5 and loss of 9) are more likely to represent clonal expansion of a (pre)malignant subpopulation. At least two studies using SNP-array analyses of bulk DNA have now documented the emergence of subclonal hematopoietic cell lineages containing large CNAs within the blood of cancer cases as well as cancer-free controls [38, 39]. Like the fraction of AUs, the frequency of this subclonal genetic mosaicism increased with age in cancer-free individuals. By using single-cell sequencing, we may be witnessing the diversity of genomically aberrant hematopoietic cells that exist in low numbers within the bone marrow.

Using the CNAs as a guide, true DTCs were mapped onto the phylogenetic trees of the breast cancers, inferred by Battenberg from the bulk tumor DNA, exposing their evolutionary origins. The single-cell genome sequences enabled us to add considerable detail to the phylogeny of the solid tumors of patients MicMa083 and MicMa107. For patient MicMa083, the 50, 30, and 21% primary tumor subclones could only be nested confidently within one another because the single-cell DTC sequences reported coexistence of all three CNA events. Without knowledge of co-occurrence of these rare CNAs in the same cell, it would have been impossible to order these events in molecular pseudo-time. Similarly, the phylogenetic tree of the lymph node metastasis in patient MicMa107 could be reconstructed in more detail owing to the single-cell DTC sequences.

Our results provide clear insights into the origin of DTCs in breast cancer. All true DTCs in this study derive from either the MRCA (MicMa003), subclones of the MRCA (MicMa083), or subclones in an axillary lymph node metastasis that was seeded by a subclone of the MRCA of the primary tumor (MicMa107). We did not observe any DTC that carried only a subset of somatic changes present in the MRCA of the primary tumor, which would point towards early dissemination. Taken together, these results support a model whereby the ability of breast cancer cells to disseminate to the bone marrow arises relatively late in tumor evolution. However, we cannot rule out the possibility that continuous seeding and replacement is occurring: early disseminating tumor cells are replaced by new arrivals from the tumor as they compete for a bone marrow niche.

Interestingly, in patient MicMa083, all DTCs originated from the same 21% subclone. In MicMa107, however, multiple subclones hold the ability to disseminate to the bone marrow. Notably, only subclonal populations in the lymph node metastasis that have undergone considerable evolution since the MRCA are observed in the bone marrow. Hypothetically, metastatic potential (or the ability to disseminate to the bone marrow as a proxy to that) emerged with the MRCA in MicMa003 and significantly later in MicMa083 and MicMa107. While our sample size is small and further study is needed, these results raise the hope that early detection strategies can lead to diagnosis and treatment before the emergence of such metastatic clones.

## Conclusions

Single-cell sequencing of cells isolated from bone marrow aspirates of patients with localized breast cancer, in parallel with intratumor genetic heterogeneity profiling of the bulk tumor DNA, is a powerful approach for the identification of genuine DTCs and the subsequent study of their biology and tumor progression. Additionally, the single-cell sequences of the DTCs help resolve the phylogenetic tree structure by evidencing co-occurrence of rare subclonal CNAs—presenting with different low allelic frequencies in the bulk—in the same single cell. Finally, our data uncover a heterogeneous class of CNA-positive cells isolated as DTCs but unrelated to the breast cancer lineage, warranting further exploration.

## Methods

### Patient material

Primary tumor tissue and bone marrow aspirates were obtained from six distant metastasis-free breast cancer patients included in the Oslo Micrometastasis (MicMa) project [18]. For one patient we also retrieved a lymph node metastasis biopsy. Biopsies were fresh frozen at −80 °C. Clinical classification of the patients and tumors is described in Additional file 2: Table S1. DNA was extracted using an ABI 341 Nucleic Acid Purification System (Applied Biosystems, Carlsbad, CA, USA). Bone marrow aspirates were taken at the time of surgery, and, for one patient, also 3 years after diagnosis.

### DTC isolation, detection, and classification

The DTC isolation and detection procedures have been described in detail previously [26]. In brief, after bone marrow mononuclear cell isolation and cytospin preparation, single cells were analyzed by immunocytochemical staining either for cytokeratins using the AE1/AE3 mAb combination, which covers a broad spectrum of cytokeratins (CK10, 14–16, 19 (AE1) and CK 1–8 (AE3)) or the MOPC 21 isotype negative control mAb, and the standard APAAP (Dako, Denmark) detection system [26]. The cytospins were screened manually using light microscopy, and detected immuno-stained cells were morphologically classified according to a current standardized and clinically validated procedure into four groups: tumor cell (TC), uncertain cell, probable hematopoietic cell (PHC), and hematopoietic cell (HC). Cells with a clearly enlarged nucleus compared with the neighboring HCs are classified as TC, in addition to cells appearing in clusters. Also, cells lacking these pathognomonic TC features and having HC-sized nuclei may be classified as TC if no recognizable hematopoietic features can be identified. Most often such cells show strong and irregularly stained cytoplasm, often partly covering the nucleus, and an atypical appearance. On the other hand, typical HC features are low nuclear/cytoplasmic ratio, with nuclear size similar to that of the neighboring bone marrow cells, weak or moderate staining intensity, and an evenly stained cytoplasm, often with microvacuolization; some have a recognizable plasma cell appearance. The detected cells, however, cover a continuous morphologic spectrum, with overlapping features between TC and HC. We have here classified as PHC the more anonymous cells with an intermediate appearance, showing both some TC and some HC characteristics, and as “uncertain” the cells with morphology in between PHC and TC. Clinical validation supports the assumption that TC mostly represent real TC, in addition to a proportion of the PHC, while HC are false positive hematopoietic cells [30]. This does not exclude the possibility of false positive HC classified as TC or vice versa. For the present study, nuclear staining was omitted and a BCIP/NBT blue substrate solution was used for visualization of antibody binding to prevent interference with the subsequent genomic analysis steps. This yields a less complete rendering of cytological details than the standard hematoxylin nuclear staining and the red New Fuchsin solution, a fact that may have increased the chance of misclassifications of the identified cells [29, 30].

Following microscopic identification of the desired cells, the single cells were isolated using a CellTram Vario Microinjector and Eppendorf Transferman NK 2 micromanipulation equipment (Eppendorf, Hamburg, Germany) combined with an inverted Axiovert 40 C microscope (Carl Zeiss MicroImaging, Jena, Germany).

### Whole genome amplification

Following isolation of the selected cells from the cytospins, the single cells were lysed and their DNA amplified using the GenomePlex® Single-Cell Whole-Genome Amplification Kit (Sigma-Aldrich, St. Louis, MO, USA) but using the Titanium Taq DNA polymerase (BD Biosciences Clontech, Heidelberg, Germany) instead of the kit’s standard polymerase, as described by Mathiesen et al. [26].

### Library preparation and sequencing of DTCs and primary tumors with matched blood

The first steps of the whole genome- and exome-sequencing library preparation for the single cells and primary tumor with matched normal, respectively, were similar. DNA (1 μg) was sheared using the S220 Focused-ultrasonicator (Covaris, Woburn, MA, USA). The sample was column cleaned using a Qiaquick PCR purification Kit (Qiagen, Valencia, CA, USA) and eluted in 36 μl elution buffer (EB), followed by fragment end repair using the End-It™ DNA End-Repair Kit (Epicenter, Madison, WI, USA). The end repair master mix (MM) was made as specified by the manufacturer and 16 μl mixed with 34 μl sheared DNA and incubated at room temperature for 45 minutes. Subsequently, each sample underwent column cleaning and was eluted in 34 μl EB followed by A-base addition using an A-addition master mix and incubated at 37 °C for 30 minutes. The A-addition master mix consisted of 5 μl 10× NEBuffer 2 (New England Biolabs, Ipswich, MA, USA), 10 μl dATP (1 mM; Sigma Aldrich, MO, USA), and 3 μl Klenow [3′- > 5′ exo-] (Thermo Scientific). Following A-base addition, samples were column cleaned and eluted in 40 μl EB. Adaptors were ligated onto the DNA fragments using an adaptor ligation master mix and each sample was incubated at 16 °C for 20 minutes. The adaptor ligation master mix was made out of 5 μl 10× T4 Ligase Buffer (New England Biolabs, Ipswich, MA, USA), 5 μl adaptor (50 μM), and 2 μl T4 DNA Ligase (2000 U/μl) (New England Biolabs, Ipswich, MA, USA) for each sample. Samples were column cleaned and exome- and whole genome libraries were eluted in 20 μl and 50 μl EB, respectively. Size selection (150–350 bp) of exome library fragments was then performed using an E-Gel® electrophoresis system with a 2% agarose gel (Thermo Fisher Scientific, MA, USA). Exome sequencing libraries were bead cleaned using Agencourt AMPure XP (Beckman Coulter, CA, USA) and eluted in 50 μl EB. Subsequently, both the exome- and whole genome-sequencing libraries were PCR-amplified; the whole genome libraries have been described in detail previously [40]. Exome libraries were amplified using an amplification master mix consisting of 1.5 μl universal primer, 1.5 μl index primer, 125 μl 2× iProof High-Fidelity Master Mix (Bio-Rad Laboratories, Hercules, CA, USA), and 72.5 μl MQ water. The MM was added to the template and spread across five wells at a reaction volume of 50 μl and run for 12 cycles. After PCR amplification, quality assessment of samples was performed using a Bioanalyzer (Agilent Technologies, Santa Clara, CA, USA). Exome capture was performed using NimbleGen capture hybridization beads (Roche, Basel, Switzerland). For each sample, 100 μl of blocking primer master mix was added to a tube of 5 μl dried Cot-1-DNA (Invitrogen, Carlsbad, CA, USA). The MM was prepared by adding 10 μl of each blocking primer: B01.P5.F, B02.P5.R, B01.P5.F, B03.P7.part1.F, B04.P7.part1.R, B05.P7.part2.F, and B06.P7.part2.R (100 μM). Blocking primer MM (60 μl) and template DNA (1 μg) were added to the dried Cot-1-DNA, resuspended, and dried by speed-vacuum at 45 °C. The hybridization MM was prepared by adding 7.5 μl 2× Hybridization Buffer and 3 μl Hybridization component A and added to the template and resuspended. The mixture was denatured at 95 °C for 10 minutes. Capture probes (4.5 μl) were added to the mixture and samples were incubated at 47 °C for 64–72 h. Post-capture, the samples were washed using Streptavidin Dynabeads (Termo Fisher Scientific, MA, USA) following the manufacturer’s protocol. Subsequently, post-capture PCR was performed for ten cycles. Exome- and whole-genome libraries were sequenced on HiSeq 2000 (Illumina, San Diego, CA, USA).

For single cells 003G–L, 017E–I, 019B–D, 044G–H, 083G–I and 107R–U, 0.5 to 1 ng of amplified gDNA was used for library preparation using the Nextera XT kit (Illumina) following the manufacturer’s instructions. The only deviation from the original protocol was that reaction volumes were scaled down by a factor of 2. Samples were barcoded and multiplex sequenced, Rapid Run Mode, on a HiSeq2500 (Illumina).

### Copy number analysis of primary tumors

DNA from the primary tumors or lymph node metastasis was analyzed for CNAs using the Genome-Wide Human SNP array 6.0 platform (Affymetrix, Santa Clara, CA, USA). The data were normalized to the HapMap samples using Affymetrix Power Tools. The Battenberg algorithm [4], an extension of the Allele-Specific Copy number Analysis of Tumors (ASCAT) algorithm [41], was used to estimate the aberrant cell fraction and tumor ploidy and call (sub)clonal CNAs. Briefly, Battenberg employs Impute2 [42] with the 1000 Genomes haplotypes as a reference panel to phase heterozygous SNPs [43]. Phased SNP B-allele frequency (BAF) values are then segmented using piecewise constant fitting (PCF) [44], resulting in segmented BAF values for each segment. Each identified chromosomal segment is then checked for deviations from clonality using a standard *t*-test comparing the fitted BAF value to that of a clonal solution. Clonal copy number and Battenberg (sub)clonal copy number analysis results are included in Additional file 3.

### Copy number analysis of DTCs

Copy number analysis of the DTCs was performed as previously described [40]. The single-cell sequencing reads were trimmed for putative WGA adapter sequences and aligned to the GRCh37 human reference using Burrows-Wheeler Aligner (BWA) [45]. LogR values were calculated for genomic bins of 500,000 uniquely mappable positions, corrected for GC bias, and segmented using PCF (the penalty parameter, γ, was set to 25) [44]. Copy number was estimated per segment as 2^logR^ × Ψ, where Ψ is the ploidy of the cell (Additional file 3). The BAF was calculated for each SNP position from dbSNP (dbSNP build 135). To measure noise in the single-cell read-depth profiles, we determined the median absolute pairwise distance (MAPD) value, which is a measure of the median distance between two consecutive GC-corrected logR data points genome-wide.

### Power analysis

The retrospective power analysis was performed in analogy to rarefaction analysis in ecology. The cumulative number of unique CNAs was plotted as a function of the cumulative number of single cells, resulting in a saturation curve. The order of the cells was randomized 100,000 times, and mean and standard deviations were calculated. If the curve flattens off, a reasonable number of cells have been sequenced and including more cells is likely to yield only few additional CNAs/clones. To decrease the influence of noise, CNAs <5 Mb were excluded. Unique CNAs were defined as those of which at least one breakpoint was >5 Mb away from those of another, overlapping CNA and it had a different total copy number state.

### Mutation calling on bulk tumor exomes and subsequent genotyping in single cells

From the exome sequences of bulk primary tumors and matched normal samples somatic mutations were called using MuTect [46] on tumor-normal pairs using only those reads with a mapping quality ≥30. The resulting somatic mutations were further filtered against germline variants present in: 1) any of the bulk normal exomes from the six patients in this study and a deep sequenced whole genome from a normal B-lymphoblastoid cell line (HCC38-BL); 2) an open source project, NGS-logistics, which provides the frequency for given loci in unrelated and unaffected individuals [47] and an in-house panel of 350 unrelated and unaffected individuals. Mutations falling within centromeres or in intergenic regions or repeats were discarded as well, as were those with an allelic fraction <15% or displaying complete strand bias. Finally, all remaining somatic mutations were visually validated using IGV [48] and likely false positives were discarded (Additional file 1: Figure S10). GATK [49] was used to genotype the primary tumor (or lymph node metastasis) somatic mutations in the single-cell sequences.

One variant called as a somatic mutation by MuTect was found present in normal cell 107B but was subsequently validated to be a germline SNP as follows. DNA from both blood and tumor from patient MicMa107 was genotyped using a TaqMan® SNP Genotyping Assay C_175074794_10 (Applied Biosystems by Thermo Fisher Scientific) in a 7900HT Fast Real-Time PCR System. The reaction was carried out in 25 μl total volume with 1× TaqMan® Genotyping Master Mix, 1× TaqMan® SNP Genotyping Assay, and 1.5 ng of DNA. This assay confirmed that both tumor and normal cells were heterozygous for this SNP.

### Genotyping germline SNPs in single cells and construction of error models

To assess the power of our approach to detect single nucleotide variants in the single cells, we checked the distribution of germline heterozygous SNPs in all the patients and respective cells. In contrast to the somatic substitutions, which are exclusively detected in the DTCs, the germline SNPs should be largely uniformly distributed over all single cells, irrespective of their classification. We hence constructed a gold standard set of heterozygous germline SNPs based on those SNPs that had a B-allele frequency between 0.3 and 0.7 in the SNP-array data of the matched blood sample and a heterozygous genotype in both blood and tumor exomes (minimum coverage of 10×). We similarly constructed a gold standard set of homozygous germline SNPs, selecting those that had a B-allele frequency ≤0.1 or ≥0.9 in the matched blood SNP array data, were homozygous in both blood and tumor exomes (minimum coverage of 10×), and were located in an area with normal 1 + 1 copy number (or 2 + 2). To genotype the SNPs in the exomes and the single-cell sequences, GATK [49] was employed using UnifiedGenotyper with the --EMIT_ALL_SITES option.

We further leveraged the resulting SNP data to obtain estimates of the (per cell) false positive and false negative rates of our approach. The former we define as the detection in a single cell of the alternative allele for a homozygous reference SNP and is likely due to sequencing or WGA errors, e.g., at position “x” the genotype is AA (homozygous reference) as observed in the bulk, yet we find at least one read containing a T (the alternative allele) in a single cell. Instead, the false negative rate considers the failure to detect the alternative allele of a heterozygous SNP due to low coverage or WGA-induced allelic dropout; e.g., at position “y”, the genotype is AT (heterozygous) as observed in the bulk, yet we only find reads reporting A (the reference allele) in the single cells. The locus dropout rate was computed as the fraction of gold standard heterozygous SNPs demonstrating no single-cell sequence coverage.

The estimates can be used to model the observed patterns of detected reference and somatic variant alleles as deriving from a binomial distribution *B(n,p)* where *n* is the number of loci with coverage (Bernoulli trials) for that single cell and *p* is the false positive or false negative error rate (*p*_*fp*_ and *p*_*fn*_). However, to take the uncertainty on the parameter estimates into account (we have *p̂* and *var(p̂)* instead of p), we adopt a beta-binomial model in which the binomial probability of success *p* is not fixed but follows a beta distribution *Beta(a, b)* such that *μ* = *p̂* = *a*/(*a* + *b*) and *σ* ^*2*^ *= var(p̂)* = *ab*/((*a* + *b*)^2^(*a* + *b* + 1)). We then compute the probability of observing at least equally extreme patterns of somatic reference and variant detections given *n* under the false positive and false negative models. In case no germline SNP loci in a cell have a false positive or false negative genotype call, we employ a worst-case estimate of *p*_*fp*_ and *p*_*fn*_ by adding, respectively, one false positive or negative pseudocall.

### Reconstructing DTC origins and tumor evolution

Phylogenetic trees were constructed using the clonal and subclonal aberrations called by Battenberg on the tumor bulk SNP-array data. Nodes in the trees correspond to detected (sub)clones and are placed according to their estimated cancer cell fraction (CCF; the fraction of cancer cells carrying the specific aberration) so as to satisfy the pigeonhole principle, as previously described [4]. Applied to CCFs, the pigeonhole principle dictates that if two CNAs have a CCF >0.5, then there must exist at least a subset of cancer cells that contains both aberrations. Therefore, the smaller subclone (lower CCF) must be nested in the larger (higher CCF) and both are collinear on the phylogenetic tree. Including the DTCs in tree construction allowed for extensive refinement by revealing shared and mutually exclusive CNAs, resulting in linear (e.g., patient MicMa083) and branching (e.g., patient MicMa107) trees.

## Additional files

Additional file 1: Supplementary Figures S1–S10. (PDF 23 mb)
Additional file 2: Supplementary Tables S1–S7. (XLSX 99 kb)
Additional file 3: Copy number calls for all single cells and bulk samples. (ZIP 631 kb)
