# Supplementary material for: Tracing the origin of disseminated tumor cells in breast cancer using single-cell sequencing
Source: Genome Biol. 2016 Dec 9;17:250. doi: 10.1186/s13059-016-1109-7 (PMC5146893; doi:10.1186/s13059-016-1109-7)

Figure S1: Isolation of disseminated tumor cells from bone marrow aspirates. Single cells isolated from patient MicMa003 (A), MicMa017 (B), MicMa019 (C), MicMa044 (D), MicMa083 (E), MicMa107 at diagnosis (F), and MicMa107 3 years post-diagnosis (G). Each panel documents the isolation process of the indicated single cell in four subsequent images taken before picking (top left), during micromanipulation (top right), of the area where the cell was picked from after micromanipulation (bottom left), and of the isolated cell in the lid (bottom right). The immunomorphological class of each cell is provided next to its name. Boxed names indicate control cells stained with an isotype control antibody.

A

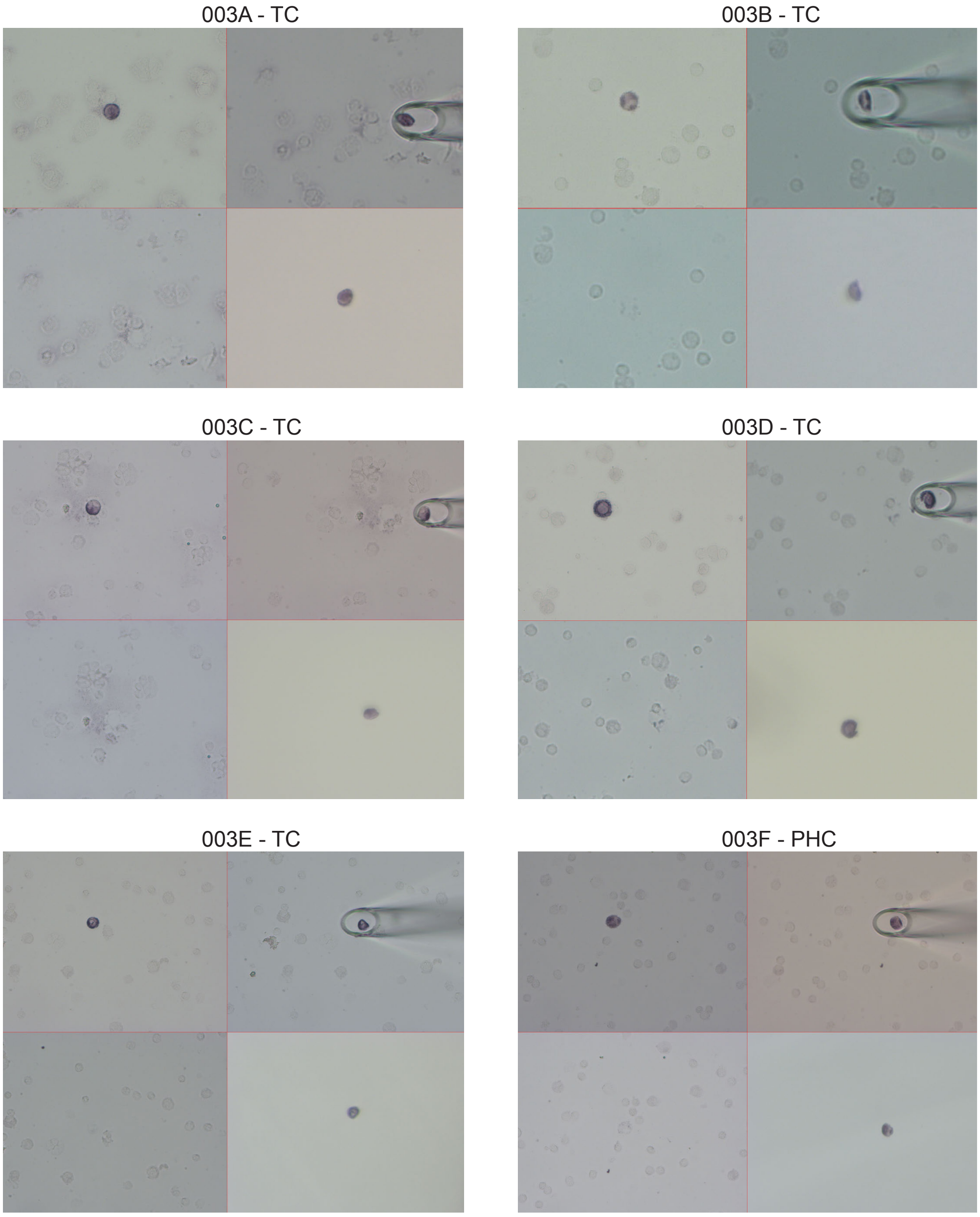

003G - PHC

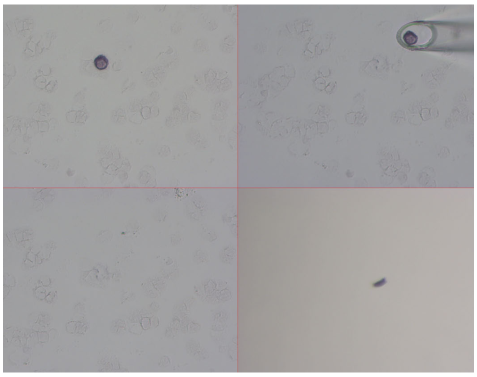

003H - HC

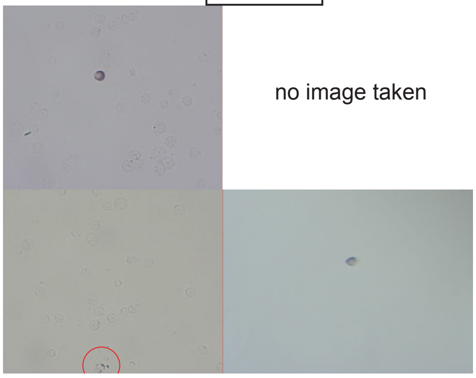

003I - HC

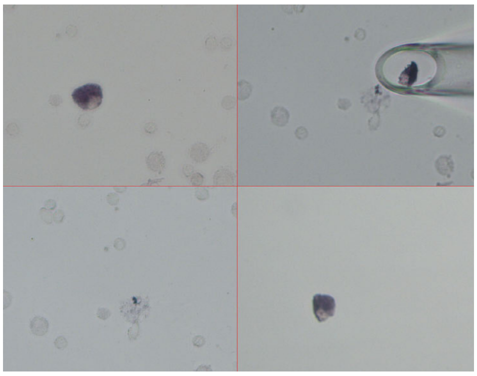

003J - uncertain

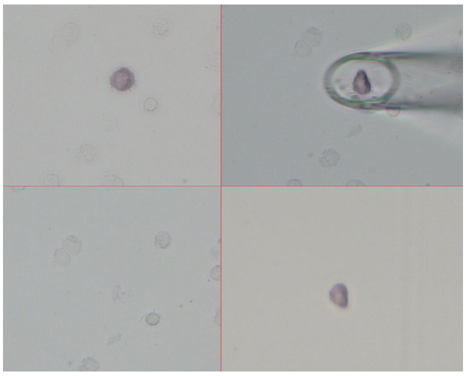

003K - uncertain

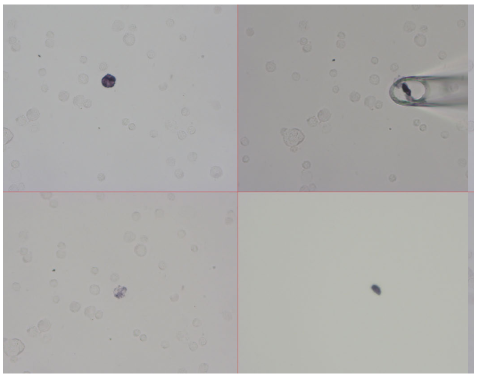

003L - PHC

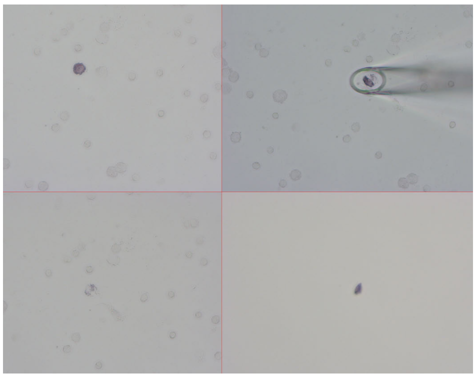

B

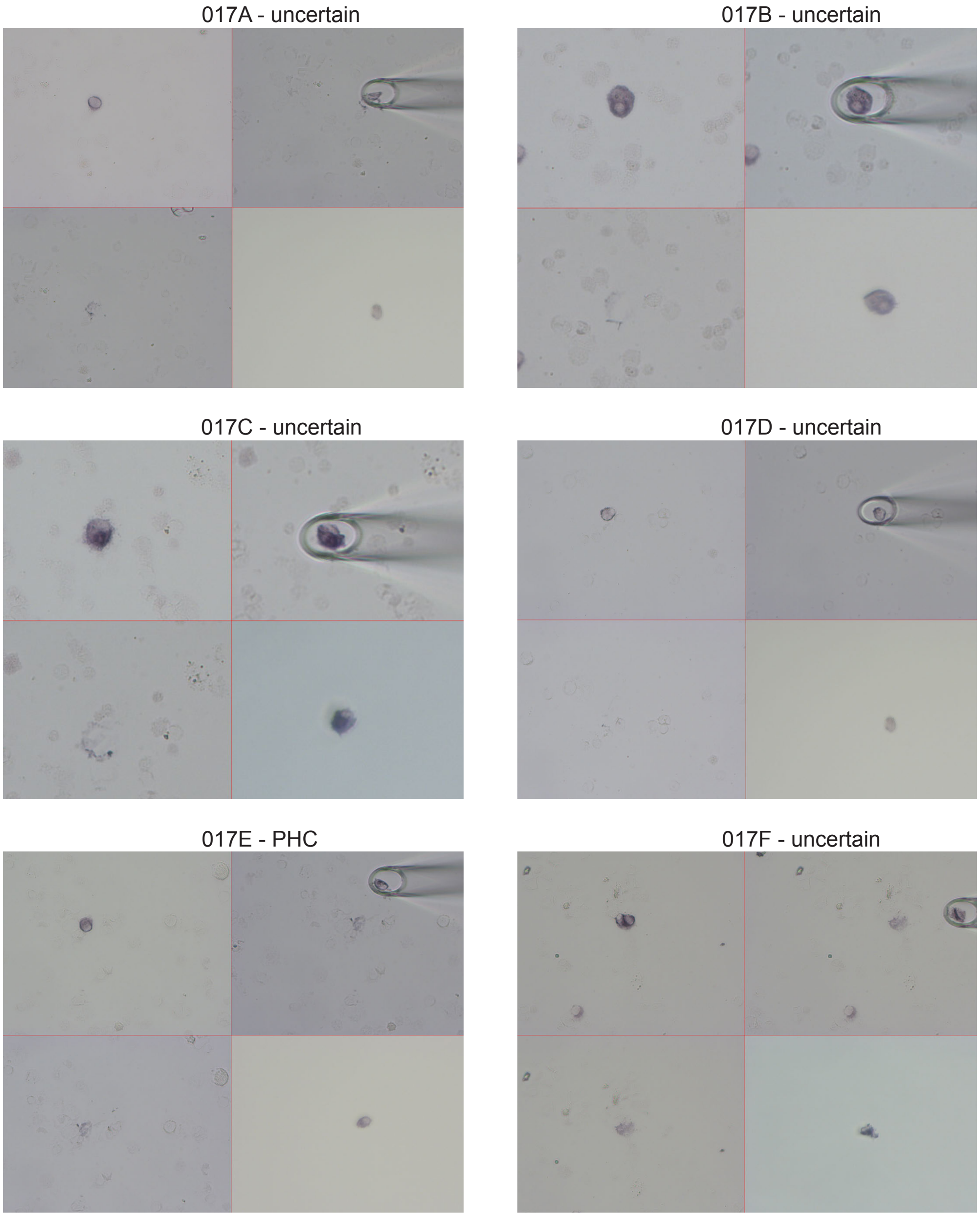
017A - uncertain
017B - uncertain
017C - uncertain
017D - uncertain
017E - PHC
017F - uncertain

017G - HC

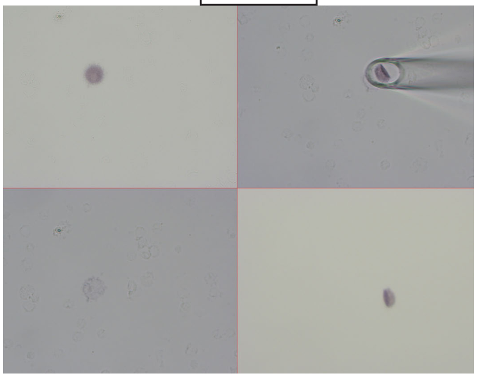

017H - PHC

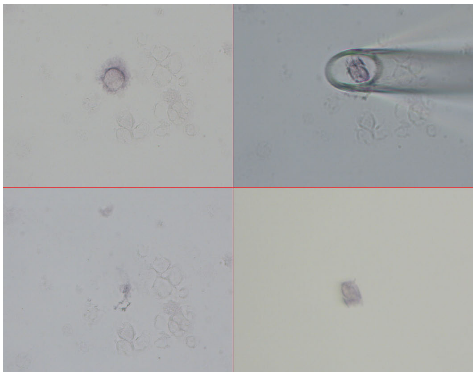

017I - PHC

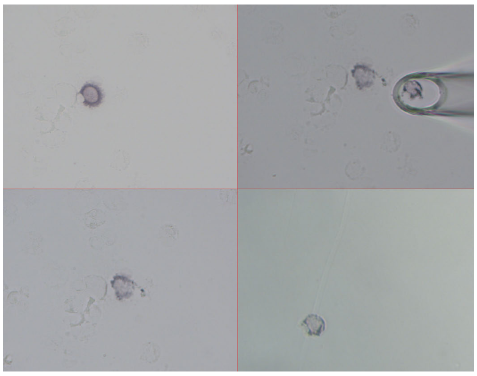

C

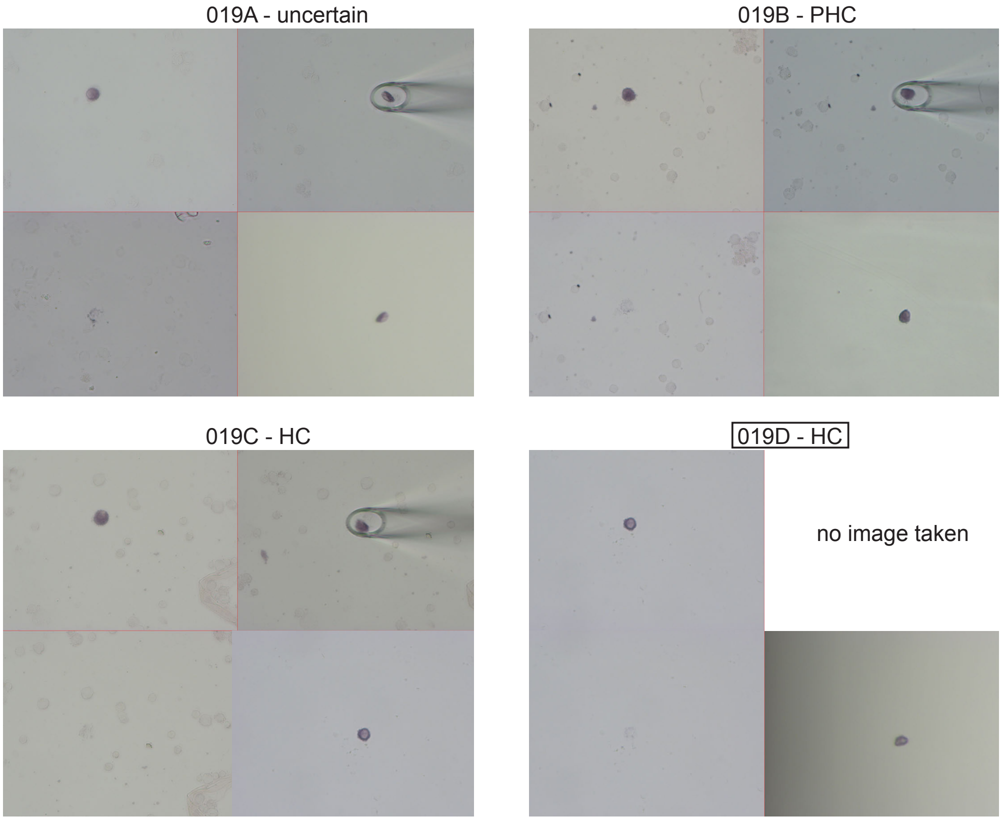

D

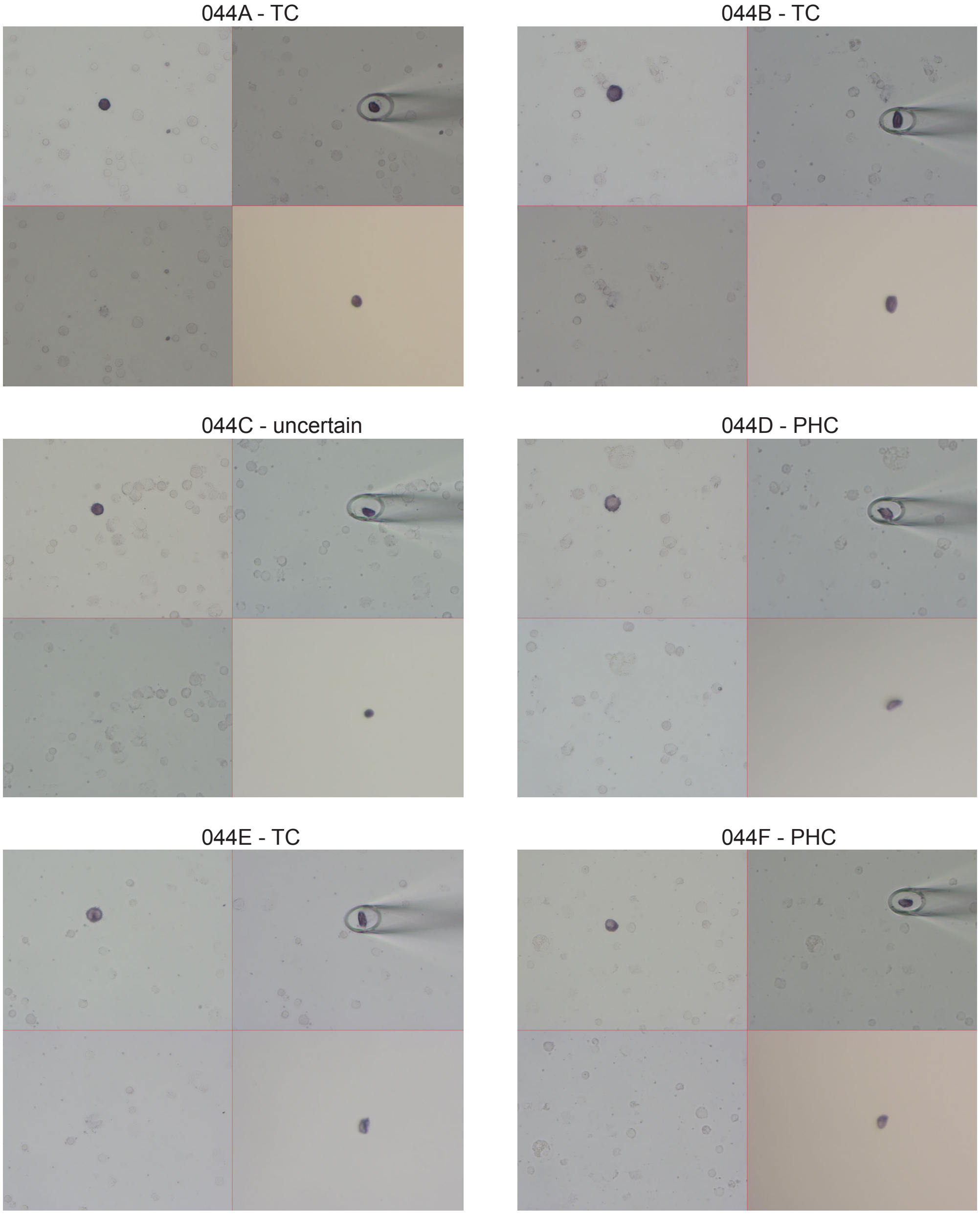

044G - uncertain

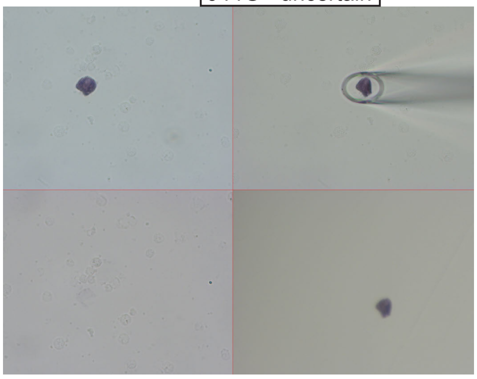

044H - PHC

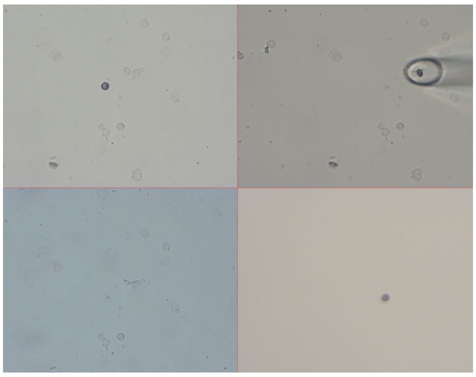

E

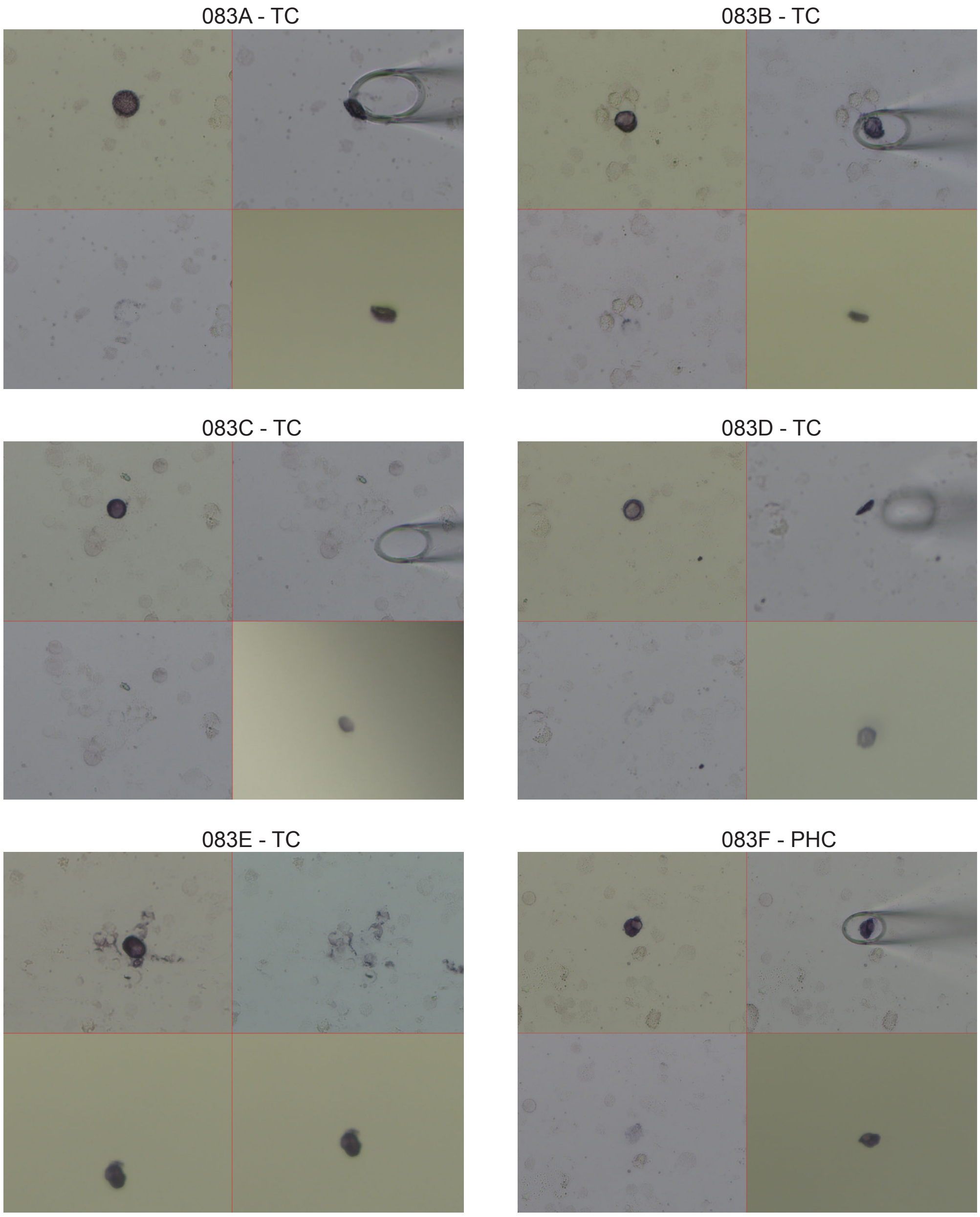

083G - TC

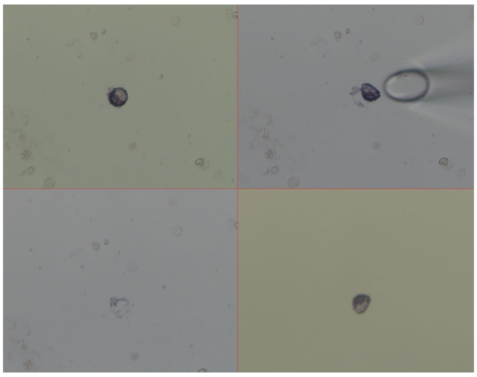

083H - TC

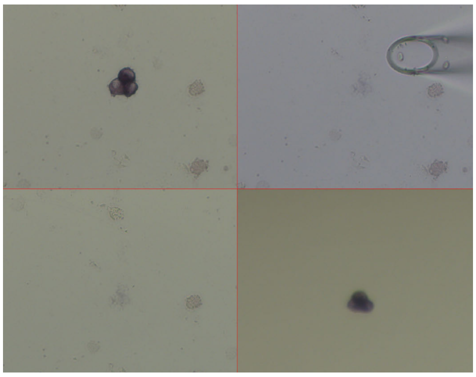

083I - HC

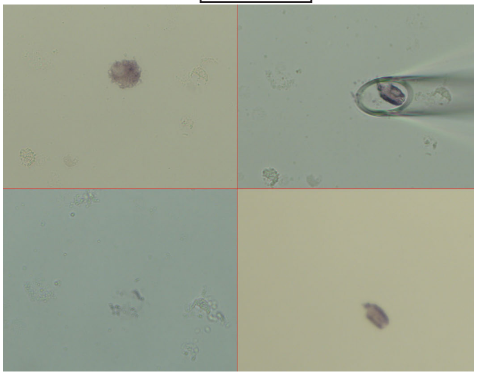

F

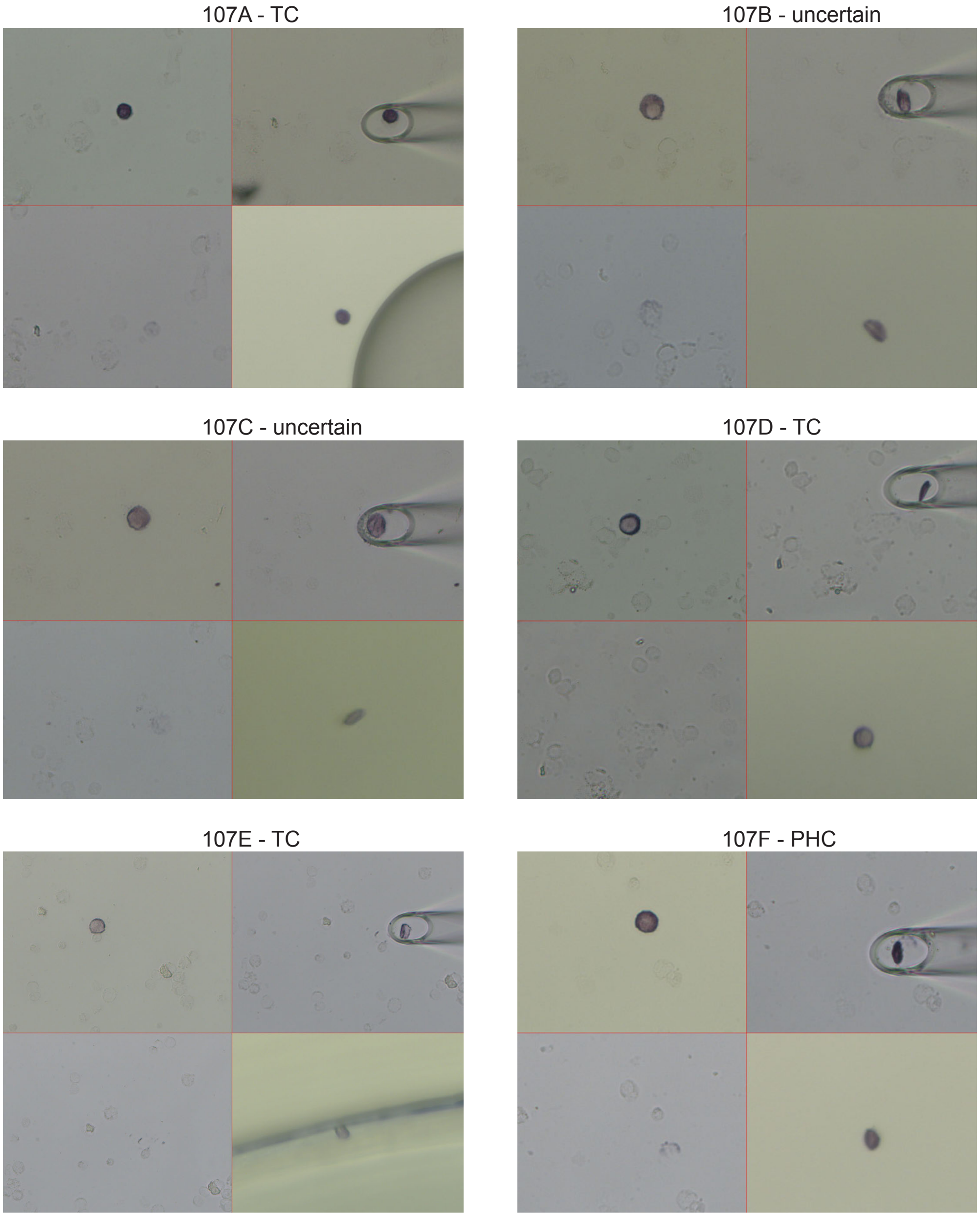
107A - TC
107B - uncertain
107C - uncertain
107D - TC
107E - TC
107F - PHC

107G - PHC

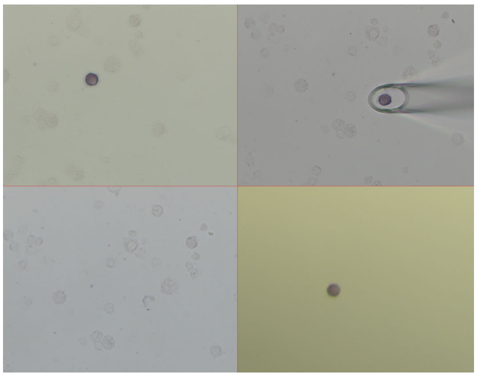

107R - PHC

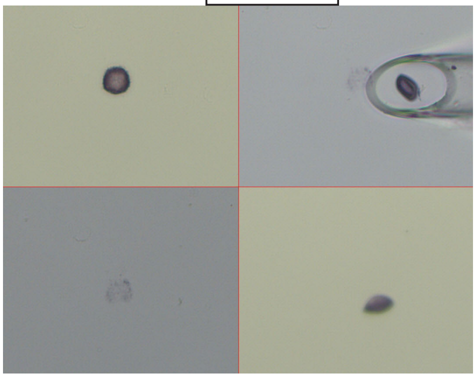

107S - uncertain

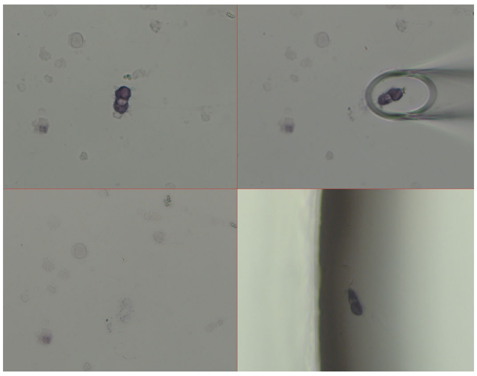

G

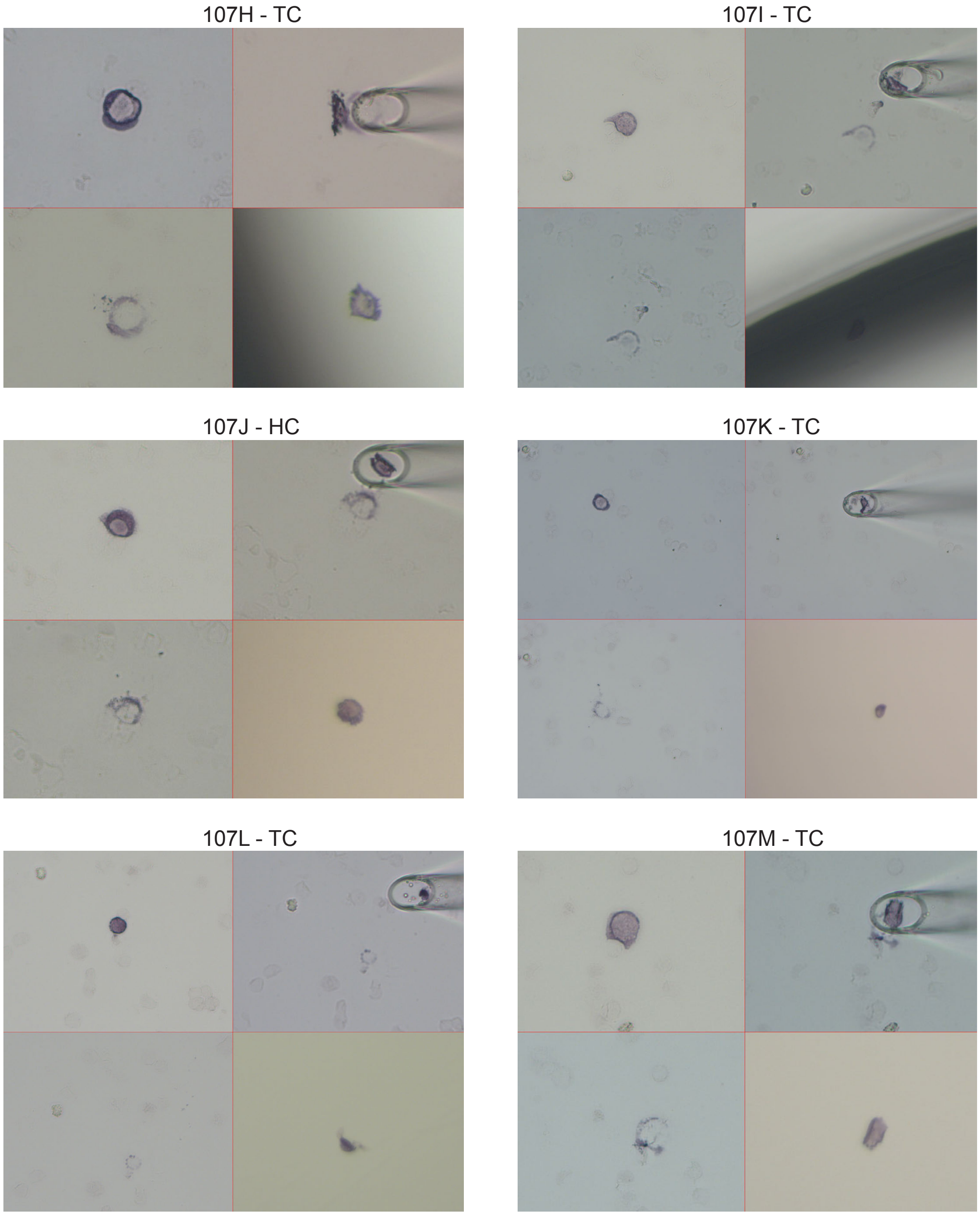

107N - TC

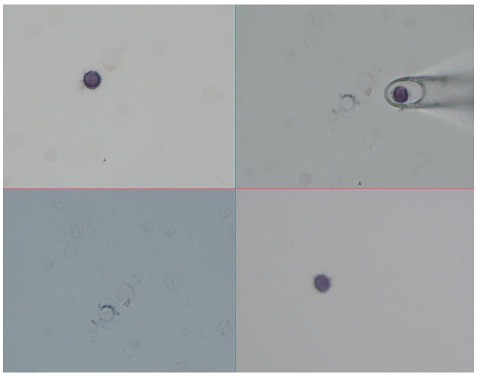

107O - TC

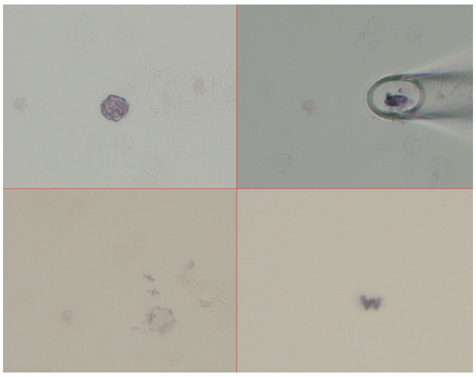

107P - TC

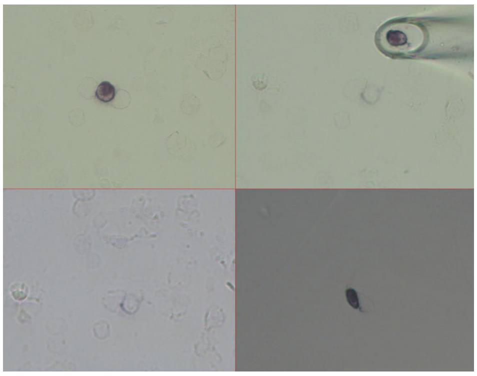

107Q - uncertain

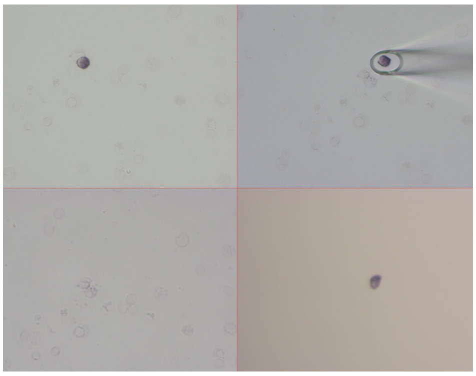

107T - HC

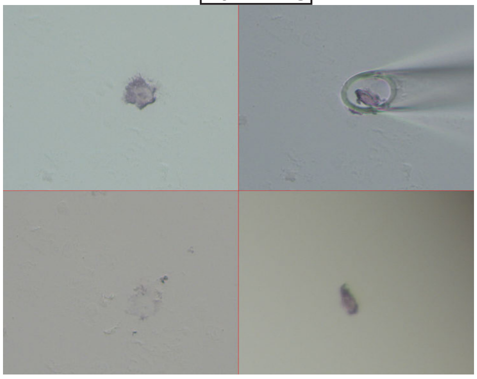

107U - TC

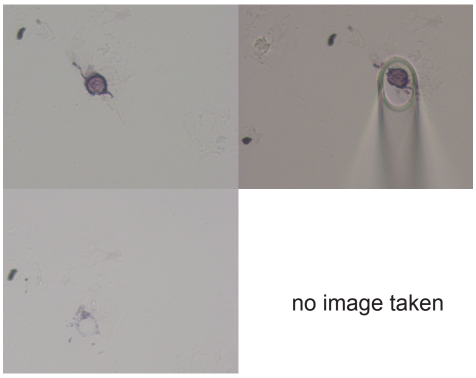

no image taken

Figure S2: Assessment of noise in single-cell logR profiles. Median Absolute Pairwise Difference (MAPD) values (Materials and methods) embody the noise in logR between two consecutive genomic bins genome wide. Red cells failed this QC filter.

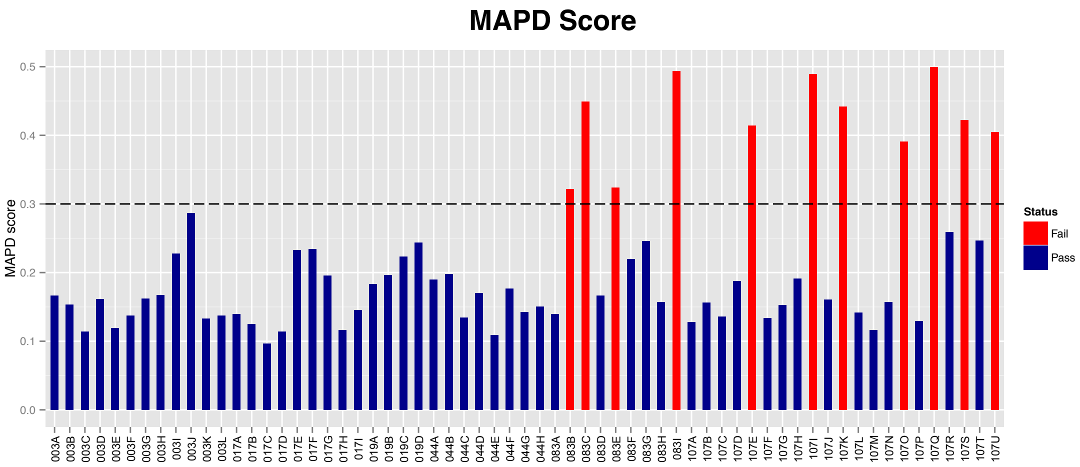

Figure S3: Focus on DNA copy number events overlapping in AUs and bulk tumor. (A-D) Per patient profiles are shown as concentric circles inside the circular human karyogram. Only those patients and regions that have overlapping CNAs between AUs and the tumor bulk are depicted. Total copy number is represented as a heat map from blue to red as indicated. Tumor samples and single cells are labeled on the corresponding circles and are color-coded: primary tumor bulk (PT) in cyan, lymph node bulk (LN) in purple, DTCs in orange, aberrant cells of unknown origin in dark green and Normal cells in green. Control cells are boxed. Panels are shown for patient MicMa017 (A), MicMa019 (B), MicMa044 (C) and MicMa107 at the time of diagnosis (D).

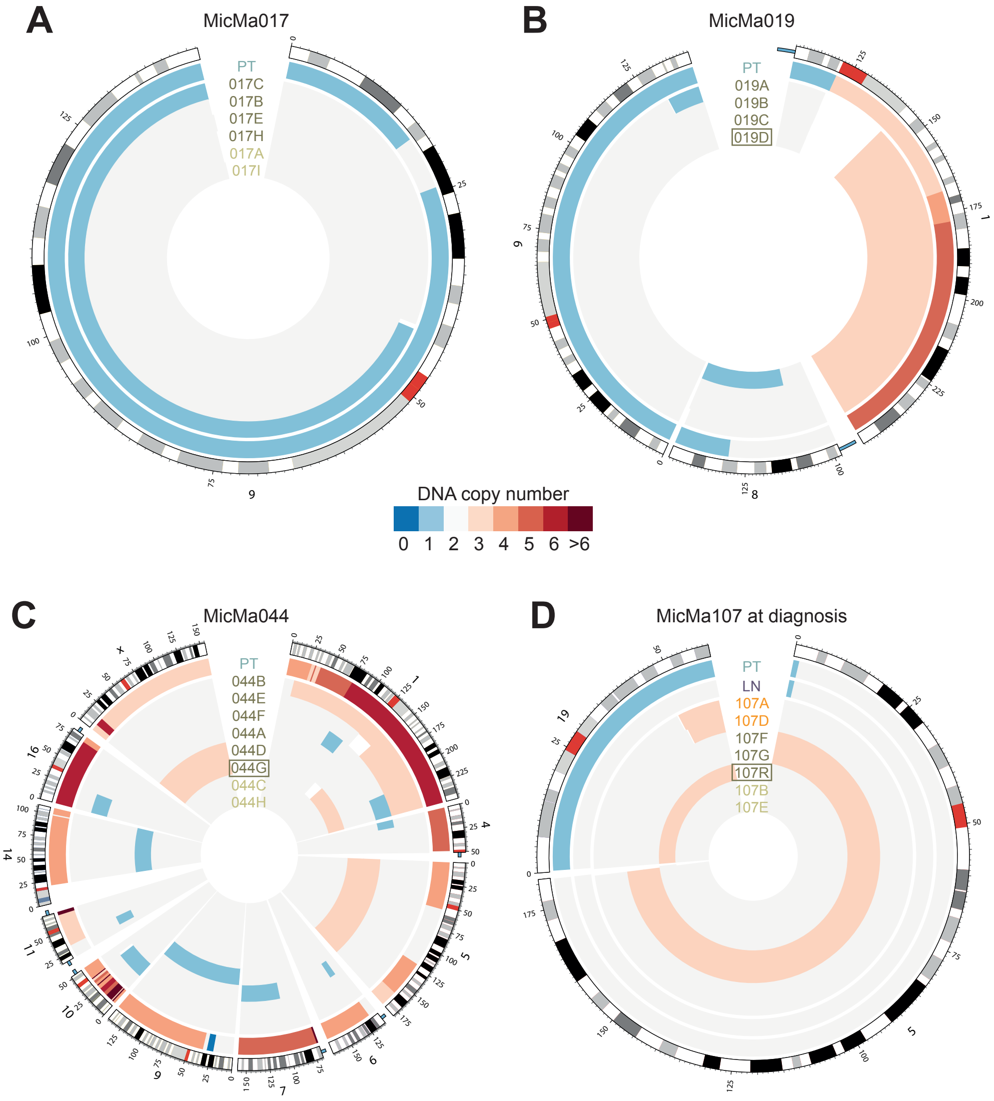

Figure S4: LogR profile of a 'Doublet' and two single cells. Genome-wide logR segments of 3 DTCs (003C, 003D, 003E) of patient MicMa003. Red and black depict logR segments obtained from single DTCs 003C and 003E, respectively, while blue indicates the logR segments of a doublet (003D).

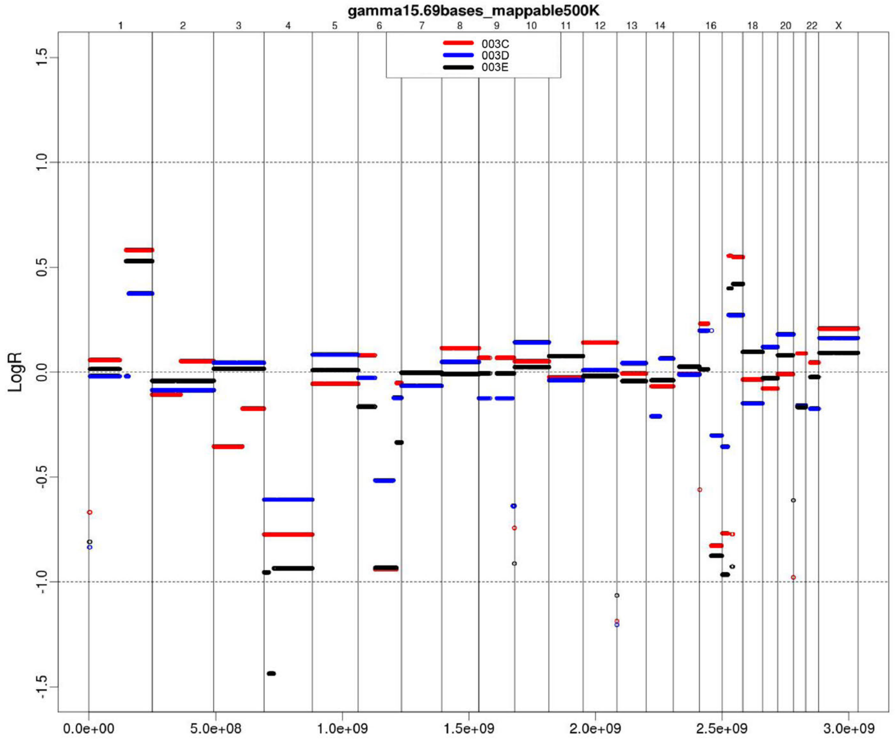

Figure S5: Retrospective power analysis. The cumulative number of unique CNAs is plotted as a function of the cumulative number of single cells for each sample. Mean and standard deviations of 100,000 randomized orders are shown. AD, at diagnosis; 3Y, 3 years after diagnosis.

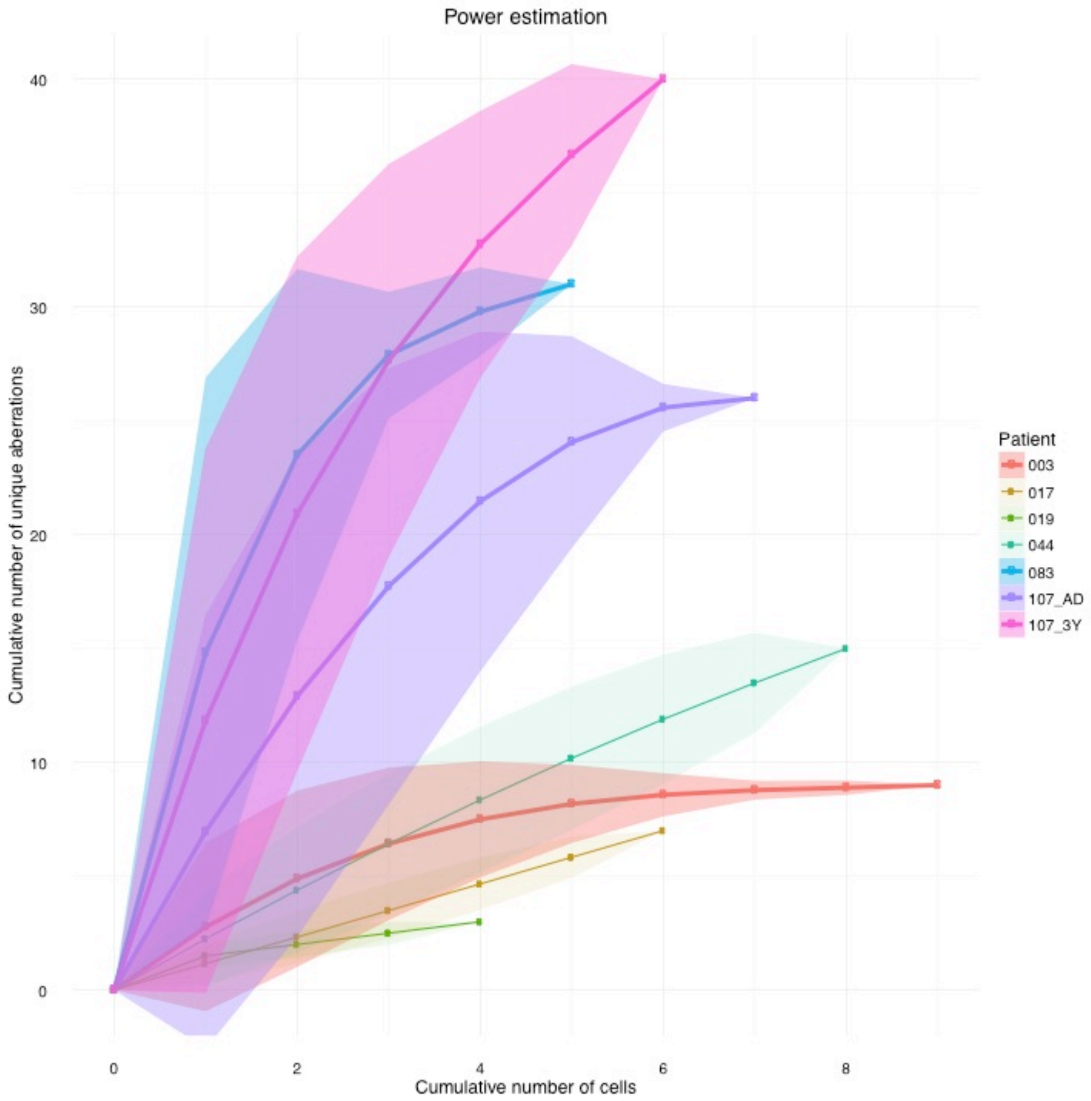

Figure S6: Coverage statistics of bulk exome sequences for all tumors and matched normal blood samples. The breadth of coverage across the exome (Y-axis) is depicted for different sequence depth cutoffs (X-axis). Primary tumor (PT), lymph node metastasis (LN) and matched blood (B).

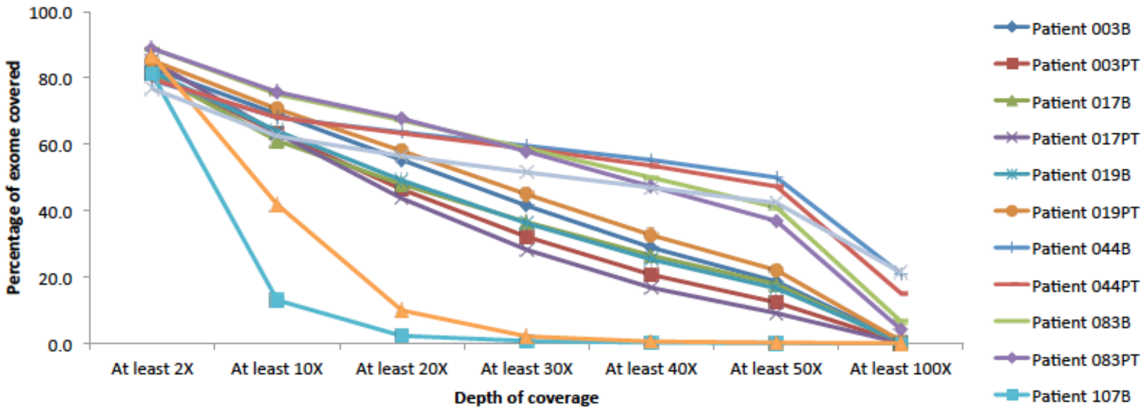

Figure S7: Genotyping of single nucleotide variants from bulk tumor exome sequences in the Doublet sequences. (A, B) Heat maps per tumor, where each row represents either a single somatic substitution called on the corresponding bulk exome and the matched blood (A), or a random heterozygous germline SNP (20 total, B), and columns represent the different Doublet or exome datasets obtained for that tumor. Tile colors reflect the presence of the mutant allele (orange), its absence (reference, blue), or whether there was no coverage at that position (white). (C) Modeled probability of observing an at least equally extreme pattern of somatic reference and variant alleles for that Doublet through false positives (none of the cells have any of the tumor's somatic mutations and hence derive from another lineage) or false negatives (at least one of the Doublet's cells derives from the tumor and hence contains these somatic mutations). Model results are encoded as heat maps of -log$_{10}$(p). For clarity, loci with zero coverage in all of the Doublets of that patient are omitted.

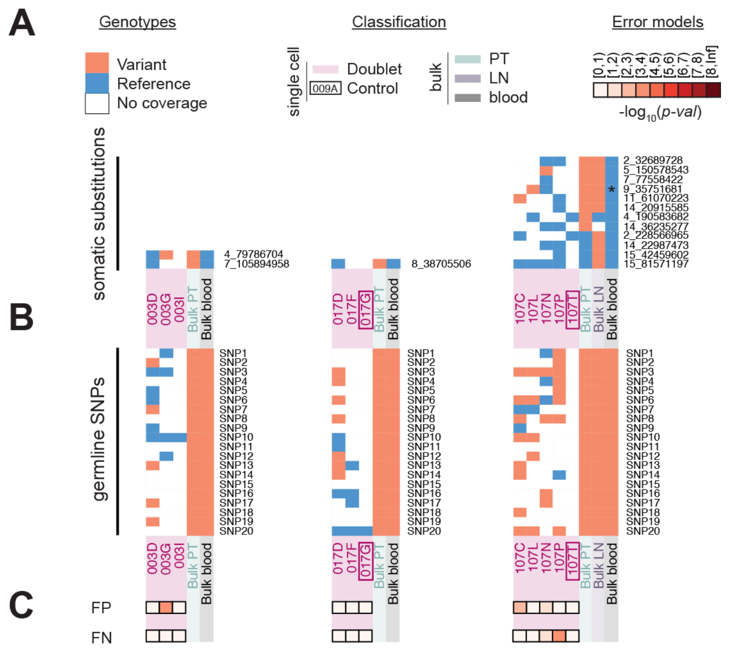

Figure S8: Tumor bulk copy number profiles and logR, copy number and BAF data of single cells from the corresponding patient. Each panel highlights the bulk copy number profile (on top) and single cell data (bottom) for a single patient/time point: patient MicMa003 (A), MicMa017 (B), MicMa019 (C), MicMa044 (D), MicMa083 (E), MicMa107 at diagnosis (F) and 3 years post-diagnosis (G). The copy number profile(s) are the output of the Battenberg algorithm based on the SNP-array data of bulk tumor DNA. Yellow indicates the total copy number, black the copy number of the minor allele. Regions with subclonal aberrations are evidenced by a non-integer copy number state. For each corresponding single cell, the logR, fitted copy number and BAF are plotted across the genome.

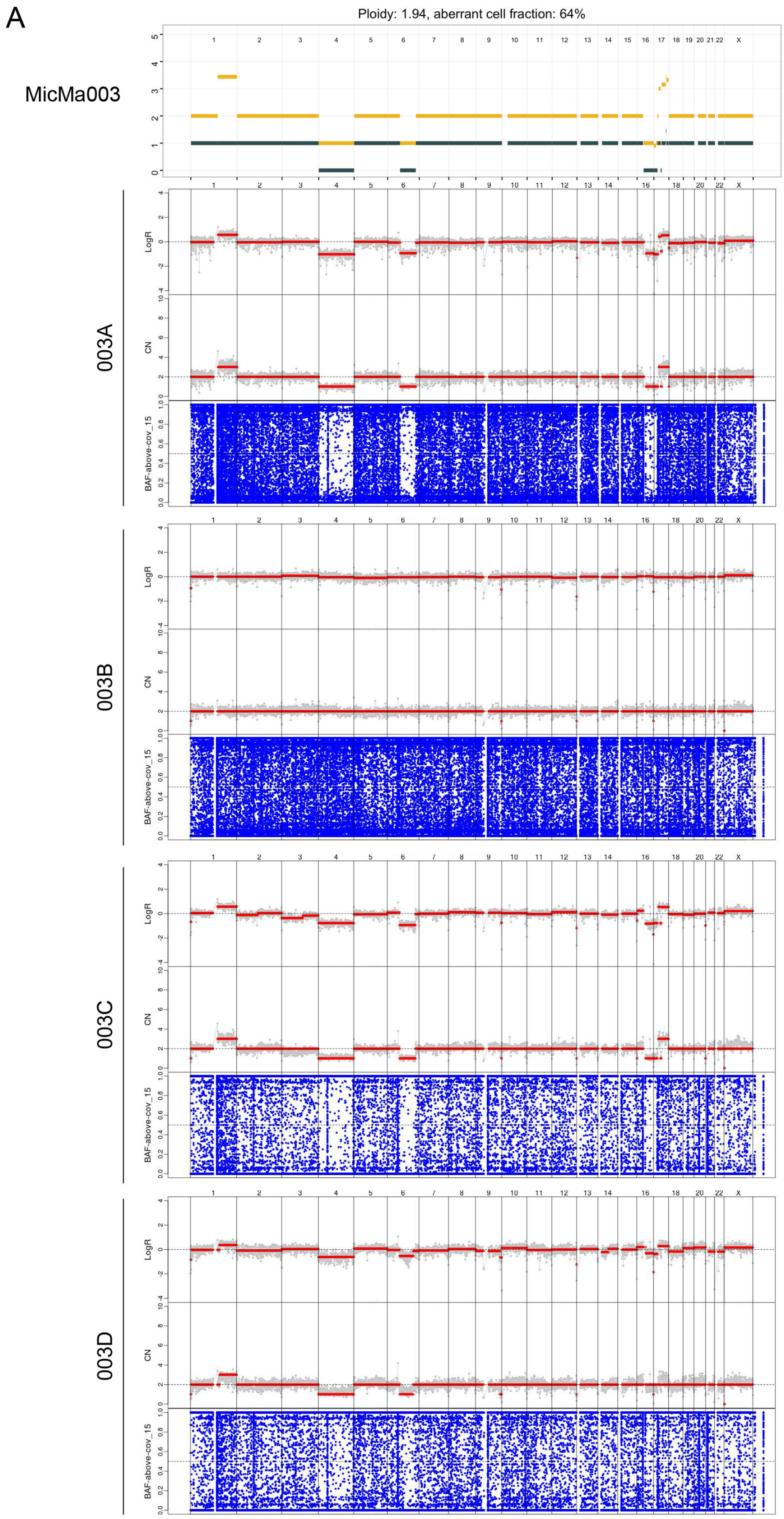
A
Ploidy: 1.94, aberrant cell fraction: 64%
MicMa003
003A
003B
003C
003D
LogR
CN
BAF-above-cov_15

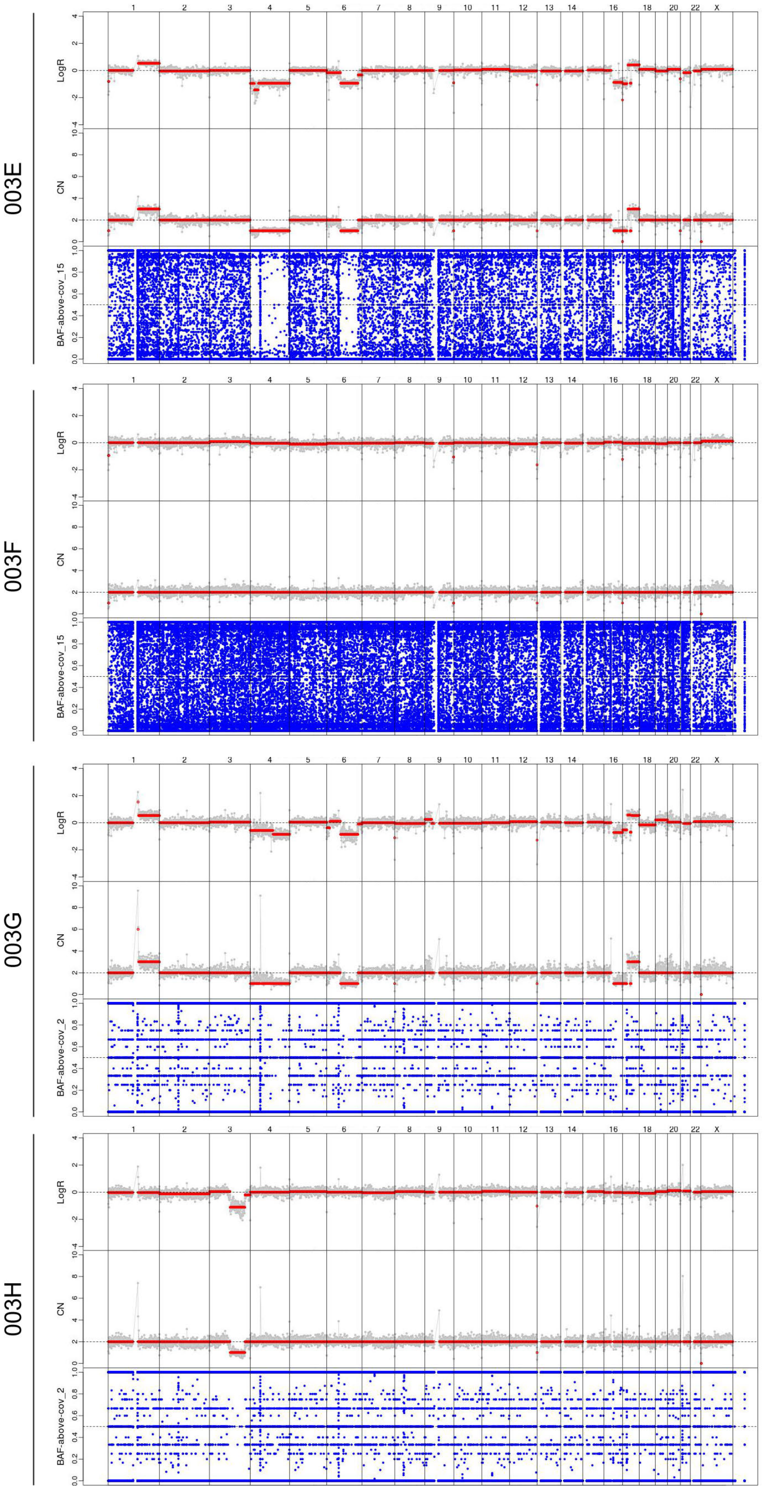
003E
003F
003G
003H
LogR
CN
BAF-above-cov_15
BAF-above-cov_2
1
2
3
4
5
6
7
8
9
10
11
12
13
14
16
18
20
22
X

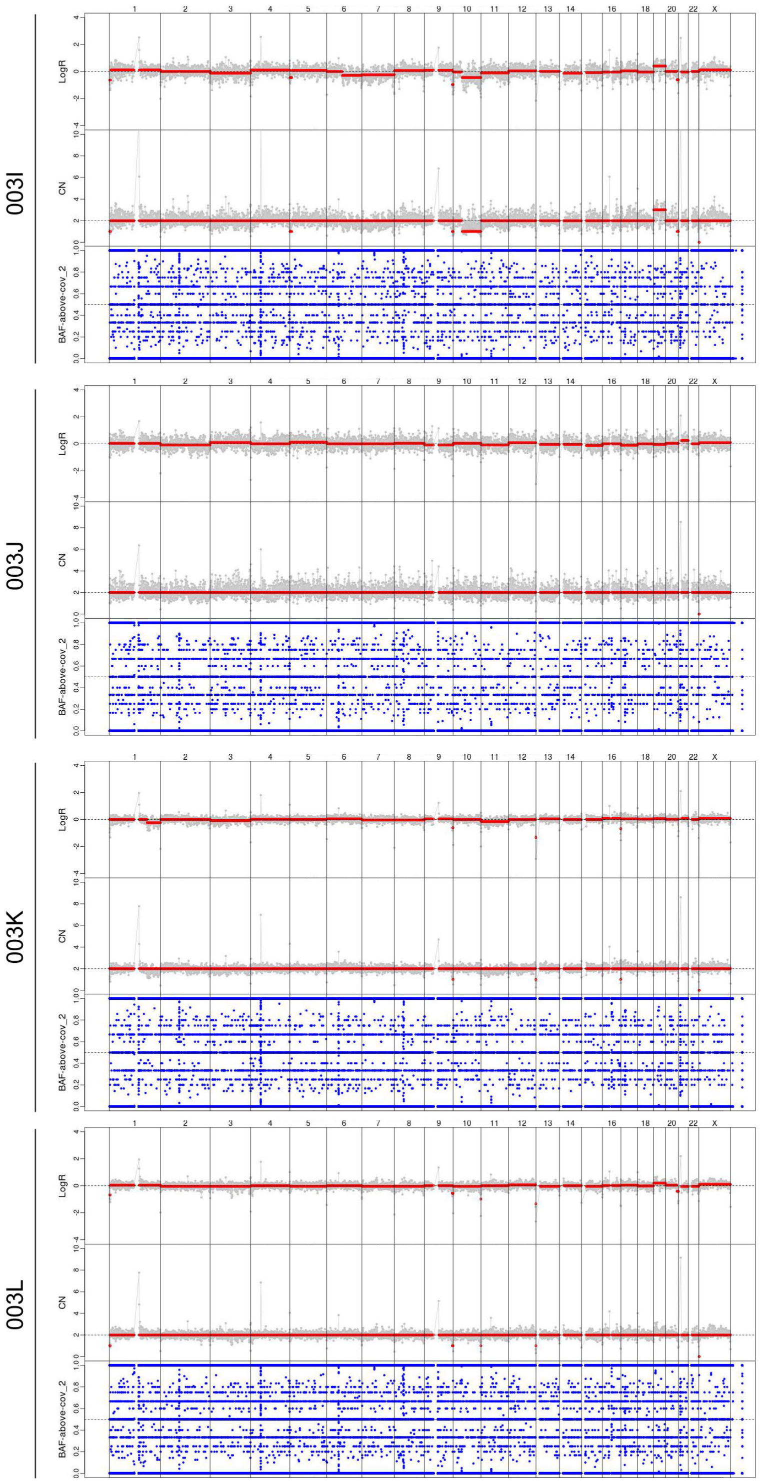

003I
003J
003K
003L
LogR
CN
BAF-above-cov_2
1 2 3 4 5 6 7 8 9 10 11 12 13 14 16 18 20 22 X

B

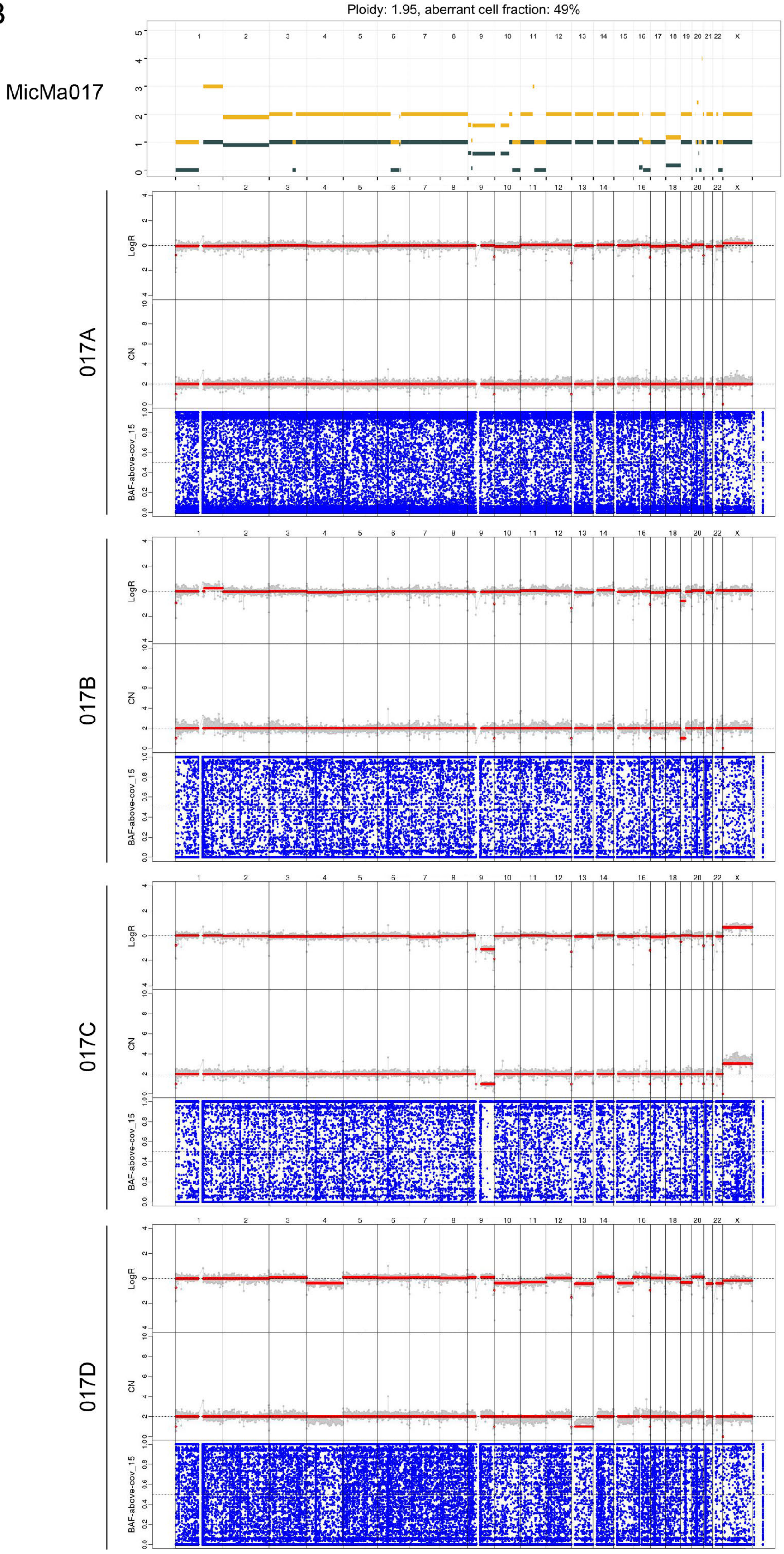
Ploidy: 1.95, aberrant cell fraction: 49%
MicMa017
017A
017B
017C
017D
LogR
CN
BAF-above-cov_15
1
2
3
4
5
6
7
8
9
10
11
12
13
14
15
16
17
18
19
20
21
22
X

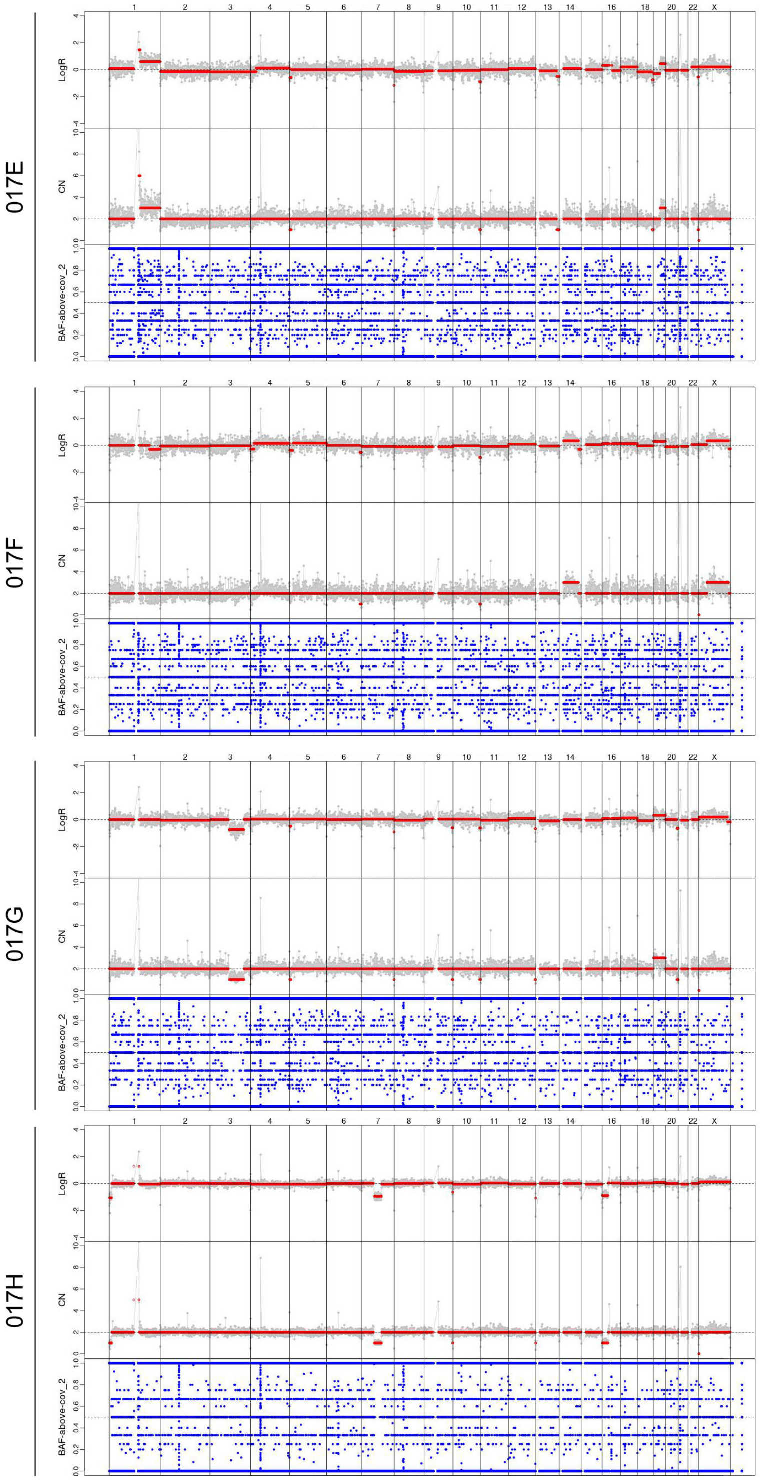
017E
017F
017G
017H
LogR
CN
BAF-above-cov_2

017I

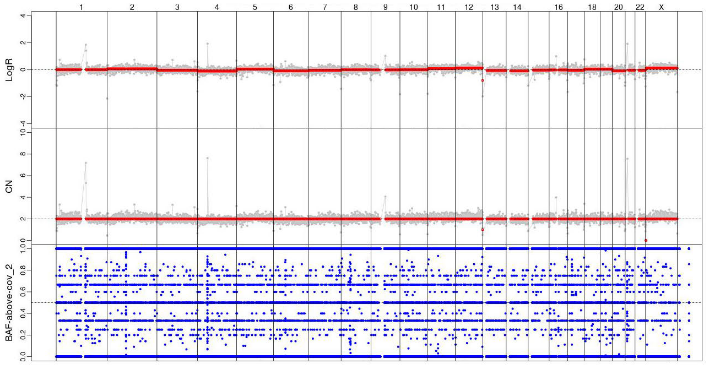

C

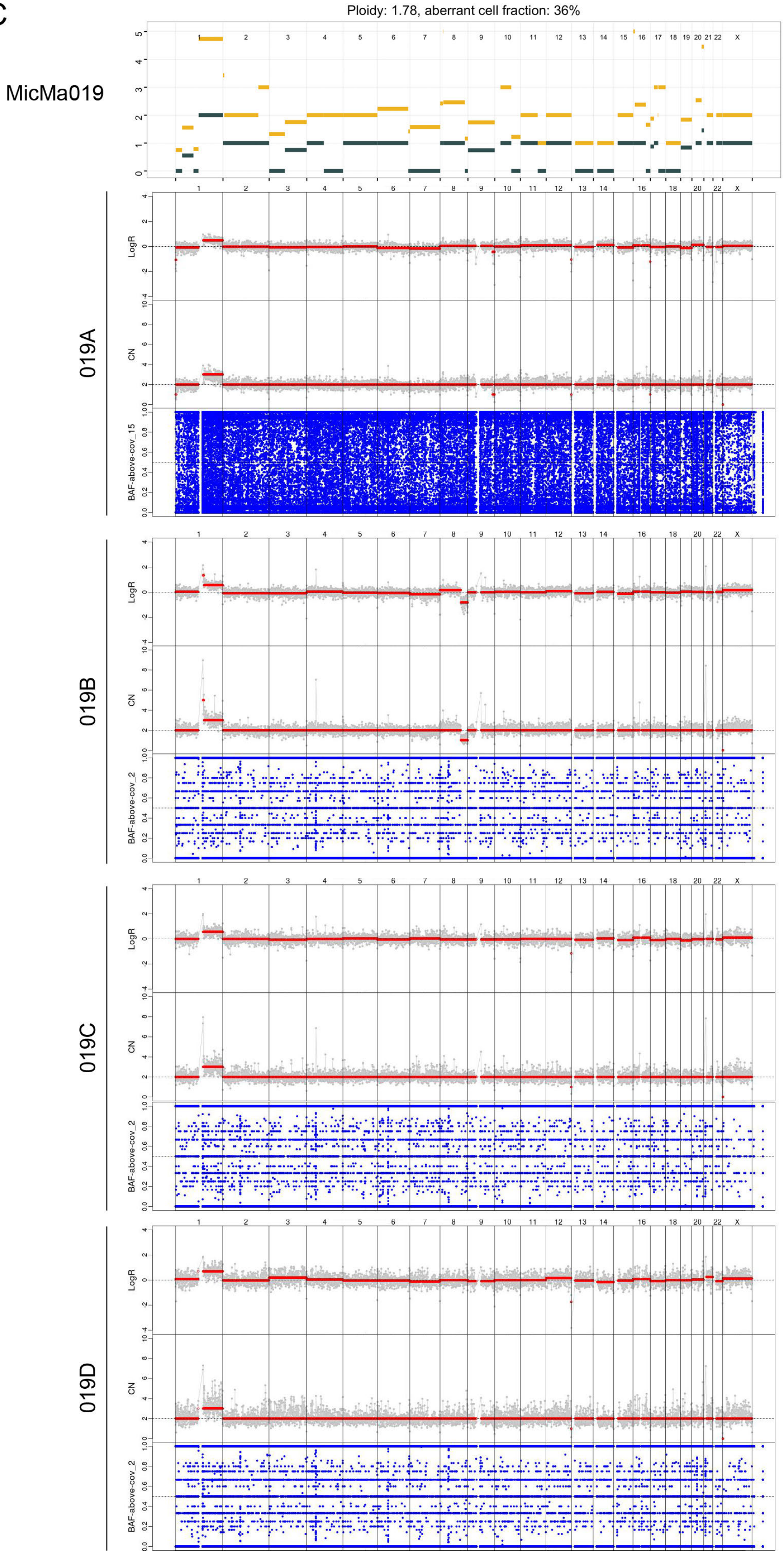
Ploidy: 1.78, aberrant cell fraction: 36%
MicMa019
019A
019B
019C
019D
LogR
CN
BAF-above-cov_15
BAF-above-cov_2

D

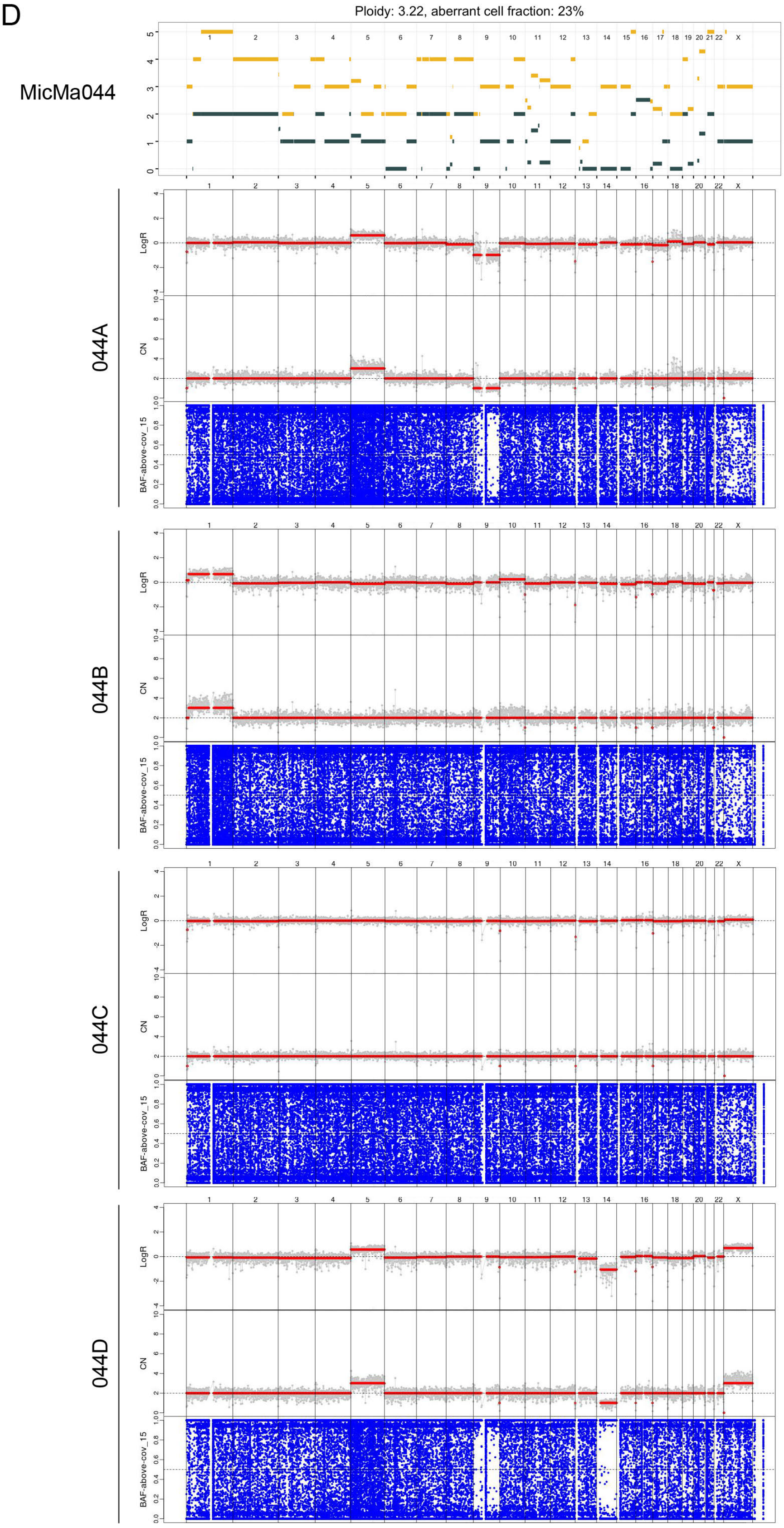

Ploidy: 3.22, aberrant cell fraction: 23%
MicMa044
044A
044B
044C
044D
LogR
CN
BAF-above-cov_15

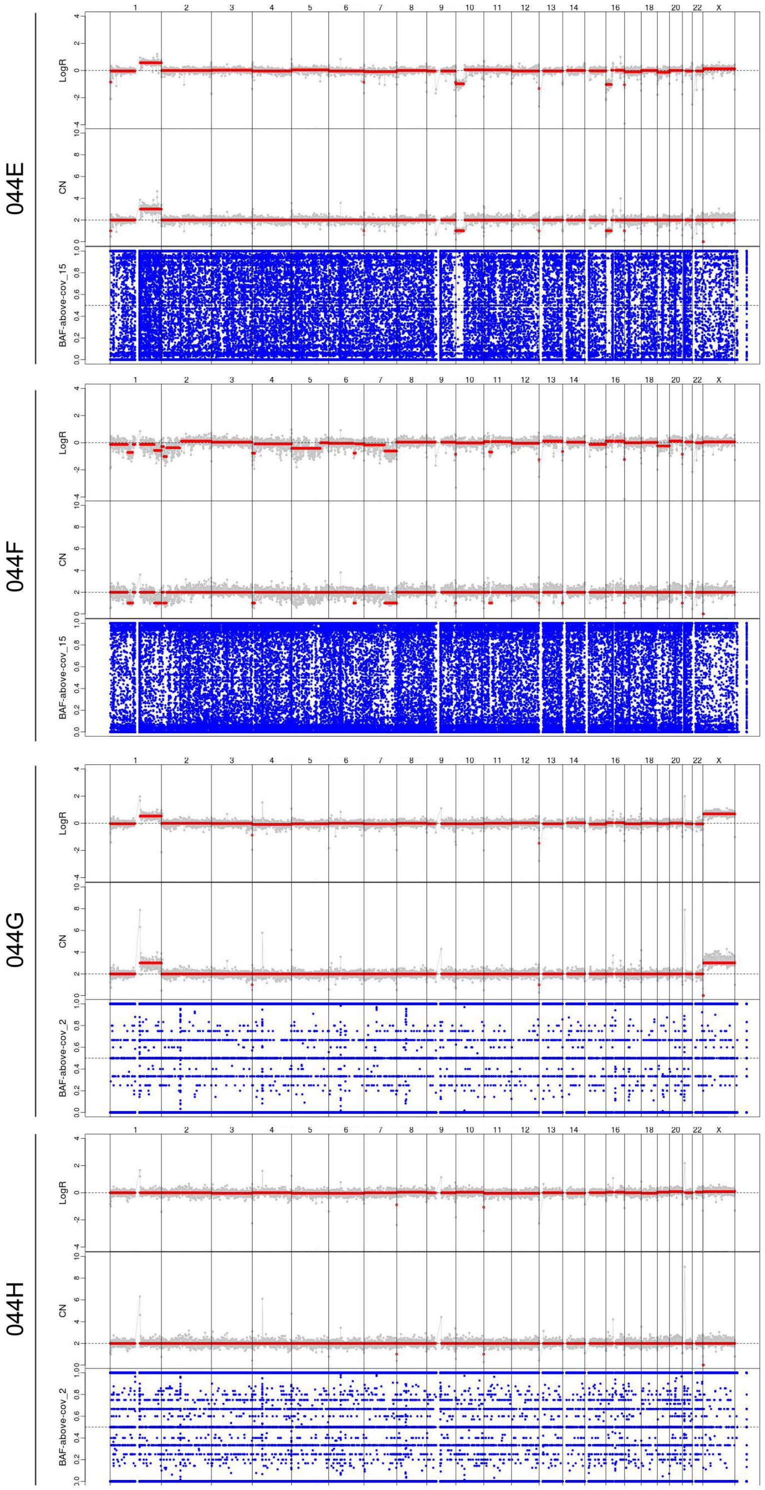
044E
044F
044G
044H
LogR
CN
BAF-above-cov_15
BAF-above-cov_2
1
2
3
4
5
6
7
8
9
10
11
12
13
14
16
18
20
22
X

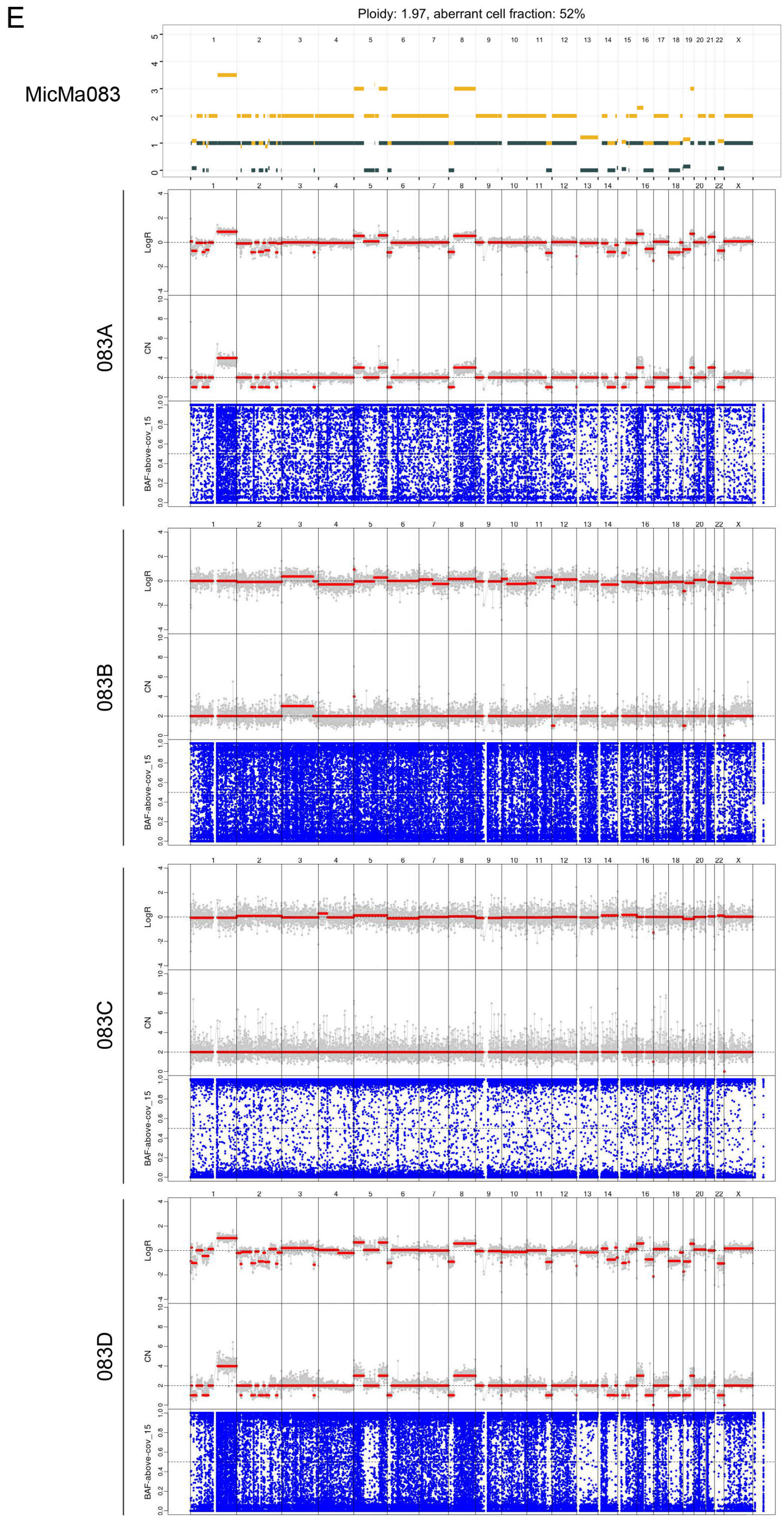
E
Ploidy: 1.97, aberrant cell fraction: 52%
MicMa083
083A
083B
083C
083D
LogR
CN
BAF-above-cov_15

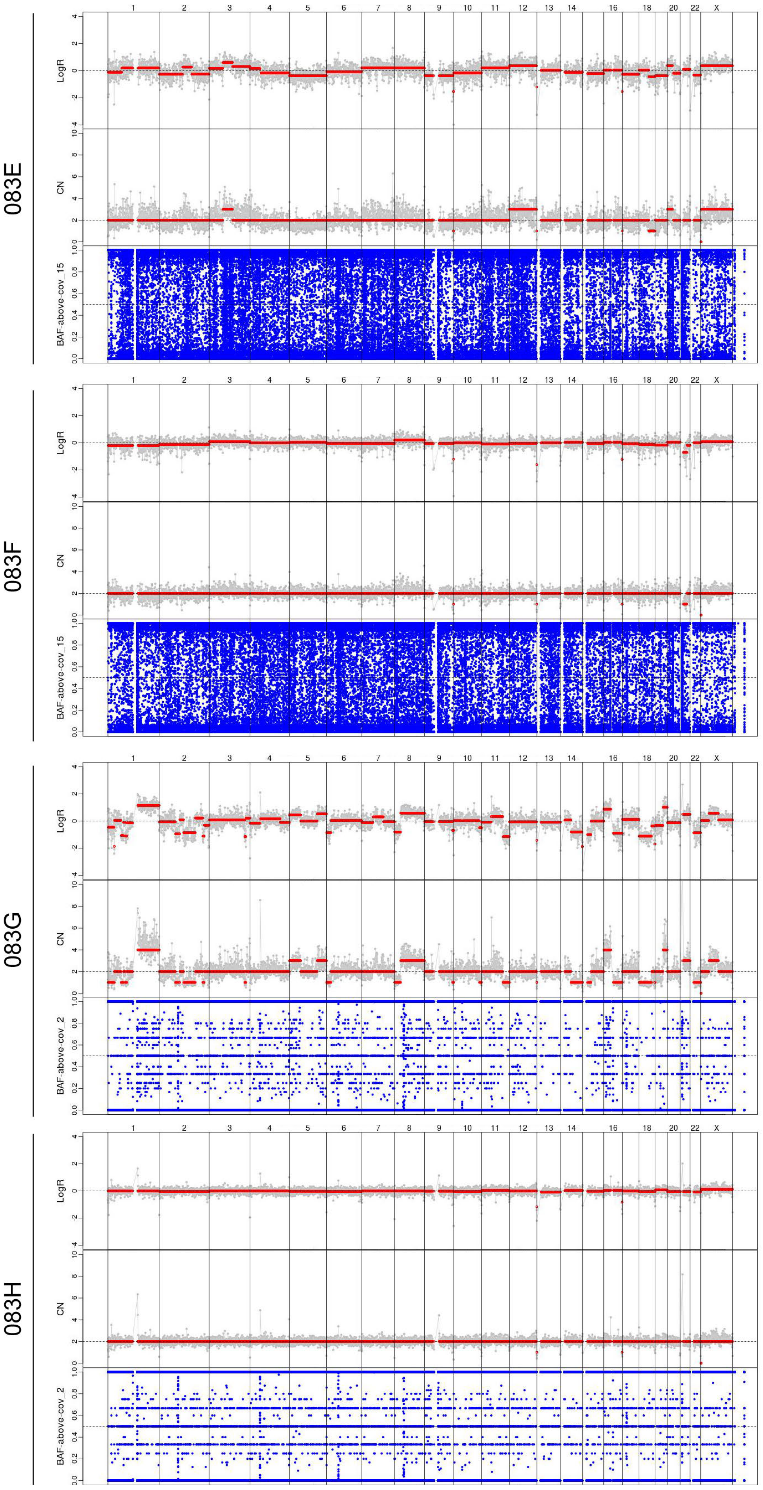
083E
083F
083G
083H
LogR
CN
BAF-above-cov_15
BAF-above-cov_2
1 2 3 4 5 6 7 8 9 10 11 12 13 14 16 18 20 22 X

083I

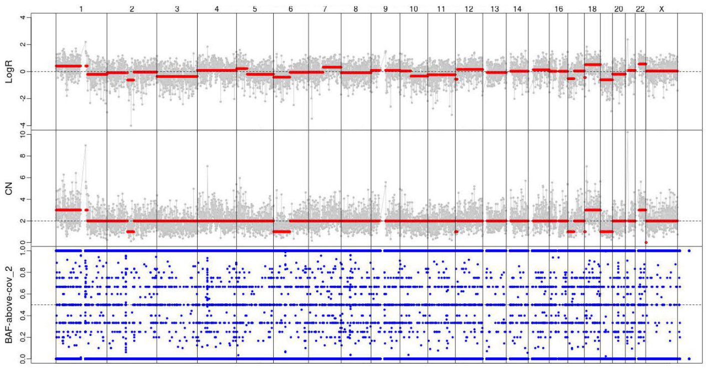

F

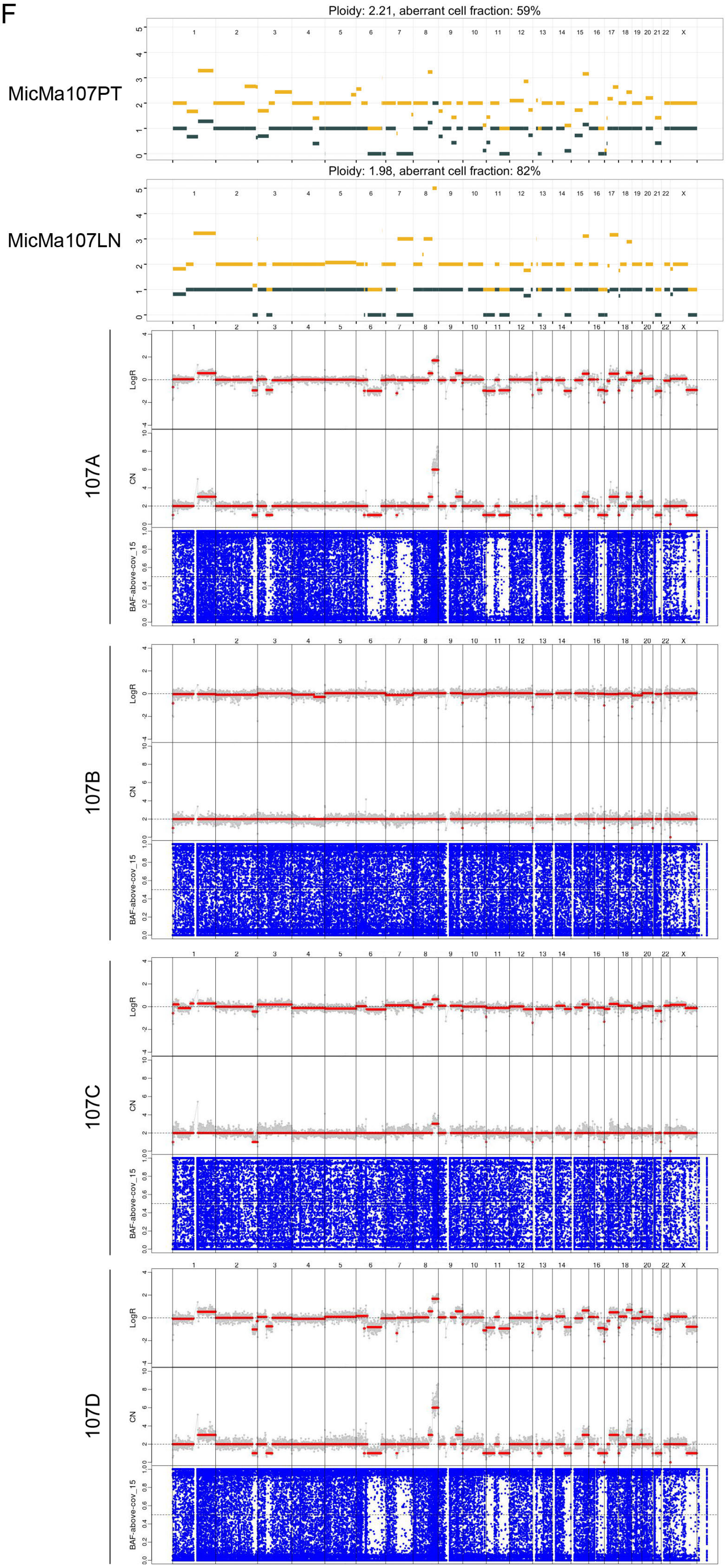

Ploidy: 2.21, aberrant cell fraction: 59%
MicMa107PT
Ploidy: 1.98, aberrant cell fraction: 82%
MicMa107LN
107A
107B
107C
107D
LogR
CN
BAF-above-cov_15

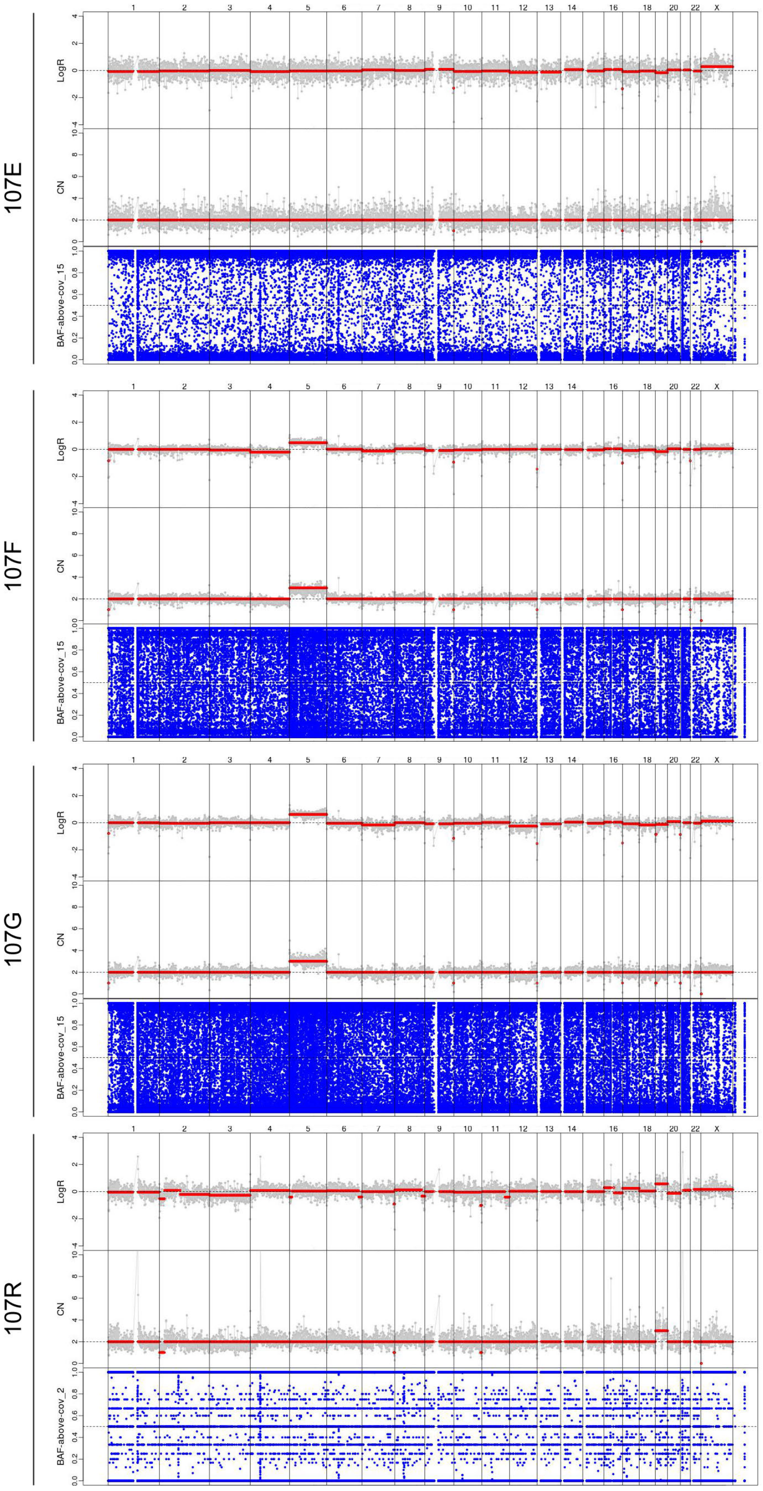
107E
107F
107G
107R
LogR
CN
BAF-above-cov_15
BAF-above-cov_2
1 2 3 4 5 6 7 8 9 10 11 12 13 14 16 18 20 22 X

107S

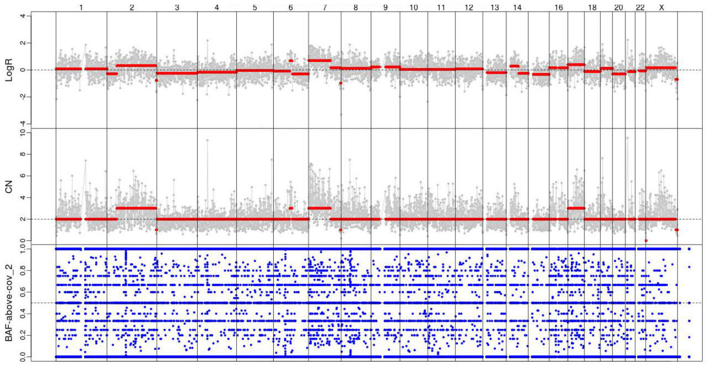

G

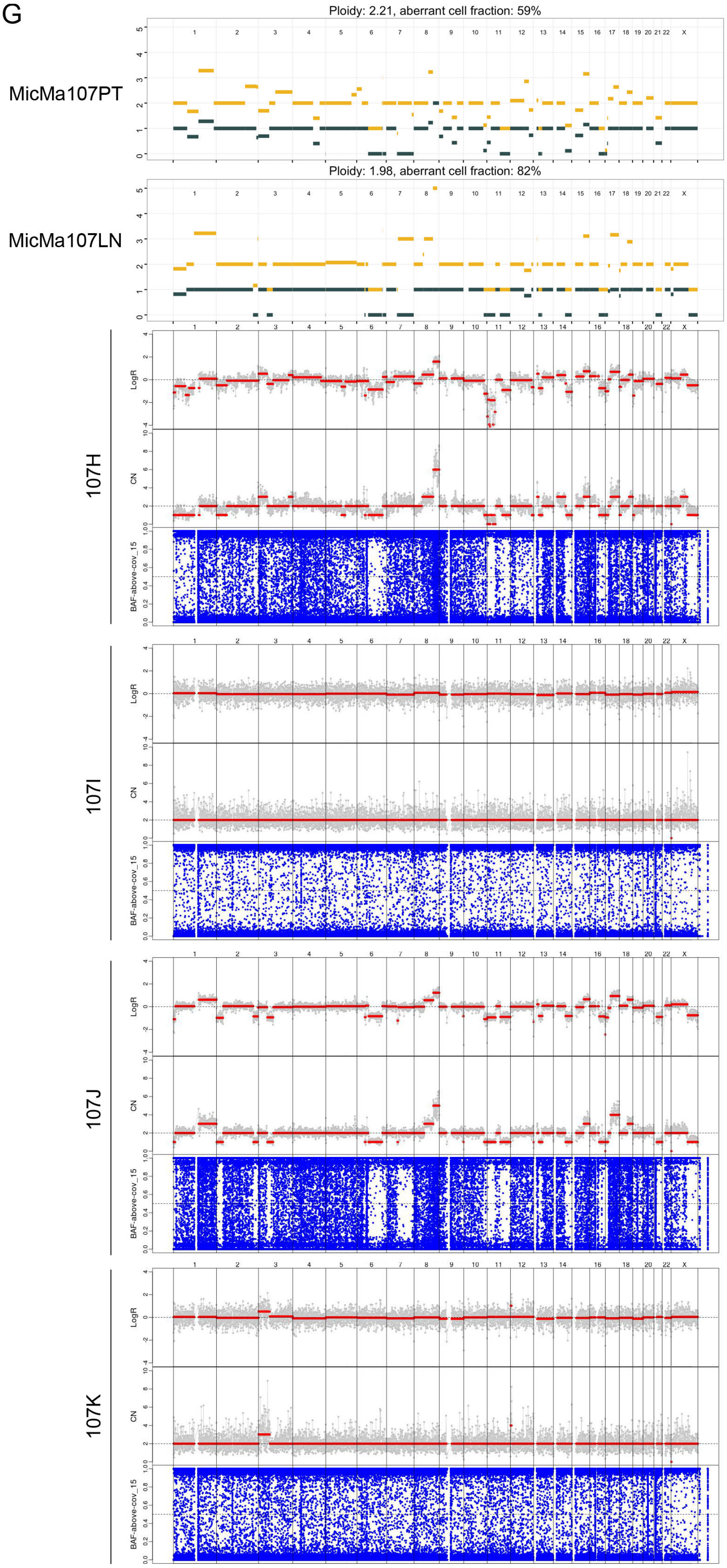

Ploidy: 2.21, aberrant cell fraction: 59%
MicMa107PT
Ploidy: 1.98, aberrant cell fraction: 82%
MicMa107LN
107H
107I
107J
107K
LogR
CN
BAF-above-cov_15

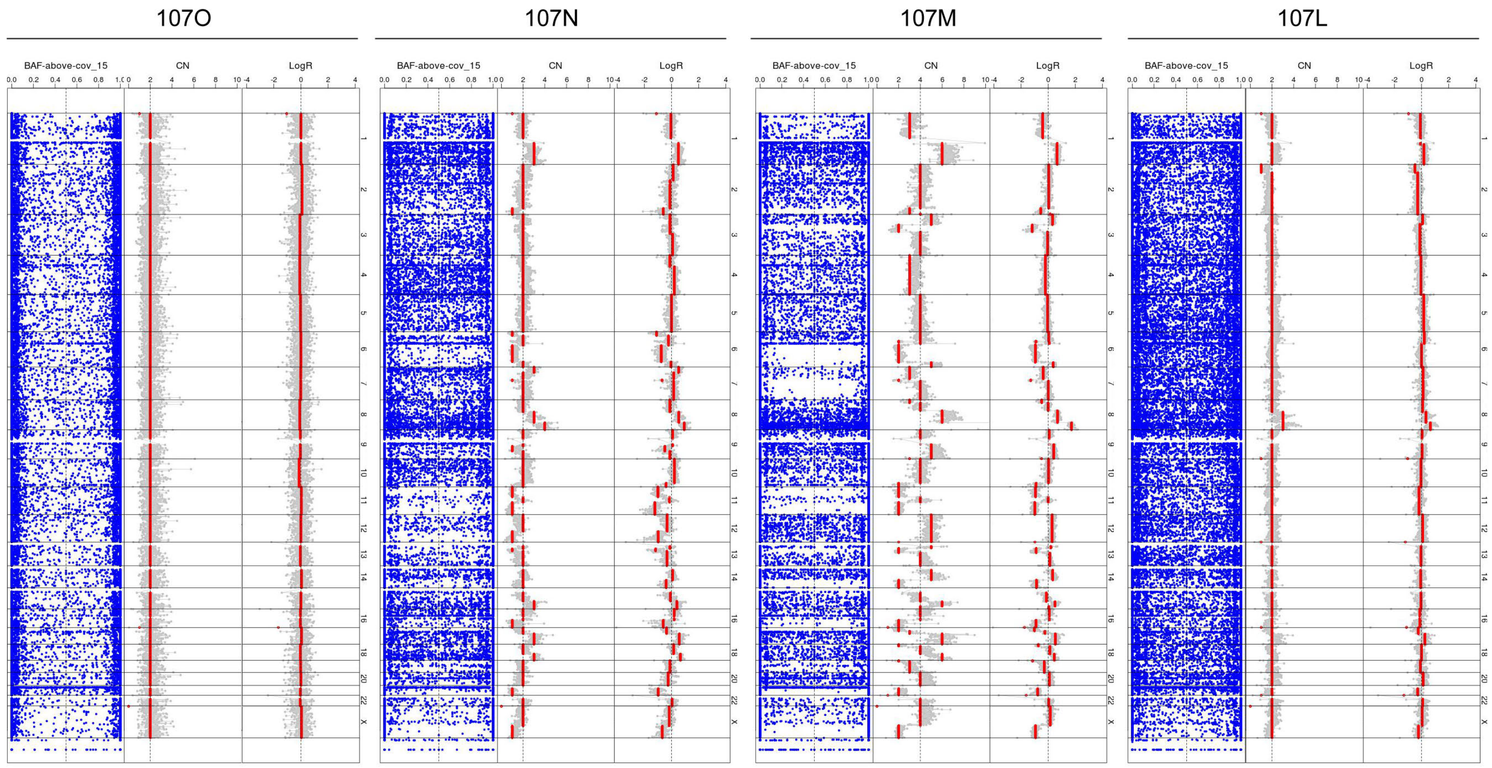
107O
107N
107M
107L
BAF-above-cov_15
CN
LogR

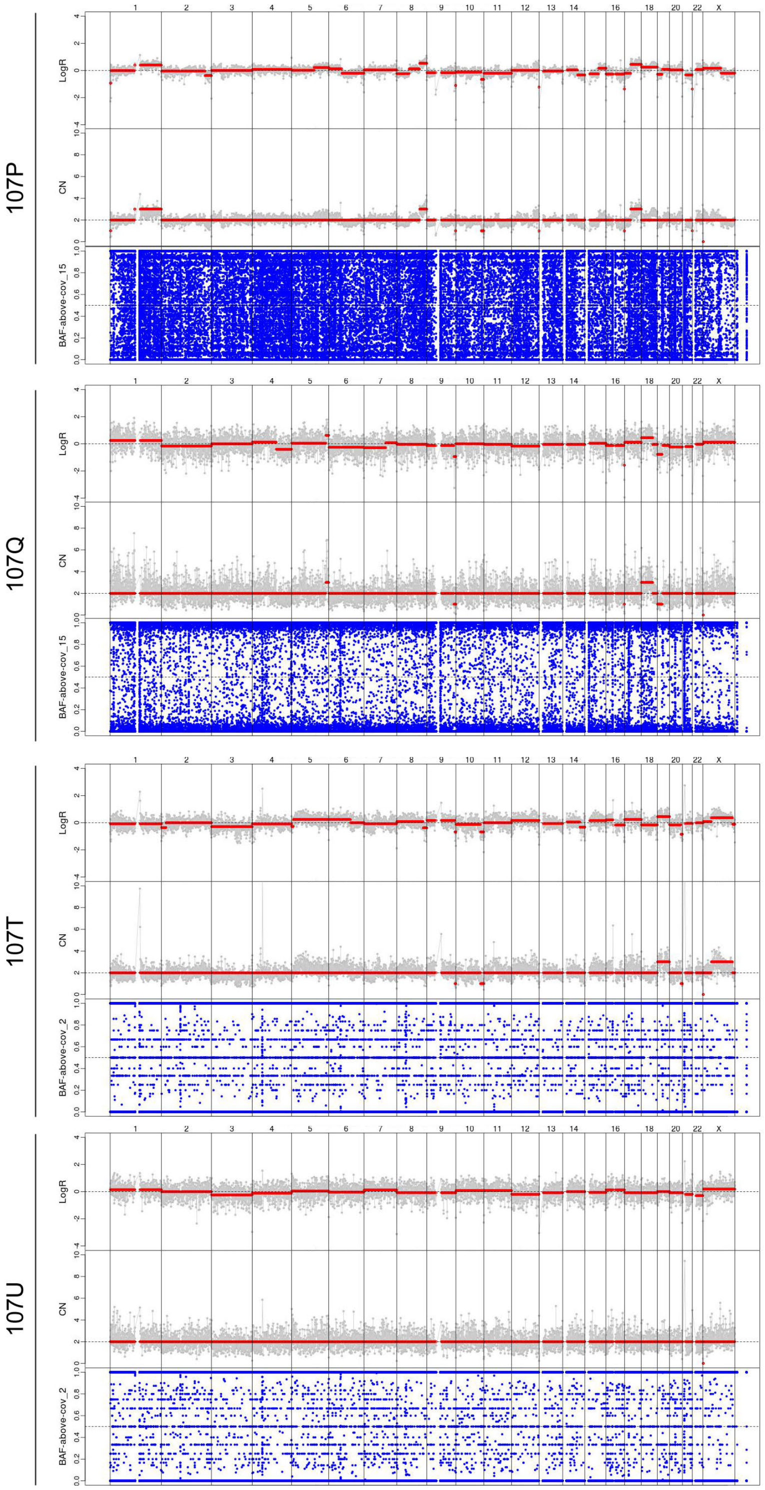
107P
107Q
107T
107U
1 2 3 4 5 6 7 8 9 10 11 12 13 14 16 18 20 22 X
LogR
CN
BAF-above-cov_15
BAF-above-cov_2

Figure S9: Battenberg bulk tumor results for phylogenetically informative segments. Each panel highlights a single node in the phylogenetic tree for the indicated patient (MicMa003, 083 or 107, top) and one or two evidencing segments as derived by Battenberg from the SNP6-array data from the primary tumor or lymph node metastasis (bottom). Battenberg plots represent the logR (top) and fitted segmented allele frequency for the relevant chromosome (middle), and the inferred (sub)clonal copy number state (bottom). The latter plot shows a grid of copy number states for the major (n_{Maj}) and minor (n_{Min}) alleles in the tumor. Blue lines are lines of equal BAF (0, 0.25, 0.5, 0.75 and 1) and red lines of equal logR in the copy number space. Using the estimated tumor purity (ρ) and ploidy (ψ_t) according to $n_{Maj} = \frac{\rho-1+2^{\frac{r}{\gamma}}b(2(1-\rho)+\rho\psi_t)}{\rho}$ and $n_{Min} = \frac{\rho-1+2^{\frac{r}{\gamma}}(1-b)(2(1-\rho)+\rho\psi_t)}{\rho}$, the measured logR ($r$) and B-allele frequency (b) correspond to a single point (plotted as 'X') in this copy number space ([38], Van Loo et al, *Proc Natl Acad Sci U S A*, 2011). The distance from this point to a clonal solution (i.e. the closest grid point) is checked via a standard t-test (black dot if clonal). If $p < 0.05$, a subclonal solution (the two red points) is proposed along one side of the copy number square containing the 'X' ([3], Nik-Zainal et al, *Cell*, 2012). The iso-BAF line corresponding to the segment's BAF (green) crosses two edges of the square, one of which is chosen based on whether the segment's logR is smaller or larger than the square's diagonal of constant logR. The cancer cell fractions are determined by the point of intersection between this edge and the line of equal BAF and are reflected by the size of the red points. Panels detail evidence for the MRCA (A) or the 38% subclone (B) in MicMa003; for the MRCA (C), the 50% (D), the 30% (E) or the 21% (F) subclone in MicMa083; for the MRCA (G), the 88% (H) or other subclones (I) in the primary of MicMa107; and for the MRCA (J), the 89% (K), the 39% (L), the 25% (M), the 16% (N) or other subclones (O) in the lymph node metastasis of MicMa107.

A

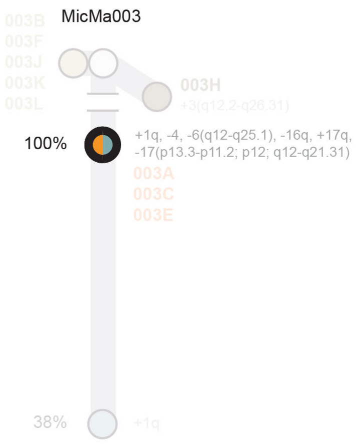
MicMa003
003B
003F
003J
003K
003L
003H
+3(q12.2-q26.31)
100%
+1q, -4, -6(q12-q25.1), -16q, +17q,
-17(p13.3-p11.2; p12; q12-q21.31)
003A
003C
003E
38%
+1q

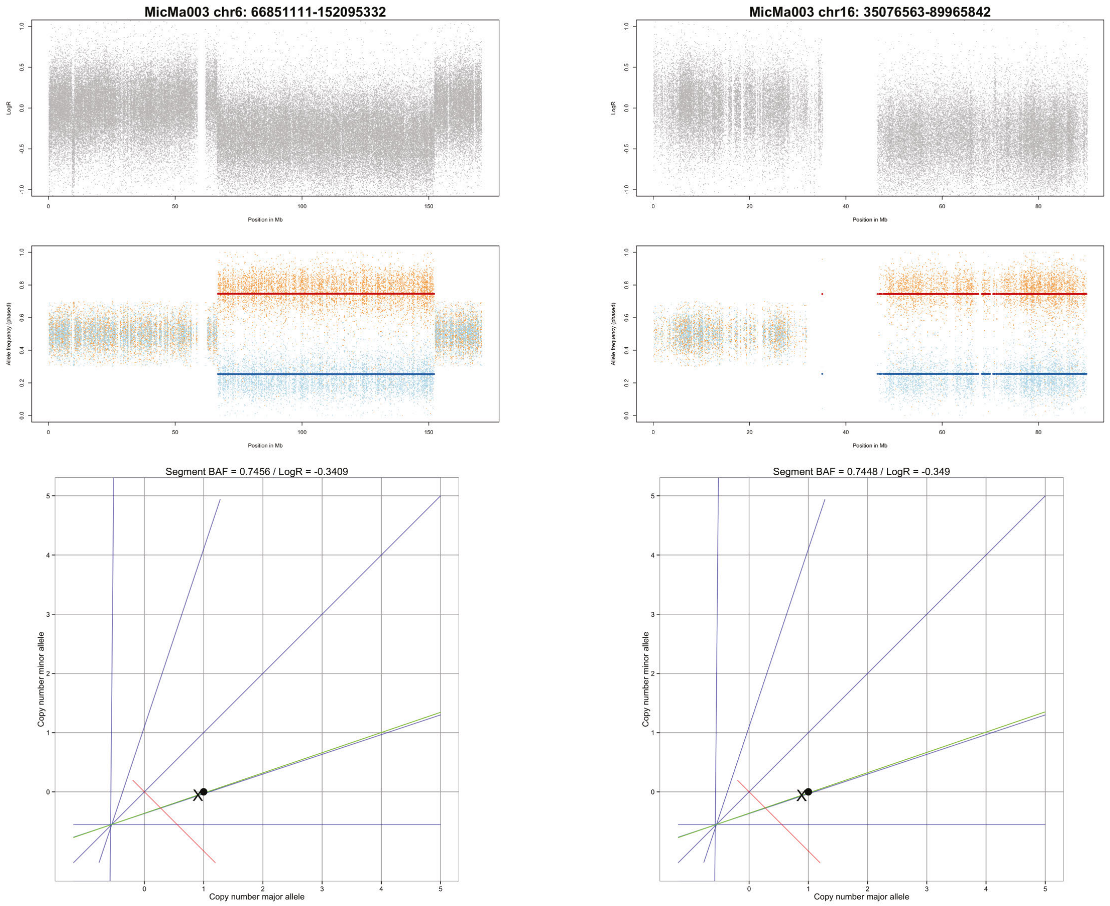
MicMa003 chr6: 66851111-152095332
MicMa003 chr16: 35076563-89965842
LogR
Position in Mb
Allele frequency (phased)
Segment BAF = 0.7456 / LogR = -0.3409
Segment BAF = 0.7448 / LogR = -0.349
Copy number minor allele
Copy number major allele

B

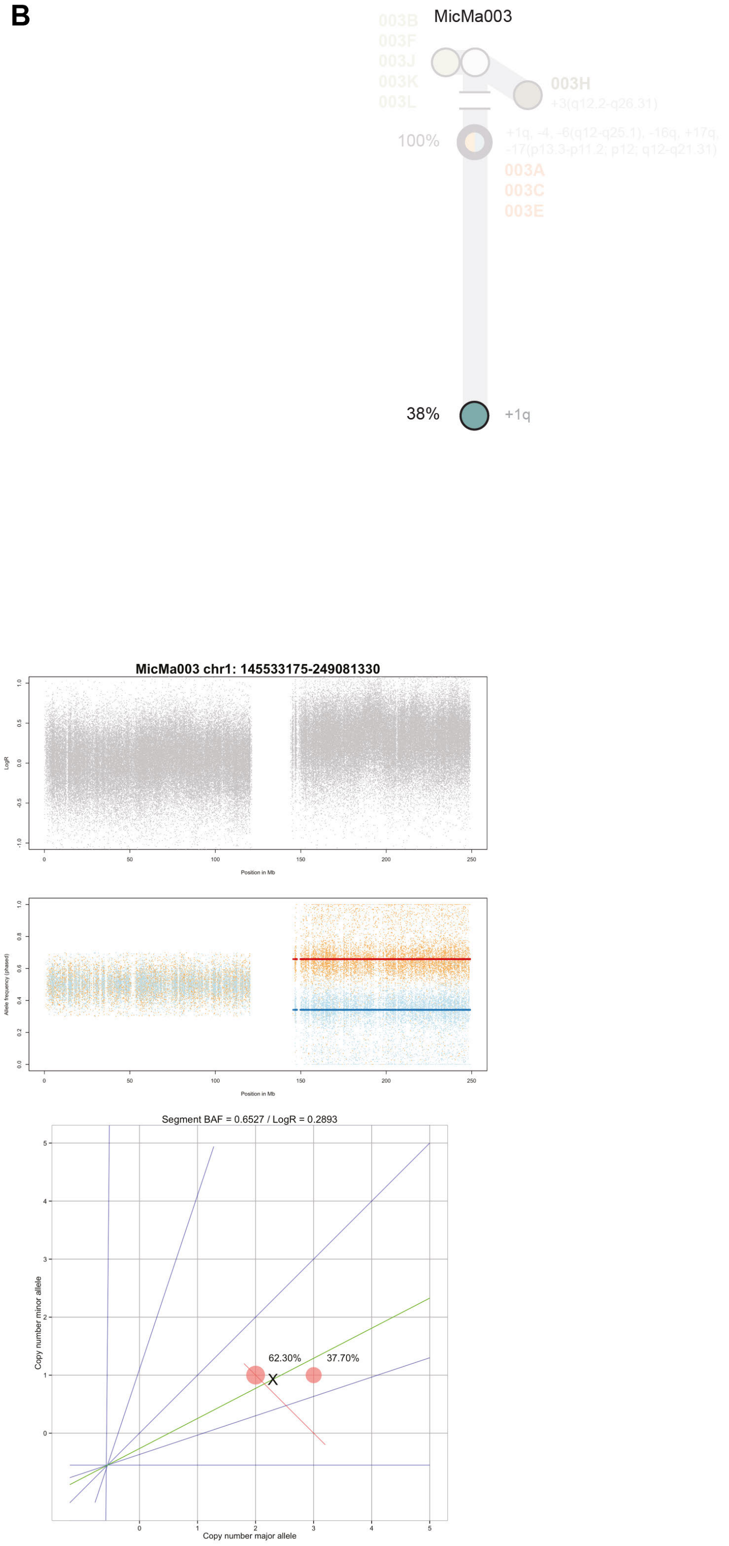
MicMa003
003B
003F
003J
003K
003L
003H
+3(q12.2-q26.31)
100%
+1q, -4, -6(q12-q25.1), -16q, +17q, -17(p13.3-p11.2, p12, q12-q21.31)
003A
003C
003E
38%
+1q
MicMa003 chr1: 145533175-249081330
LogR
Position in Mb
Allele frequency (phased)
Position in Mb
Segment BAF = 0.6527 / LogR = 0.2893
Copy number minor allele
Copy number major allele
62.30%
37.70%
X

C

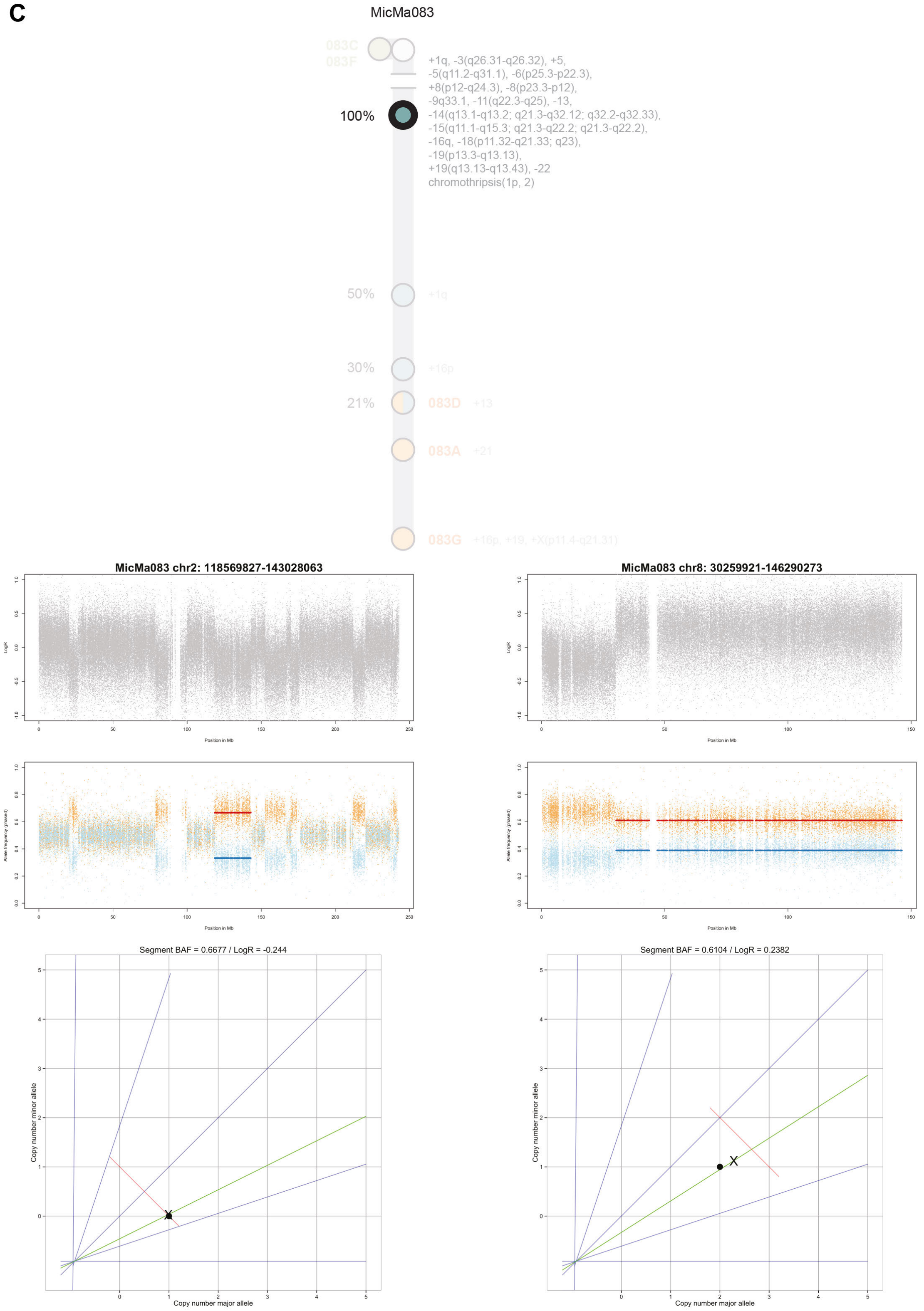
MicMa083
083C
083F
100%
+1q, -3(q26.31-q26.32), +5,
-5(q11.2-q31.1), -6(p25.3-p22.3),
+8(p12-q24.3), -8(p23.3-p12),
-9q33.1, -11(q22.3-q25), -13,
-14(q13.1-q13.2; q21.3-q32.12; q32.2-q32.33),
-15(q11.1-q15.3; q21.3-q22.2; q21.3-q22.2),
-16q, -18(p11.32-q21.33; q23),
-19(p13.3-q13.13),
+19(q13.13-q13.43), -22
chromothripsis(1p, 2)
50%
30%
21%
083D
083A
083G
MicMa083 chr2: 118569827-143028063
MicMa083 chr8: 30259921-146290273
LogR
Position in Mb
Allele frequency (phased)
Segment BAF = 0.6677 / LogR = -0.244
Segment BAF = 0.6104 / LogR = 0.2382
Copy number minor allele
Copy number major allele

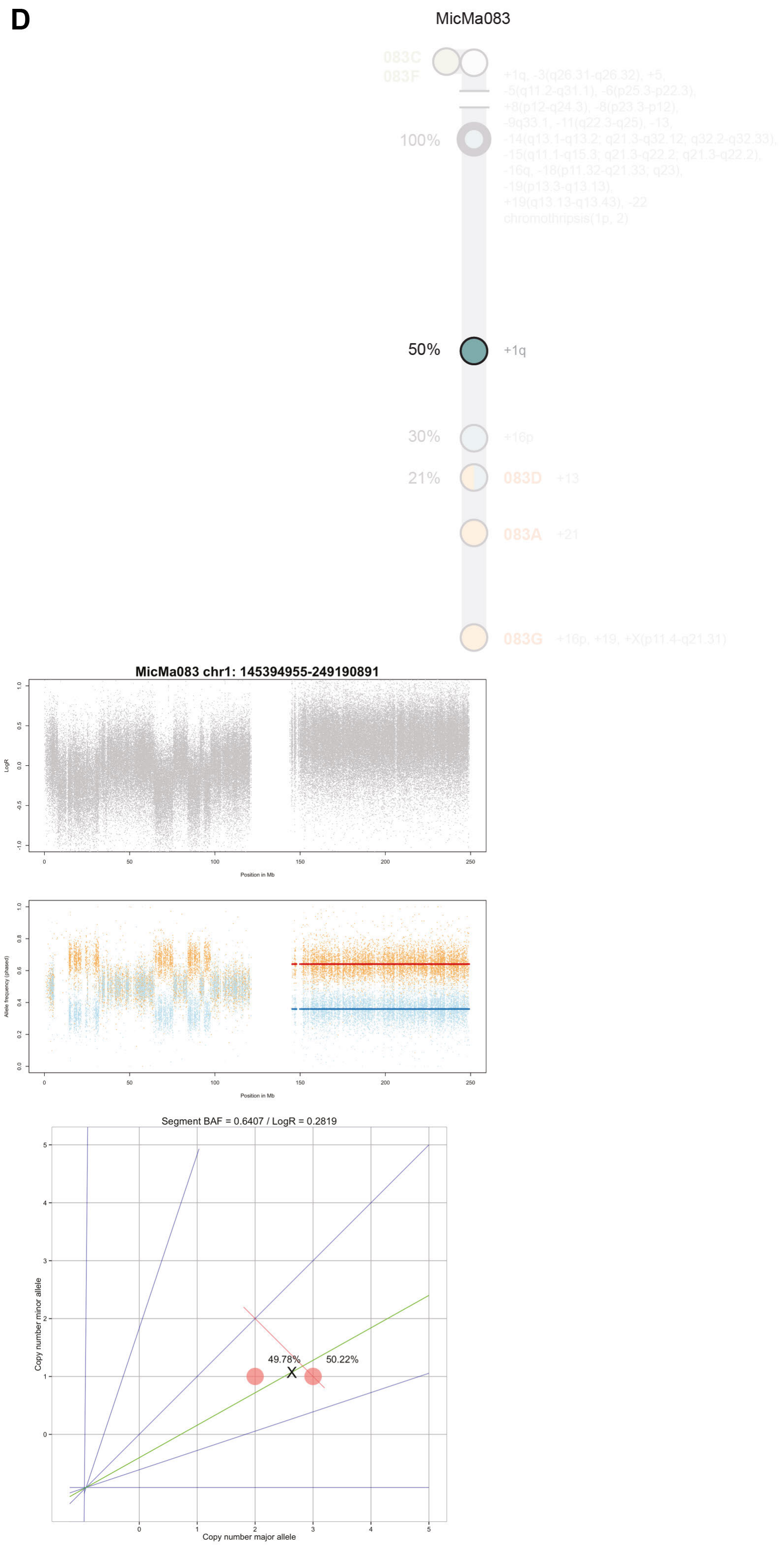
D
MicMa083
100%
50%
+1q
30%
21%
083D
083A
083G
MicMa083 chr1: 145394955-249190891
LogR
Position in Mb
Allele frequency (phased)
Position in Mb
Segment BAF = 0.6407 / LogR = 0.2819
Copy number minor allele
Copy number major allele
49.78%
50.22%

E

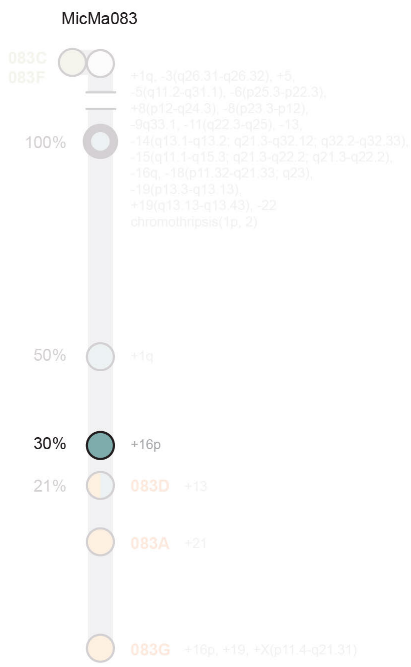
MicMa083
083C
083F
+1q, -3(q26.31-q26.32), +5,
-5(q11.2-q31.1), -6(p25.3-p22.3),
+8(p12-q24.3), -8(p23.3-p12),
-9q33.1, -11(q22.3-q25), -13,
-14(q13.1-q13.2; q21.3-q32.12; q32.2-q32.33),
-15(q11.1-q15.3; q21.3-q22.2; q21.3-q22.2),
-16q, -18(p11.32-q21.33; q23),
-19(p13.3-q13.13),
+19(q13.13-q13.43), -22
chromothripsis(1p, 2)
100%
50%
+1q
30%
+16p
21%
083D
+13
083A
+21
083G
+16p, +19, +X(p11.4-q21.31)

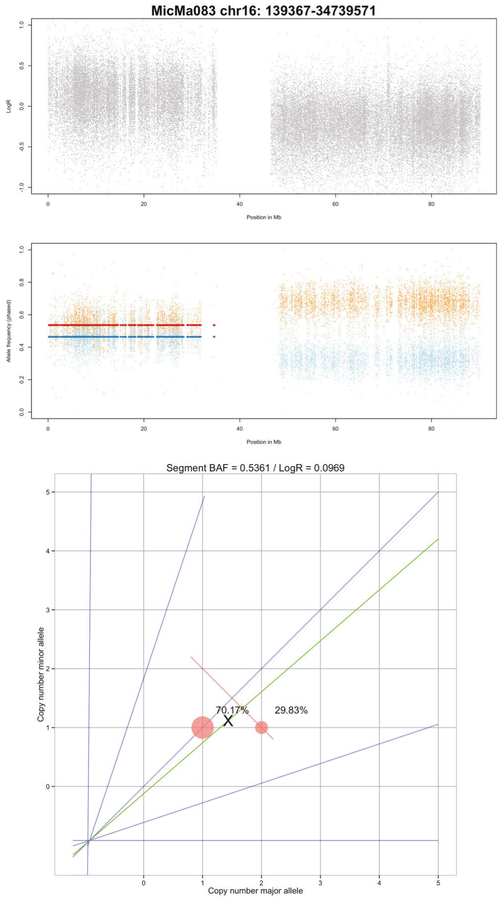
MicMa083 chr16: 139367-34739571
LogR
Position in Mb
Allele frequency (phased)
Position in Mb
Segment BAF = 0.5361 / LogR = 0.0969
70.17%
X
29.83%
Copy number minor allele
Copy number major allele

F

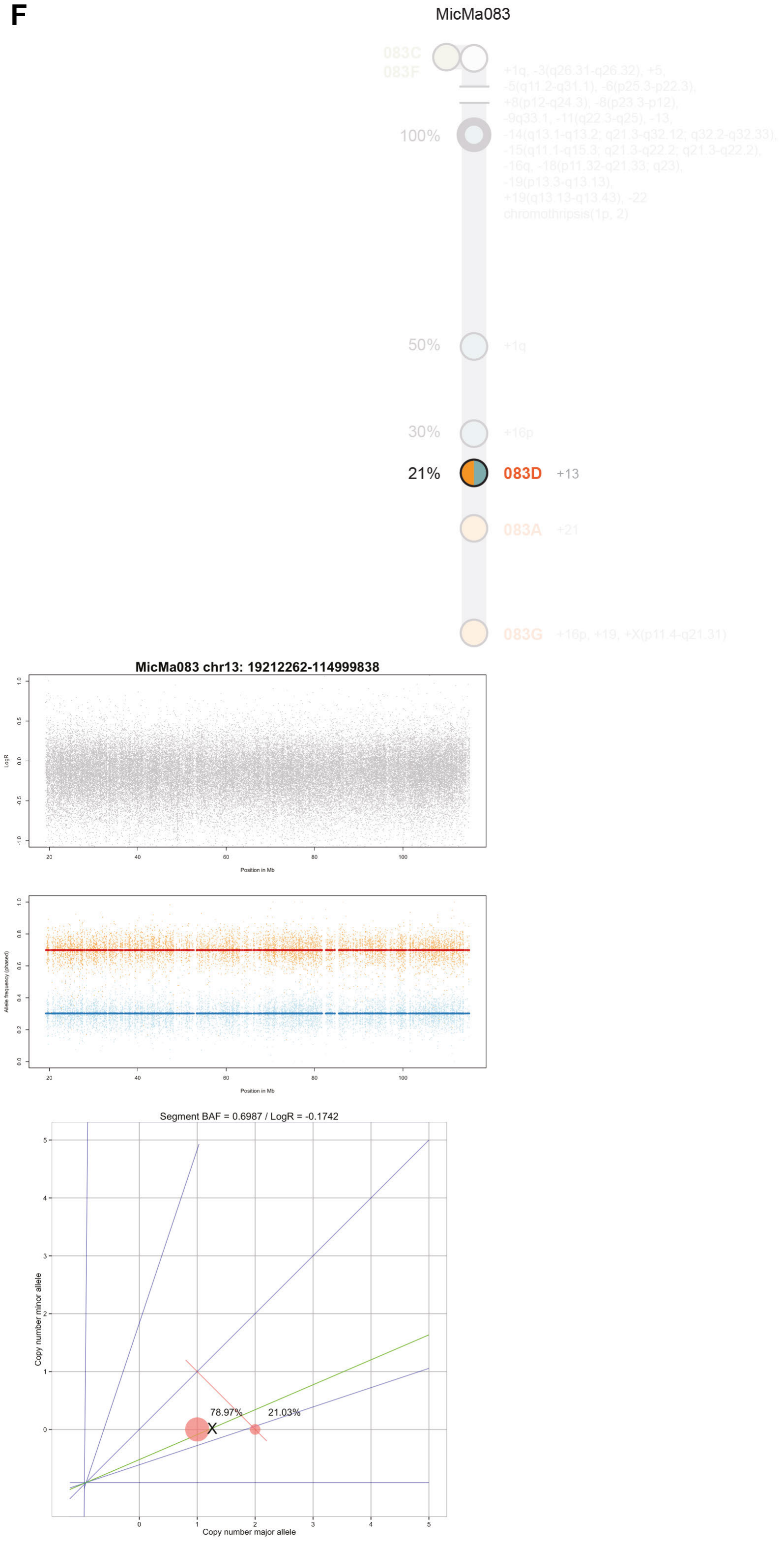
MicMa083
083C
083F
+1q, -3(q26.31-q26.32), +5,
-5(q11.2-q31.1), -6(p25.3-p22.3),
+8(p12-q24.3), -8(p23.3-p12),
-9q33.1, -11(q22.3-q25), -13,
-14(q13.1-q13.2; q21.3-q32.12; q32.2-q32.33),
-15(q11.1-q15.3; q21.3-q22.2; q21.3-q22.2),
-16q, -18(p11.32-q21.33; q23),
-19(p13.3-q13.13),
+19(q13.13-q13.43), -22
chromothripsis(1p, 2)
100%
50%
+1q
30%
+16p
21%
083D
+13
083A
+21
083G
+16p, +19, +X(p11.4-q21.31)
MicMa083 chr13: 19212262-114999838
LogR
Position in Mb
Allele frequency (phased)
Position in Mb
Segment BAF = 0.6987 / LogR = -0.1742
78.97%
21.03%
Copy number minor allele
Copy number major allele

G

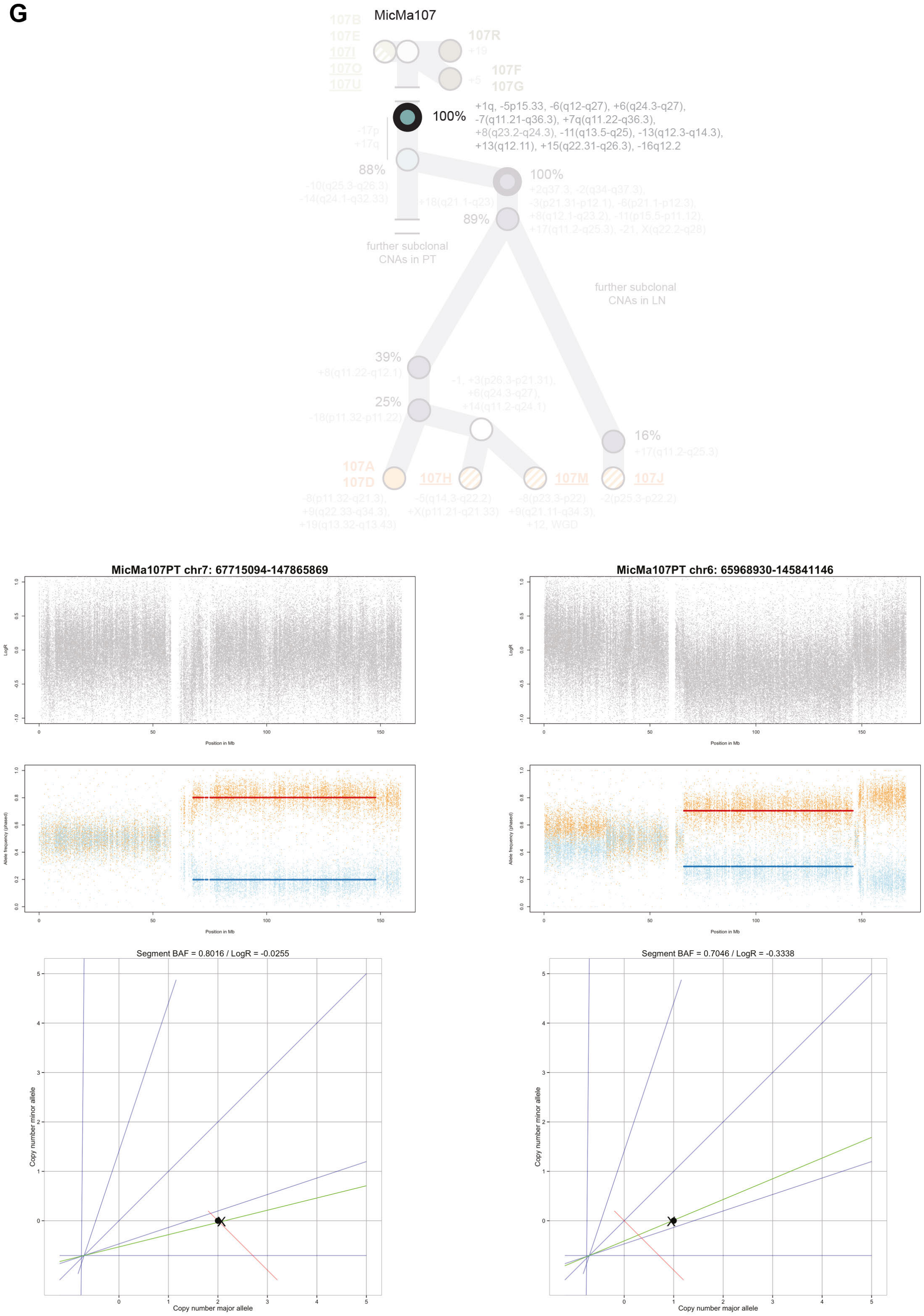
MicMa107
100%
+1q, -5p15.33, -6(q12-q27), +6(q24.3-q27),
-7(q11.21-q36.3), +7q(q11.22-q36.3),
+8(q23.2-q24.3), -11(q13.5-q25), -13(q12.3-q14.3),
+13(q12.11), +15(q22.31-q26.3), -16q12.2
88%
100%
89%
further subclonal CNAs in PT
further subclonal CNAs in LN
39%
25%
16%
MicMa107PT chr7: 67715094-147865869
MicMa107PT chr6: 65968930-145841146
Segment BAF = 0.8016 / LogR = -0.0255
Segment BAF = 0.7046 / LogR = -0.3338
Copy number minor allele
Copy number major allele

H

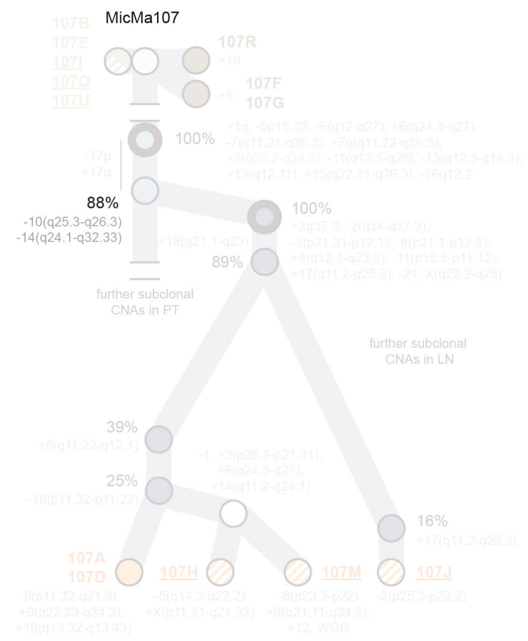
MicMa107
100%
88%
-10(q25.3-q26.3)
-14(q24.1-q32.33)
100%
89%
further subclonal CNAs in PT
further subclonal CNAs in LN
39%
25%
16%

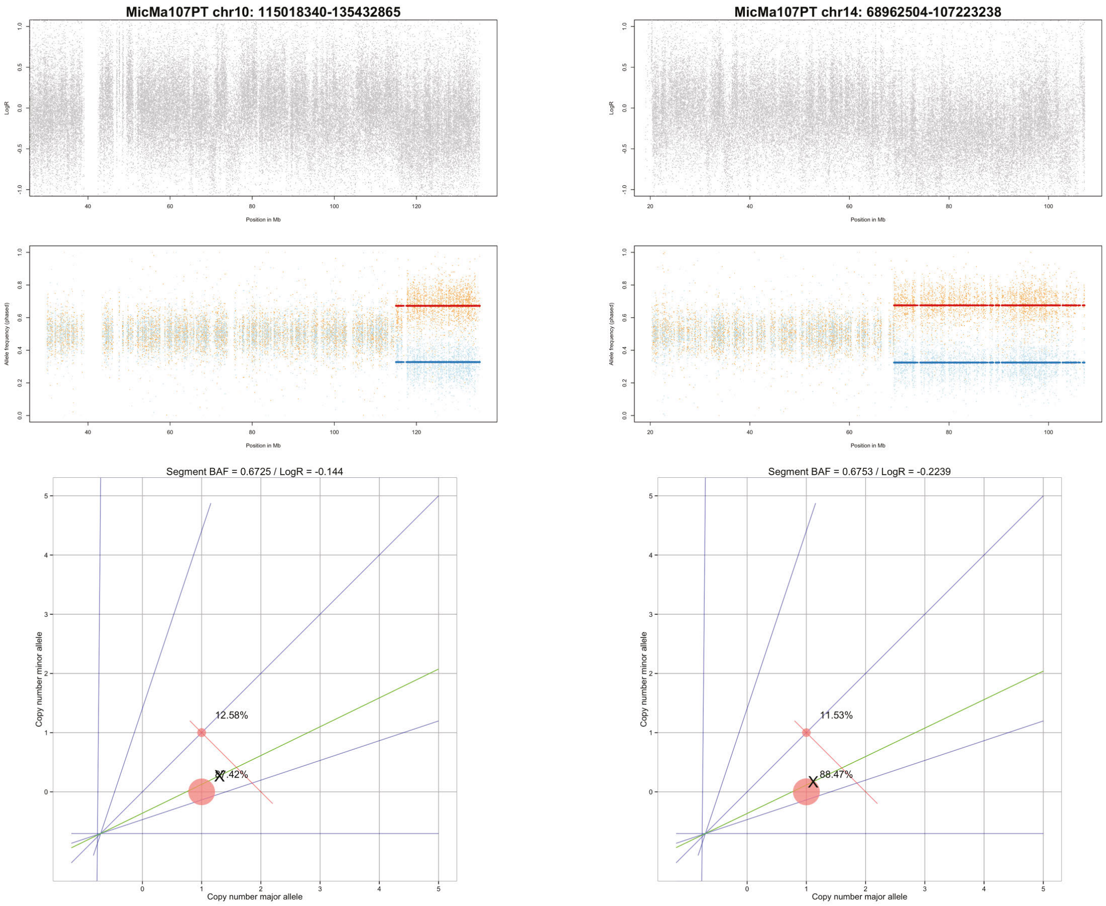
MicMa107PT chr10: 115018340-135432865
MicMa107PT chr14: 68962504-107223238
LogR
Position in Mb
Allele frequency (phased)
Segment BAF = 0.6725 / LogR = -0.144
Segment BAF = 0.6753 / LogR = -0.2239
Copy number minor allele
Copy number major allele
12.58%
11.53%
88.47%

I

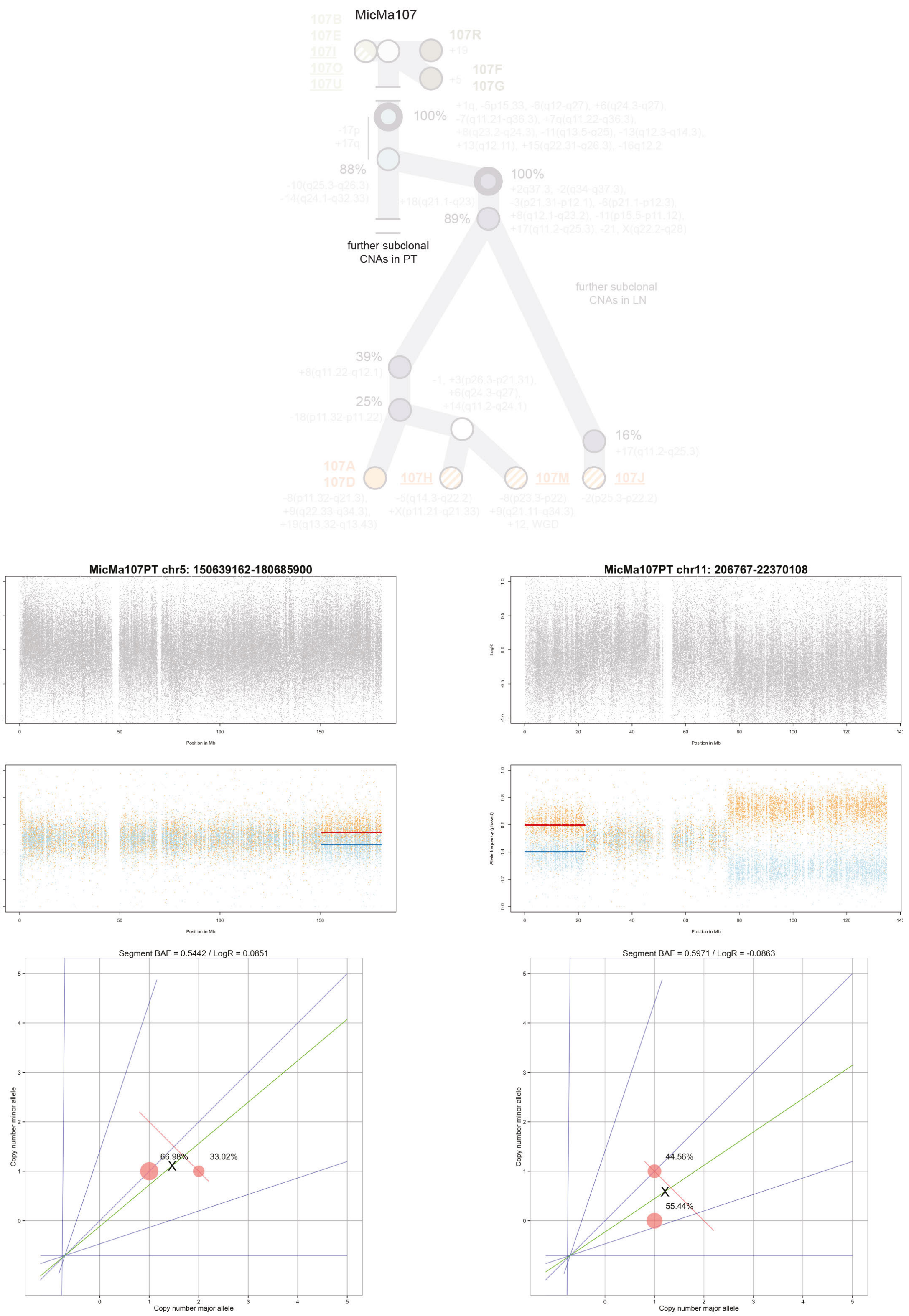

MicMa107
107B
107E
107I
107O
107U
107R
+19
+5
107F
107G
100%
-17p
+17q
88%
100%
89%
further subclonal
CNAs in PT
further subclonal
CNAs in LN
39%
25%
16%
107A
107D
107H
107M
107J
MicMa107PT chr5: 150639162-180685900
MicMa107PT chr11: 206767-22370108
LogR
Position in Mb
Allele frequency (phased)
Segment BAF = 0.5442 / LogR = 0.0851
Segment BAF = 0.5971 / LogR = -0.0863
66.98%
33.02%
44.56%
55.44%
X
Copy number minor allele
Copy number major allele

J

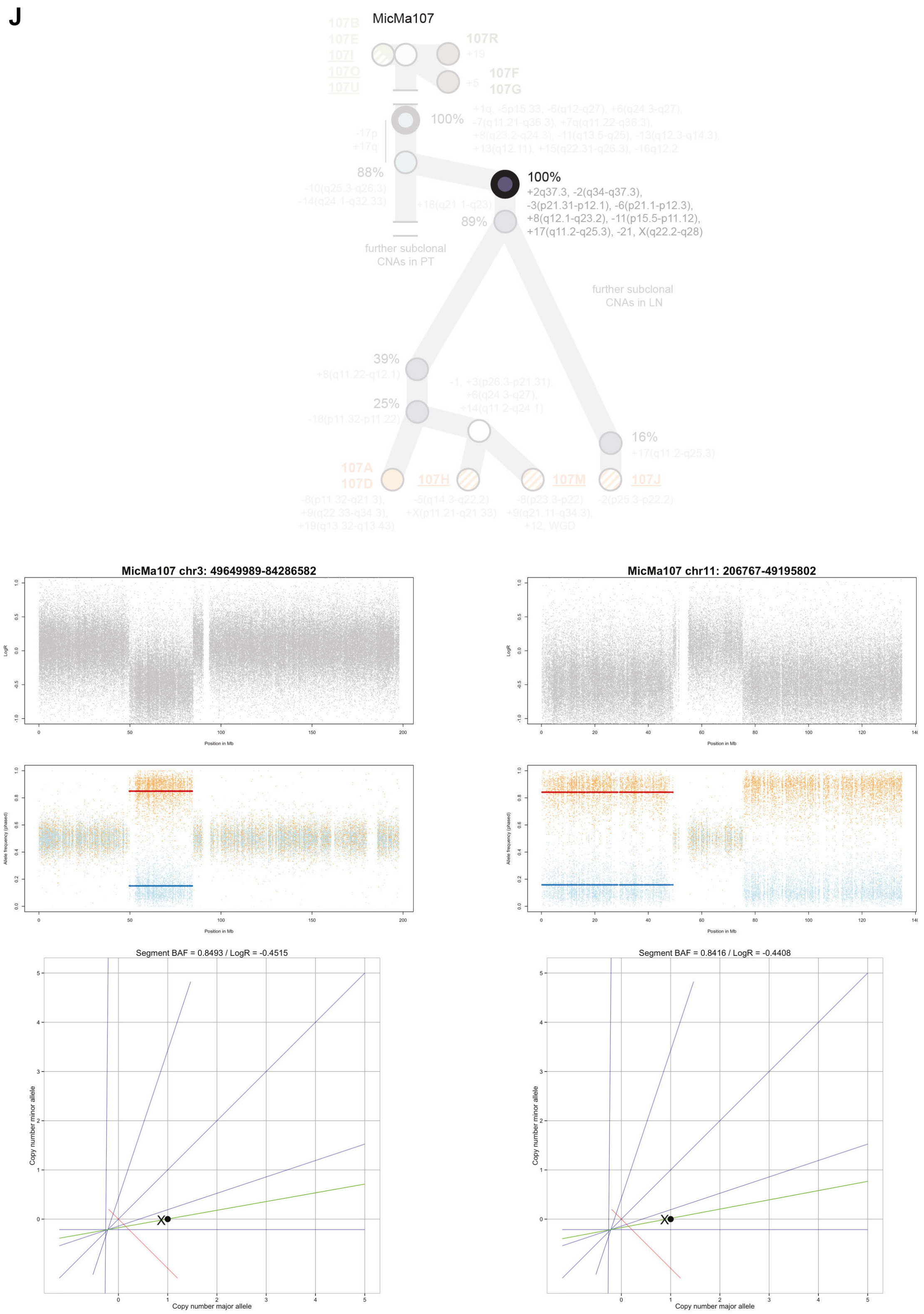
MicMa107
100%
+2q37.3, -2(q34-q37.3),
-3(p21.31-p12.1), -6(p21.1-p12.3),
+8(q12.1-q23.2), -11(p15.5-p11.12),
+17(q11.2-q25.3), -21, X(q22.2-q28)
further subclonal CNAs in PT
further subclonal CNAs in LN
MicMa107 chr3: 49649989-84286582
MicMa107 chr11: 206767-49195802
Segment BAF = 0.8493 / LogR = -0.4515
Segment BAF = 0.8416 / LogR = -0.4408
Copy number minor allele
Copy number major allele

K

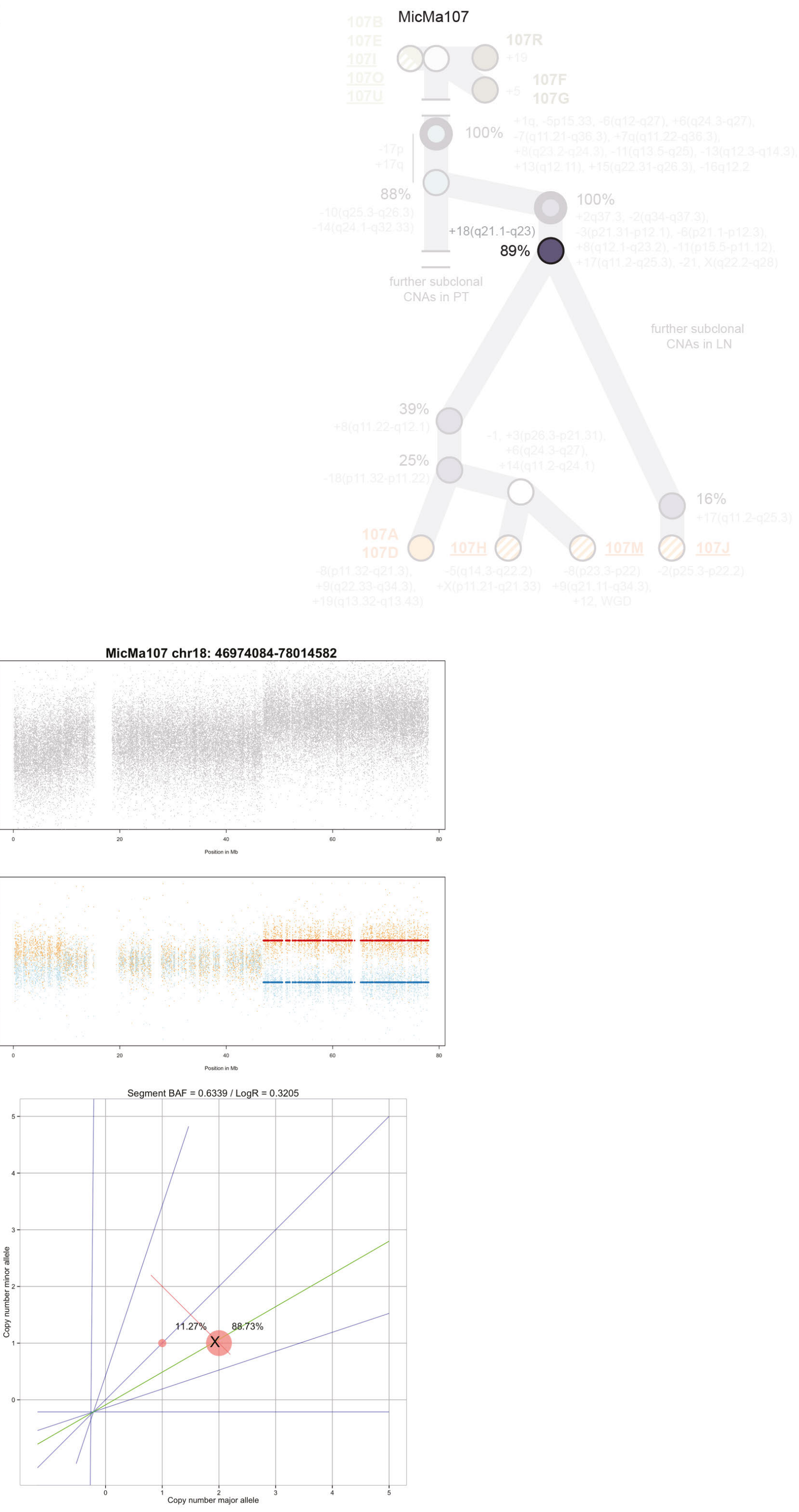

MicMa107
107B
107E
107I
107O
107U
107R
+19
+5
107F
107G
100%
-17p
+17q
88%
100%
+18(q21.1-q23)
89%
further subclonal
CNAs in PT
further subclonal
CNAs in LN
39%
25%
16%
107A
107D
107H
107M
107J
MicMa107 chr18: 46974084-78014582
LogR
Position in Mb
Allele frequency (phased)
Position in Mb
Segment BAF = 0.6339 / LogR = 0.3205
11.27%
88.73%
Copy number minor allele
Copy number major allele

L

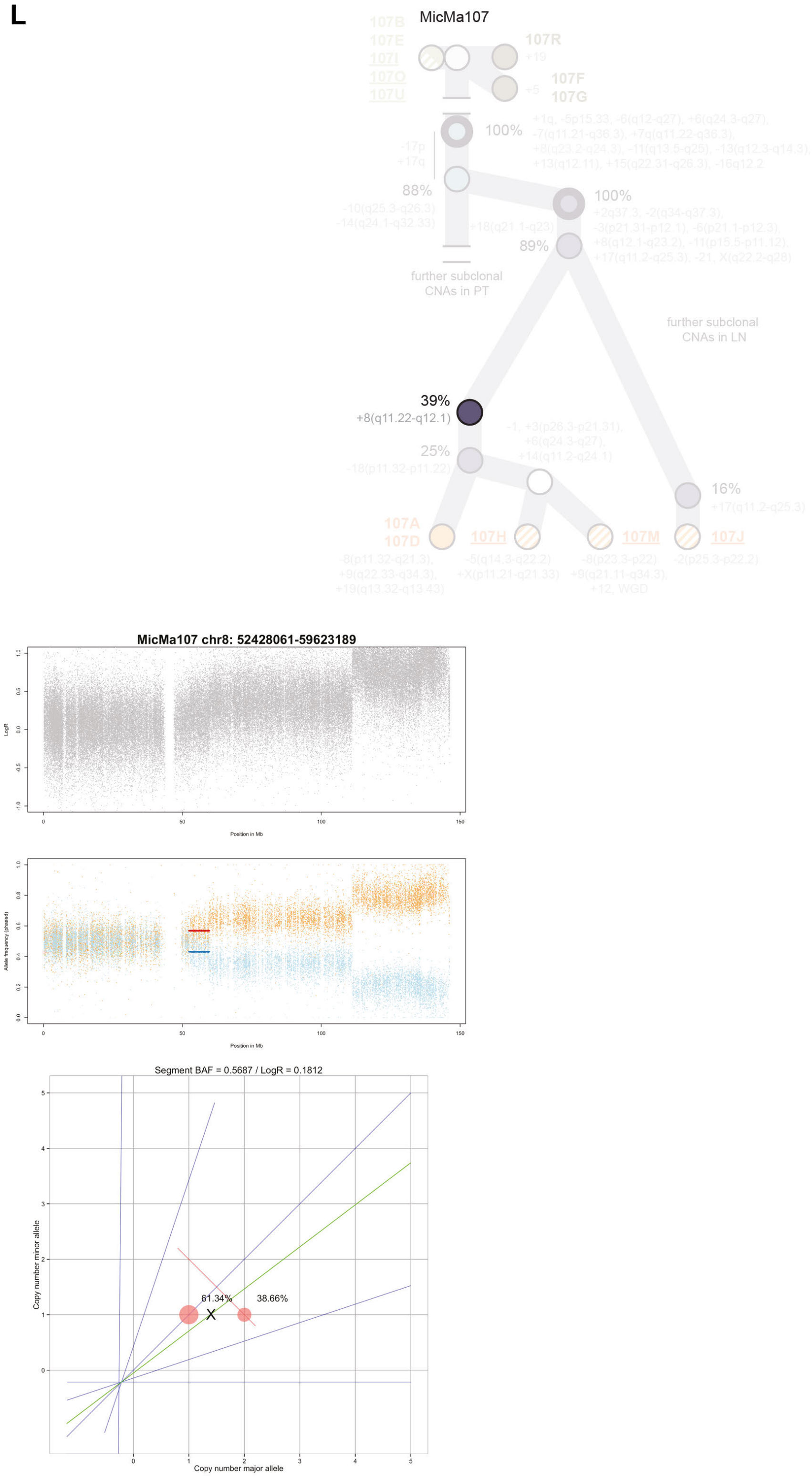
MicMa107
107R
107F
107G
100%
88%
100%
89%
further subclonal CNAs in PT
further subclonal CNAs in LN
39%
+8(q11.22-q12.1)
25%
16%
107A
107D
107H
107M
107J
MicMa107 chr8: 52428061-59623189
LogR
Position in Mb
Allele frequency (phased)
Position in Mb
Segment BAF = 0.5687 / LogR = 0.1812
61.34%
38.66%
X
Copy number minor allele
Copy number major allele

M

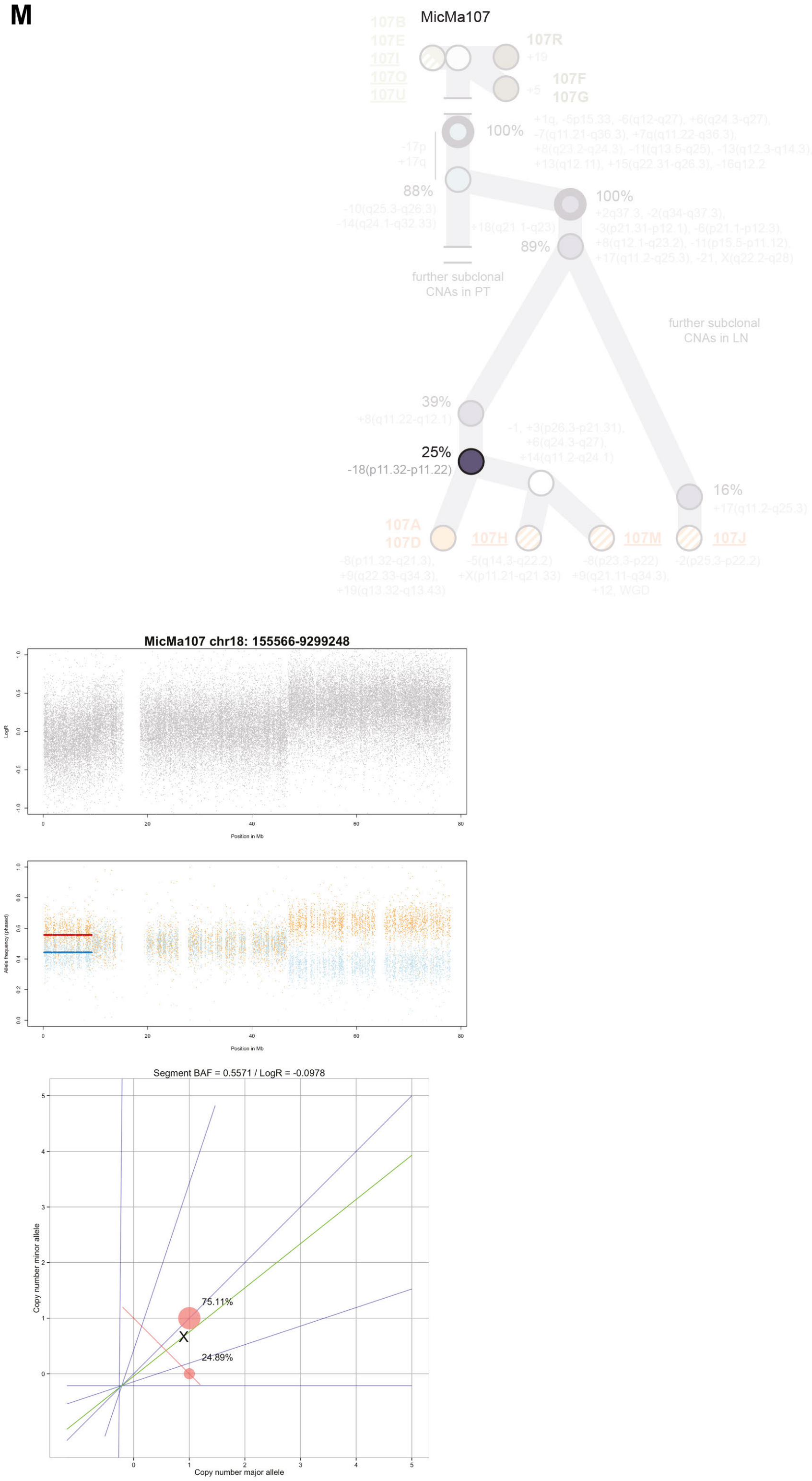
MicMa107
100%
88%
100%
89%
further subclonal
CNAs in PT
further subclonal
CNAs in LN
39%
25%
-18(p11.32-p11.22)
16%
MicMa107 chr18: 155566-9299248
LogR
Position in Mb
Allele frequency (phased)
Position in Mb
Segment BAF = 0.5571 / LogR = -0.0978
75.11%
24.89%
Copy number minor allele
Copy number major allele

N

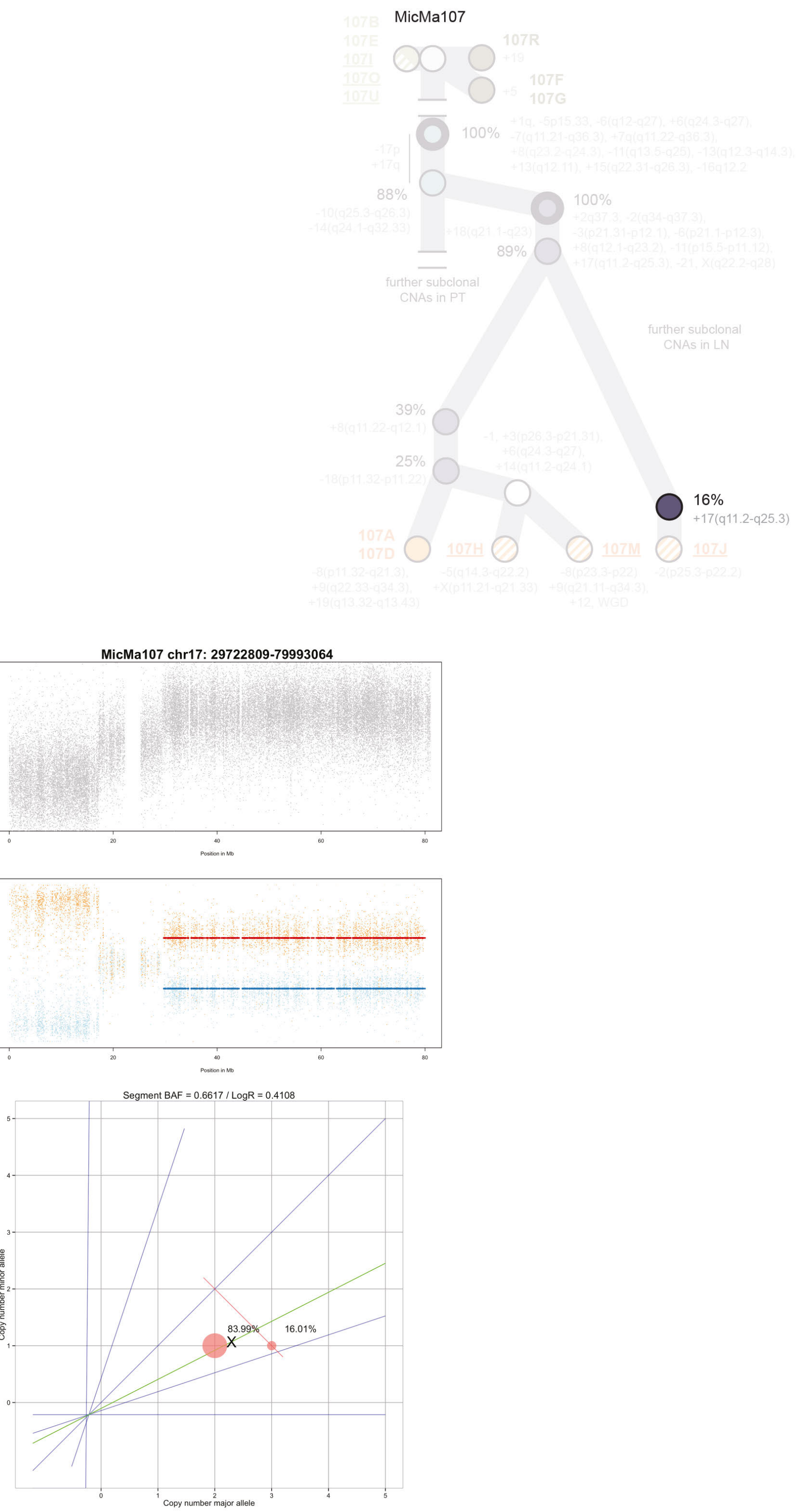
MicMa107
100%
88%
100%
89%
further subclonal CNAs in PT
further subclonal CNAs in LN
39%
25%
16%
+17(q11.2-q25.3)
MicMa107 chr17: 29722809-79993064
LogR
Position in Mb
Allele frequency (phased)
Position in Mb
Segment BAF = 0.6617 / LogR = 0.4108
83.99%
16.01%
X
Copy number minor allele
Copy number major allele

O

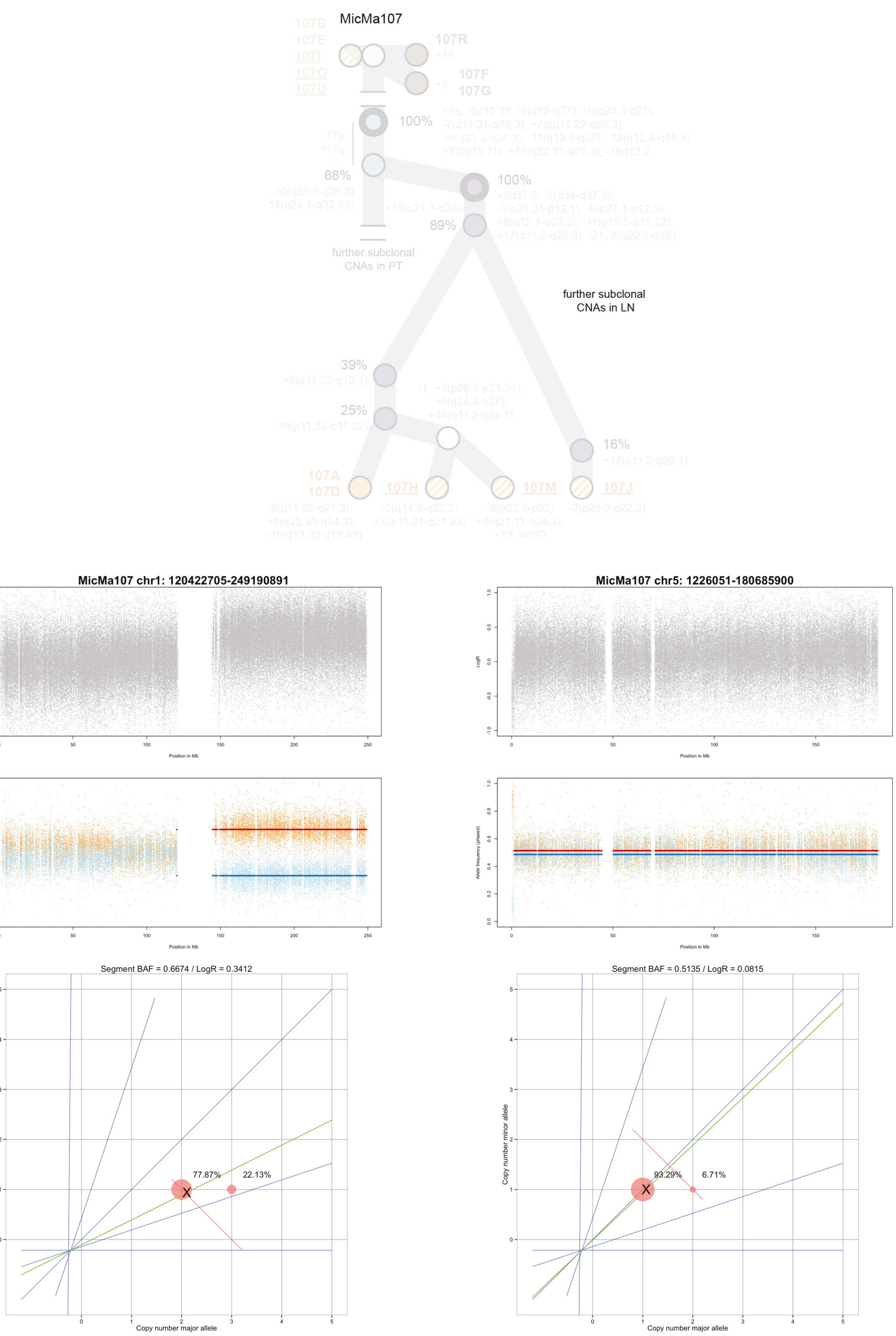
MicMa107
107B
107E
107I
107O
107U
107R
+19
+5
107F
107G
100%
88%
100%
89%
further subclonal CNAs in PT
further subclonal CNAs in LN
39%
25%
16%
107A
107D
107H
107M
107J
MicMa107 chr1: 120422705-249190891
MicMa107 chr5: 1226051-180685900
LogR
Position in Mb
Allele frequency (phased)
Segment BAF = 0.6674 / LogR = 0.3412
Segment BAF = 0.5135 / LogR = 0.0815
77.87%
22.13%
93.29%
6.71%
Copy number minor allele
Copy number major allele

Figure S10: Flowchart of bulk exome sequence analysis. The steps schematically outline our workflow to detect somatic mutations in the bulk tumor exomes and filter out putative false positives.

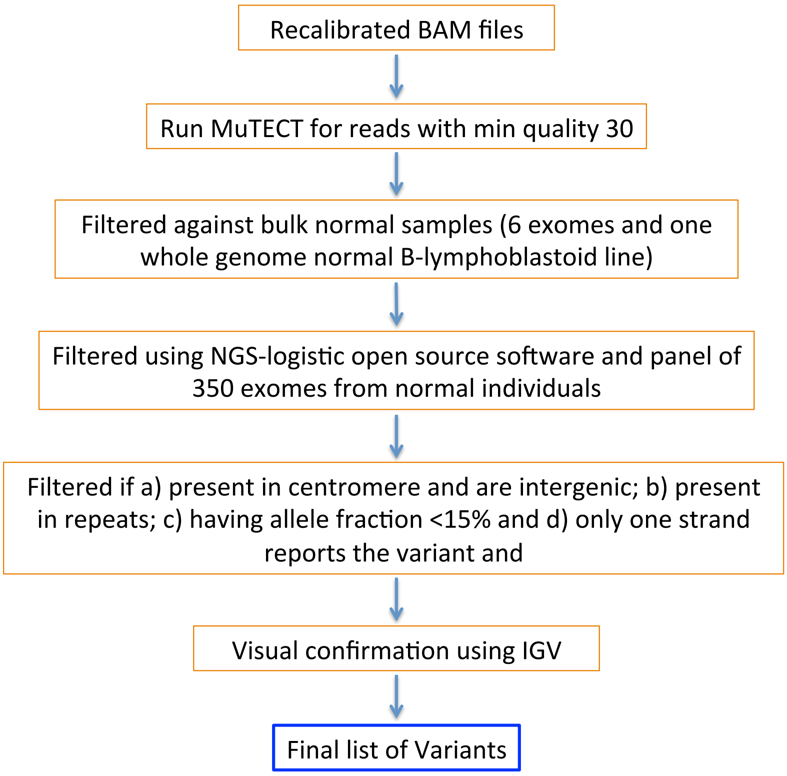

Supplement: Additional file 1: — Supplementary Figures S1–S10. (PDF 23 mb) [file 13059_2016_1109_MOESM1_ESM.pdf]
